# Meta-Analysis of Genome-Wide Scans for Total Body BMD in Children and Adults Reveals Allelic Heterogeneity and Age-Specific Effects at the *WNT16* Locus

**DOI:** 10.1371/journal.pgen.1002718

**Published:** 2012-07-05

**Authors:** Carolina Medina-Gomez, John P. Kemp, Karol Estrada, Joel Eriksson, Jeff Liu, Sjur Reppe, David M. Evans, Denise H. M. Heppe, Liesbeth Vandenput, Lizbeth Herrera, Susan M. Ring, Claudia J. Kruithof, Nicholas J. Timpson, M. Carola Zillikens, Ole K. Olstad, Hou-Feng Zheng, J. Brent Richards, Beate St. Pourcain, Albert Hofman, Vincent W. V. Jaddoe, George Davey Smith, Mattias Lorentzon, Kaare M. Gautvik, André G. Uitterlinden, Robert Brommage, Claes Ohlsson, Jonathan H. Tobias, Fernando Rivadeneira

**Affiliations:** 1 Department of Internal Medicine, Erasmus University Medical Center, Rotterdam, The Netherlands; 2 The Generation R Study Group, Erasmus University Medical Center, Rotterdam, The Netherlands; 3 Department of Epidemiology, Erasmus University Medical Center, Rotterdam, The Netherlands; 4 Netherlands Genomics Initiative (NGI)–sponsored Netherlands Consortium for Healthy Aging (NCHA), Rotterdam, The Netherlands; 5 MRC CAiTE Centre, School of Social and Community Medicine, University of Bristol, Bristol, United Kingdom; 6 Avon Longitudinal Study of Parents and Children (ALSPAC), School of Social and Community Medicine, University of Bristol, Bristol, United Kingdom; 7 Center for Bone and Arthritis Research, Institute of Medicine, Sahlgrenska Academy, University of Gothenburg, Gothenburg, Sweden; 8 Lexicon Pharmaceuticals, The Woodlands, Texas, United States of America; 9 Department of Medical Biochemistry, Oslo University Hospital, Ullevaal, Oslo, Norway; 10 Department of Pediatrics, Erasmus University Medical Center, Rotterdam, The Netherlands; 11 Department of Medicine, Human Genetics, McGill University, Montreal, Quebec, Canada; 12 Department of Epidemiology and Biostatistics, Lady Davis Institute for Medical Research, Jewish General Hospital, Montreal, Quebec, Canada; 13 Twin Research and Genetic Epidemiology, King's College London, London, United Kingdom; 14 Department of Medical Biochemistry, Oslo Deacon Hospital, Oslo, Norway; 15 School of Clinical Sciences, University of Bristol, Bristol, United Kingdom; Georgia Institute of Technology, United States of America

## Abstract

To identify genetic loci influencing bone accrual, we performed a genome-wide association scan for total-body bone mineral density (TB-BMD) variation in 2,660 children of different ethnicities. We discovered variants in 7q31.31 associated with BMD measurements, with the lowest P = 4.1×10^−11^ observed for rs917727 with minor allele frequency of 0.37. We sought replication for all SNPs located ±500 kb from rs917727 in 11,052 additional individuals from five independent studies including children and adults, together with *de novo* genotyping of rs3801387 (in perfect linkage disequilibrium (LD) with rs917727) in 1,014 mothers of children from the discovery cohort. The top signal mapping in the surroundings of *WNT16* was replicated across studies with a meta-analysis P = 2.6×10^−31^ and an effect size explaining between 0.6%–1.8% of TB-BMD variance. Conditional analyses on this signal revealed a secondary signal for total body BMD (P = 1.42×10^−10^) for rs4609139 and mapping to *C7orf58*. We also examined the genomic region for association with skull BMD to test if the associations were independent of skeletal loading. We identified two signals influencing skull BMD variation, including rs917727 (P = 1.9×10^−16^) and rs7801723 (P = 8.9×10^−28^), also mapping to *C7orf58 (*r^2^ = 0.50 with rs4609139). *Wnt16* knockout (KO) mice with reduced total body BMD and gene expression profiles in human bone biopsies support a role of *C7orf58* and *WNT16* on the BMD phenotypes observed at the human population level. In summary, we detected two independent signals influencing total body and skull BMD variation in children and adults, thus demonstrating the presence of allelic heterogeneity at the *WNT16* locus. One of the skull BMD signals mapping to *C7orf58* is mostly driven by children, suggesting temporal determination on peak bone mass acquisition. Our life-course approach postulates that these genetic effects influencing peak bone mass accrual may impact the risk of osteoporosis later in life.

## Author Summary

Genetic investigations on bone mineral density (BMD) variation in children allow the identification of factors determining peak bone mass and their influence on developing osteoporosis later in life. We ran a genome-wide association study (GWAS) for total body BMD based on 2,660 children of different ethnic backgrounds, followed by replication in an additional 12,066 individuals comprising children, young adults, and elderly populations. Our GWAS meta-analysis identified two independent signals in the 7q31.31 locus, arising from SNPs in the vicinity of *WNT16*, *FAM3C*, and *C7orf58*. These variants were also associated with skull BMD, a skeletal trait with much less environmental influence for which one of the signals displayed age-specific effects. Integration of functional studies in a *Wnt16* knockout mouse model and gene expression profiles in human bone tissue provided additional evidence that *WNT16* and *C7orf58* underlie the described associations. All together our findings demonstrate the relevance of these factors for bone biology, the attainment of peak bone mass, and their likely impact on bone fragility later in life.

## Introduction

Roughly 30 to 50% of women and 15 to 30% of men experience an osteoporosis-related fracture during their lifetime [1]. In adults, bone mineral density (BMD) measured at skeletal sites where osteoporotic fractures occur more frequently (i.e., lumbar spine, hip and forearm) is used for the diagnosis of osteoporosis and assessment of fracture risk. BMD measured at a given point in time is the result of peak-bone mass acquisition and subsequent bone loss in later life.

Due to the rapid changes in bone area in early life, the total body measurement (less head) is the preferred measurement to evaluate bone health in children [2]. The total body BMD measurement (in both children and adults) incorporates components of both cortical (∼80%) and to a lesser extent trabecular (∼20%) bone [3]. Moreover, it is likely that the genes underlying skeletal growth and bone loss differ in importance across the lifespan and can act in a site specific manner [4]–[6]. Peak bone mass is an important determinant of the risk of osteoporosis later in life [7], [8]. Early identification of individuals prone to low peak BMD may allow implementing strategies (interventions) which can delay the onset of osteoporosis.

From a genetic perspective, the discovery of loci influencing peak bone mass should be based on younger populations to avoid the noise introduced by bone loss later in life. A relatively recent Genome Wide Association Study (GWAS) in native British children successfully identified an association between total body derived BMD and variants in the osteoblast transcription factor gene *Osterix* [9], an early acting developmental gene shown to influence peak bone mass accrual but also BMD in adults [10], [11] .

The purpose of this study was to identify genetic variants associated with total body BMD (TB-BMD) in children, thus targeting variants involved in bone accrual. We ran a GWAS on children from the multiethnic Generation R Study and then replicated our findings in five additional cohorts including Northern European individuals covering different age groups ranging from children to elderly adults, allowing any life-course effect of the discovered variants to be evaluated.

## Results

### Association with total-body BMD in the discovery cohort

**Figure 1:**
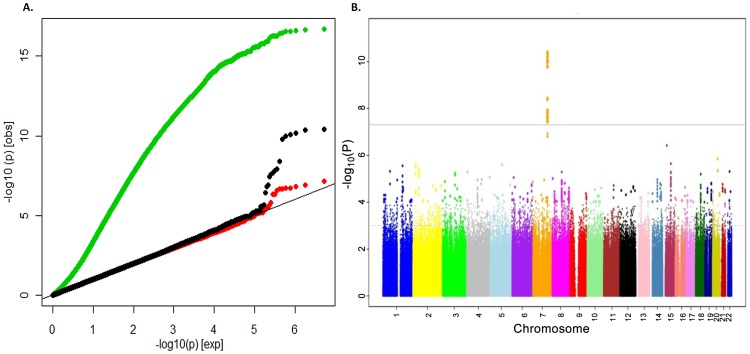
Genome-wide association of TB-BMD in the discovery cohort. A. Q-Q plot showing the inflation of the test statistics when correction for data structure is not applied (green dots) and the loss of power when no weight correction is applied (red dots) in comparison with the applied model (black dots) and B. Manhattan Plot of the genome wide association analysis of TB-BMD in the Generation R (discovery) cohort of model correcting by age, gender and body weight.

To search for loci influencing total-body BMD variation we performed genome-wide association analysis in a subset of 2,660 children from the Generation R Study with DXA scans and GWAS data. The Generation R Study is a population-based multiethnic birth cohort currently assessing children at an average age of 6.1 (SD 0.28) years. Table S1 shows population characteristics of these children overall and stratified by ethnicity. To increase the genome coverage of common variants we imputed genotypes for 3,021,329 SNPs in reference to the combined CEU, CHB/JPT and YRI HapMap Phase II panels using MACH/minimac software taking into account the admixed nature of the Generation R population [12]. The GWAS for TB-BMD in these individuals adjusted for age, gender, weight and 20 principal components, showed appropriate control for population structure with genomic inflation factors (λ) approaching unity (Figure 1A), and revealed a genome-wide significant association (lowest P = 4.1×10^−11^ for rs917727) mapping to the 7q.31 locus (Figure 1B).

### Replication of the 7q31.31 association signal

**Figure 2:**
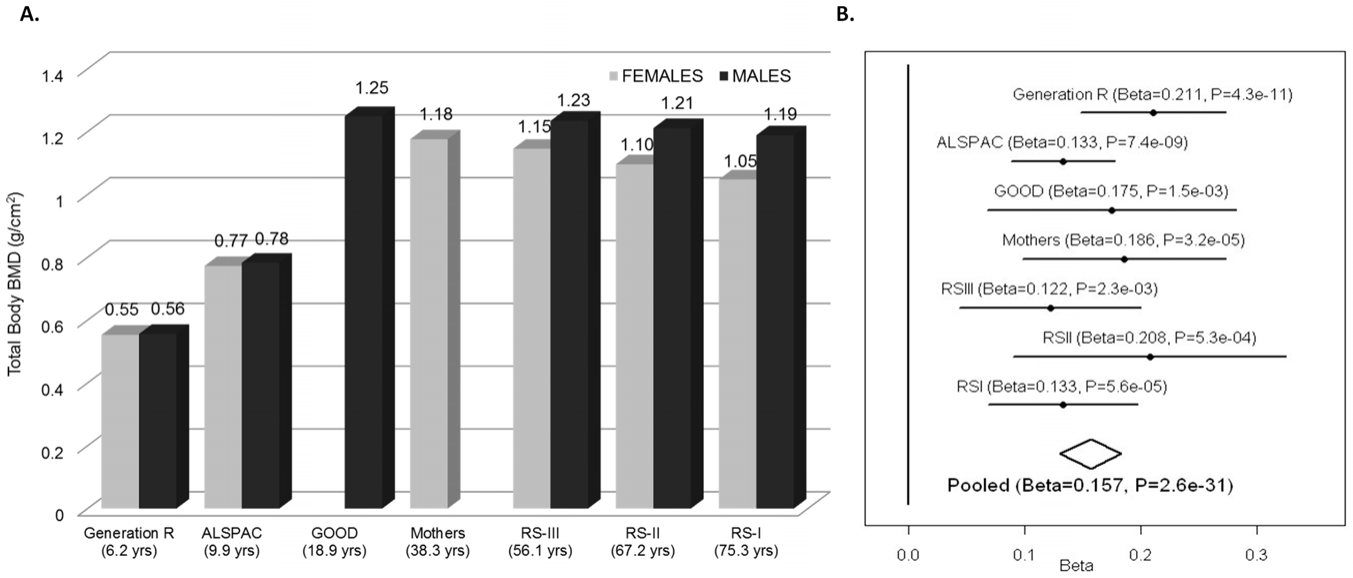
TB-BMD across cohorts and meta-analysis. A. Mean Total Body BMD in each cohort by gender showing the highest BMD levels in young adults and overall higher levels in male than in female participants. B. Forest plot of the association of TB-BMD and rs917727. Results are reported per copy of the G-allele (MAF = 0.27).

**Figure 3:**
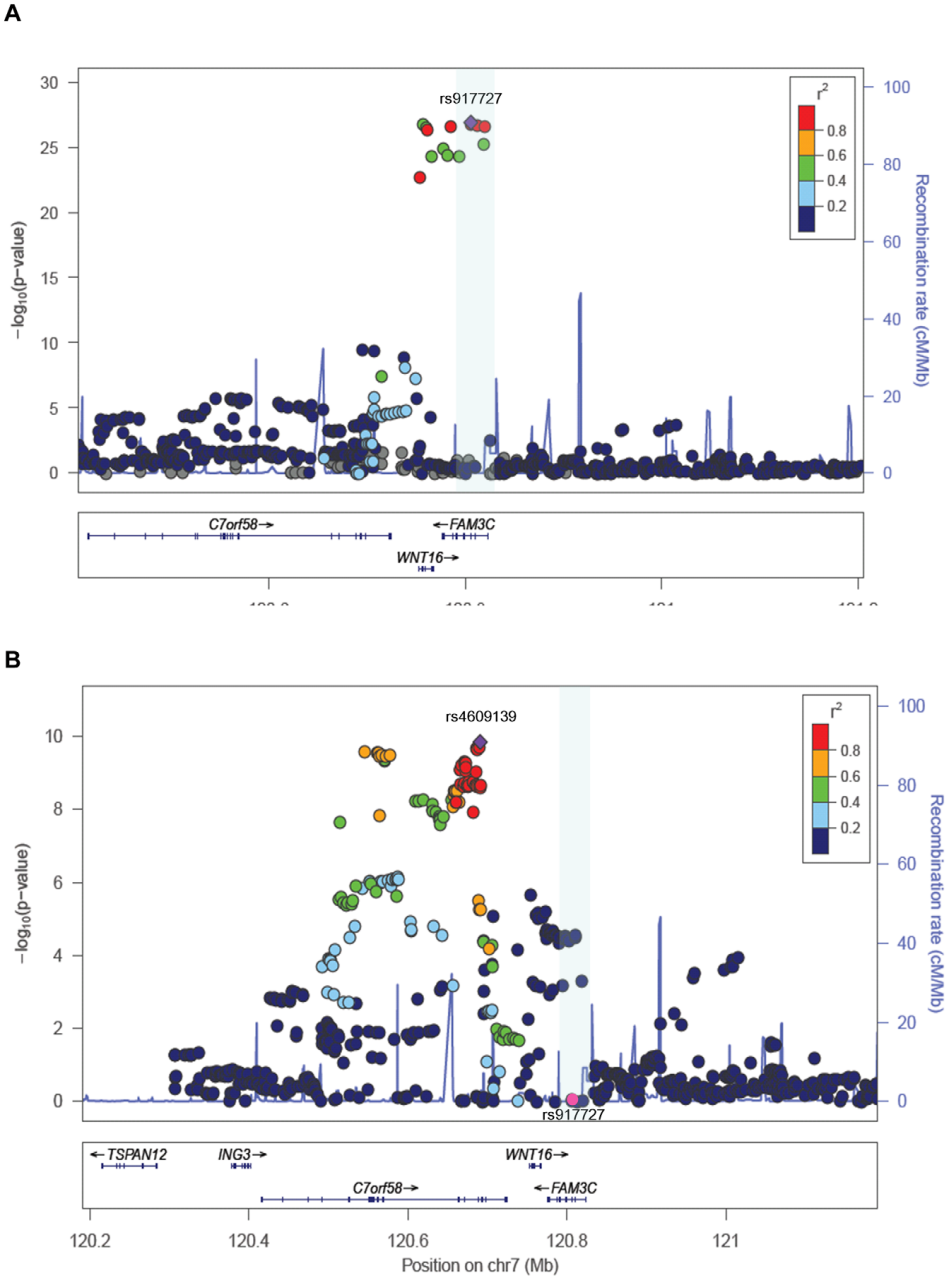
Association plots for TB-BMD. A. SNP association plot for TB-BMD-associated region of Chromosome 7q31.31. B. SNP association plot for TB-BMD-associated region of Chromosome 7q31.31 after conditioning on rs3801382. Genetic coordinates are as per Hapmap phase II-CEU. *Data from the mothers of Generation R is not included.

We sought replication of 721 SNPs spanning the region comprised by +/−500 kb from the top associated SNP (rs917727). We did this across five populations with total body DXA scans and GWAS data including: the *Avon Longitudinal Study of Parents and Children* (ALSPAC), the *Gothenburg Osteoporosis and Obesity Determinants* (GOOD) and the Rotterdam Study (RS-I, RS-II and RS-III) cohorts. These replication cohorts were selected to cover a wide spectrum of age groups to assess the genetic association with total body BMD variation throughout different life periods (Figure 2A) comprising: ALSPAC children (n = 5,334; mean age 9.9 years), GOOD young adults (n = 938; mean age 18.9 years) and a set of individuals over age 45 years RS-III (n = 1,594; mean age 56.1 years), RS-II (n = 750; mean 67.2 years); and RS-I, (n = 2,436, mean age 75.3 years). In addition, a sample of young women of Northern European descent (mothers of the Generation R participants) lacking GWAS scans, MoGENR, (n = 1,014; mean age 38 years) were *de-novo* genotyped for rs3801387, a perfect proxy (r^2^ = 1, based on the Hapmap phase II-CEU panel) of the top associated SNP. Detailed population characteristics of these cohorts can be found in Table S2. From the 721 SNPs used in the meta-analysis 20 surpassed the genome-wide significant threshold (Table 1) whilst a further 22 were suggestive of association (P<1×10^−5^). These SNPs had a minor allele frequency (MAF) ranging between 0.23–0.30 across studies (Table 1). The top associated SNP was rs917727 (P = 1.28×10^−27^ and P = 2.6×10^−31^ when including Generation R mothers), which had a combined effect of 0.16 SD increment per copy of the minor allele (Figure 2B). The effect of the rs917727 explained on average 0.9% of the phenotypic variance in standardized BMD and had no significant evidence for statistical heterogeneity across the meta-analyzed cohorts (I^2^ = 17%, P = 0.306). The GWAS signal mapped to a 66.3 Kb region of high LD (r^2^>0.8) between the *FAM3C* and *WNT16* genes (Figure 3A).

**Table 1:** Genome-wide significant markers of the total body BMD GWAS meta-analysis.

			DISCOVERY				REPLICATION														COMBINED			
VARIANT			Generation R			ALSPAC			GOOD			RS-III			RS-II			RS-I						
			n = 2,660			n = 5,334			n = 938			n = 1,594			n = 750			n = 2,436			n = 13,712***			
SNP	A1	R2*	Freq.	BETA**	P	Freq	BETA**	P	Freq	BETA**	P	Freq	BETA**	P	Freq	BETA**	P	Freq	BETA**	P	BETA**	P	I2	HetP
**rs917727**	T	1	0.296	0.21	**4.31E-11**	0.273	0.133	**7.43E-09**	0.238	0.175	0.002	0.274	0.122	0.002	0.263	0.208	5.34E-04	0.269	0.133	5.6E-05	0.154	**1.28E-27**	17	0.306
rs2908004	A	0.55	0.501	0.16	**1.23E-08**	0.442	0.122	**1.32E-09**	0.431	0.14	0.004	0.456	0.111	0.002	0.437	0.233	1.01E-05	0.449	0.124	5.01E-05	0.136	**1.66E-27**	0	0.416
rs917726	T	1	0.282	0.208	**6.42E-11**	0.273	0.134	**3.53E-09**	0.238	0.175	0.002	0.274	0.122	0.003	0.263	0.208	5.35E-04	0.269	0.133	6.39E-05	0.154	**1.80E-27**	11	0.347
rs718766	C	1	0.272	0.208	**8.63E-11**	0.273	0.134	**3.55E-09**	0.238	0.177	0.002	0.274	0.122	0.003	0.263	0.209	5.40E-04	0.269	0.134	6.60E-05	0.154	**2.17E-27**	11	0.347
rs3801382	G	1	0.275	0.199	**1.04E-10**	0.273	0.129	**3.37E-09**	0.238	0.171	0.002	0.274	0.12	0.003	0.263	0.206	5.22E-04	0.269	0.132	6.28E-05	0.149	**2.24E-27**	6	0.378
*rs7776725*	*C*	*1*	*0.264*	*0.214*	***4.58E-11***	*0.273*	*0.136*	***3.65E-09***	*0.239*	*0.187*	*0.001*	*0.274*	*0.123*	*0.003*	*0.263*	*0.209*	*5.49E-04*	*0.269*	*0.134*	*6.71E-05*	*0.156*	***2.36E-27***	*16*	*0.312*
*rs2536189*	*G*	*0.55*	*0.498*	*0.153*	***2.23E-08***	*0.442*	*0.122*	***1.32E-09***	*0.431*	*0.14*	*0.004*	*0.456*	*0.111*	*0.002*	*0.437*	*0.232*	*1.02E-05*	*0.449*	*0.124*	*5.04E-05*	*0.135*	***3.06E-27***	*0*	*0.444*
rs3801387	G	1	0.274	0.197	**1.61E-10**	0.272	0.129	**3.34E-09**	0.238	0.166	0.002	0.275	0.121	0.002	0.263	0.205	5.71E-04	0.270	0.131	7.09E-05	0.148	**4.32E-27**	0	0.415
rs4727924	T	0.51	0.467	0.172	**3.88E-09**	0.459	0.118	**7.05E-09**	0.454	0.144	0.003	0.477	0.112	0.002	0.463	0.218	2.57E-05	0.476	0.103	4.65E-04	0.132	**5.25E-26**	22	0.267
rs2536182	G	0.53	0.471	0.156	**2.69E-08**	0.454	0.116	**3.05E-09**	0.439	0.151	0.002	0.466	0.117	0.001	0.452	0.223	1.43E-05	0.463	0.098	8.54E-04	0.129	**1.28E-25**	20	0.286
rs2536180	C	0.51	0.495	0.143	1.57E-07	0.463	0.113	**5.66E-09**	0.456	0.137	0.003	0.478	0.11	0.002	0.465	0.215	2.30E-05	0.477	0.102	4.57E-04	0.124	**4.21E-25**	0	0.438
*rs2707466*	*T*	*0.51*	*0.485*	*0.152*	***3.55E-08***	*0.425*	*0.118*	***1.11E-08***	*0.418*	*0.142*	*0.004*	*0.440*	*0.111*	*0.003*	*0.424*	*0.237*	*1.03E-05*	*0.435*	*0.119*	*1.33E-04*	*0.133*	***5.13E-25***	*5*	*0.386*
rs2254595	C	0.51	0.504	0.142	1.20E-07	0.463	0.113	**5.92E-09**	0.456	0.137	0.003	0.478	0.11	0.002	0.464	0.214	2.45E-05	0.477	0.102	4.48E-04	0.124	**5.24E-25**	0	0.453
rs3779381	G	0.87	0.264	0.18	**1.57E-08**	0.255	0.123	9.95E-08	0.227	0.155	0.006	0.254	0.123	0.003	0.241	0.209	8.52E-04	0.250	0.137	7.81E-05	0.143	**2.00E-23**	0	0.623
rs2536150	C	0.07	0.215	−0.136	6.22E-05	0.176	−0.096	1.57E-04	0.168	−0.13	0.037	0.180	−0.011	0.812	0.174	−0.206	0.003	0.182	−0.071	0.059	−0.099	**3.17E-10**	37	0.162
rs2952559	C	0.06	0.265	−0.133	6.34E-05	0.183	−0.105	4.24E-05	0.165	−0.114	0.077	0.180	−0.034	0.475	0.184	−0.198	0.005	0.184	−0.050	0.180	−0.099	**3.94E-10**	26	0.240
rs13247600	C	0.04	0.064	−0.219	7.07E-04	0.076	−0.117	7.09E-03	0.061	−0.18	0.061	0.082	−0.085	0.244	0.084	−0.266	0.022	0.079	−0.219	3.17E-04	−0.163	**1.31E-09**	0	0.462
rs2707520	C	0.25	0.444	−0.091	0.001	0.502	−0.06	0.003	0.544	−0.04	0.416	0.475	−0.066	0.070	0.532	−0.098	0.065	0.517	−0.090	0.003	−0.073	**6.90E-09**	0	0.865
rs17509082	T	0.39	0.173	0.119	0.001	0.198	0.065	0.007	0.182	0.041	0.492	0.202	0.067	0.133	0.187	0.170	0.008	0.201	0.090	0.010	0.084	**3.21E-08**	0	0.536
rs2908007	A	0.37	0.492	−0.1	4.12E-04	0.603	−0.046	0.027	0.628	−0.062	0.216	0.613	−0.067	0.070	0.610	−0.030	0.569	0.608	−0.100	0.001	−0.070	**4.78E-08**	0	0.528

Ty = Type of SNP Imputed (I) Genotyped (G).Bolded rs917727 top-hit, rs3801382 used for conditioning and rs3801387 genotyped in Generation R Mothers.

*: Correlation coefficients with rs917727 based on HapMap release22 CEU population.

**: Effect estimates expressed as standardized adjusted SD per copy of allele (A1).

***: Results do not include Generation R mothers only genotyped for rs3801387. Underline: rs7776725 Top-hit wrist fracture GWAS, rs2536189 Top-hit forearm BMD GWAS, rs2707466 Top-hit for Cortical thickness in Zheng et al (accompanying submission).

### Conditional analyses for secondary signals

To assess the presence of allelic heterogeneity at the locus we carried out a conditional analysis conditioning on the top signal. After meta-analysis we identified a secondary independent signal mapping to *C7orf58* (Figure 3B) including 67 SNPs surpassing (P<5×10^−8^) genome-wide significant level (Table S3). In general, most of these 67 SNPs were in high LD with each other (r^2^: 0.80–1.00, based on the Hapmap phase II-CEU panel), displayed moderate heterogeneity across cohorts (I^2^ between 11 and 47%) and had smaller effect sizes (standardized SD −0.067 to −0.081) as compared to those observed for the main signal. The lead SNP rs4609139 associated at P = 1.42×10^−10^ (Figure S1) had an average MAF of 0.35 across studies and a combined effect of −0.08 BMD standard deviations (SE:0.0126) per copy of the minor allele, explaining on average 0.2% of the phenotypic variance in standardized BMD.

### Evaluation of covariates

In the discovery cohort we observed prominent effects of the covariates on the SNP-phenotype relationships. To illustrate this we present beta estimates as standardized coefficients from null-intercept centered models in Table S4 (T-S4). As expected, lack of correction for principal components of the sex- and age- adjusted model (Model 0 in T-S4 and Figure 1A) generated important inflation of the test statistic, which is severely reduced by inclusion of twenty principal components (Model 1 in T-S4 and Figure 1A). Inclusion of weight in the sex-, age- and PCs- corrected model (Final Model in T-S4 and Figure 1A) increased the significance of the putative signal by reducing the standard error of the SNP effect estimate (from 0.030 to 0.022). Such reduction of the error variance led to genome-wide significance after weight correction. Weight is an important determinant of peak bone mass accrual related to both loading and size effects as illustrated by the positive relationship with total body BMD also evident across our SNP-Phenotype models (Table S4).

### Association with skull BMD

**Figure 4:**
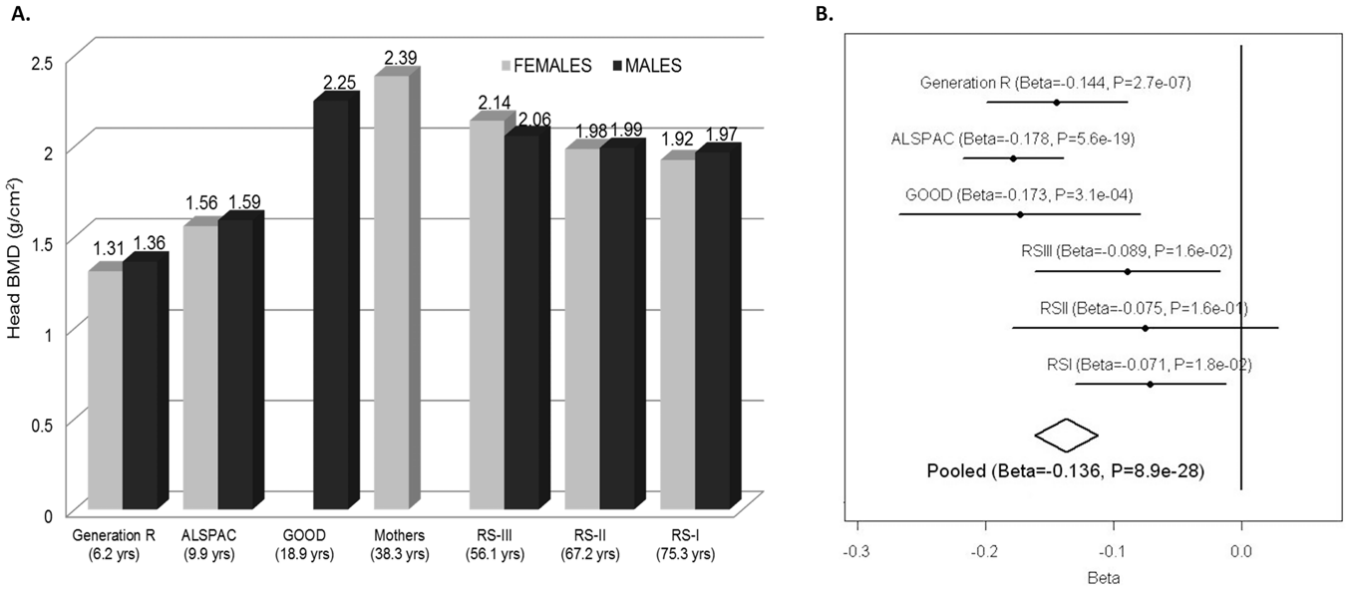
Skull-BMD across cohorts and meta-analysis. A. Gender-specific mean Skull BMD for each cohort. B. Forrest plot of the association of skull BMD with rs7801723. The results are reported per copy of the T-allele (MAF = 0.37).

**Figure 5:**
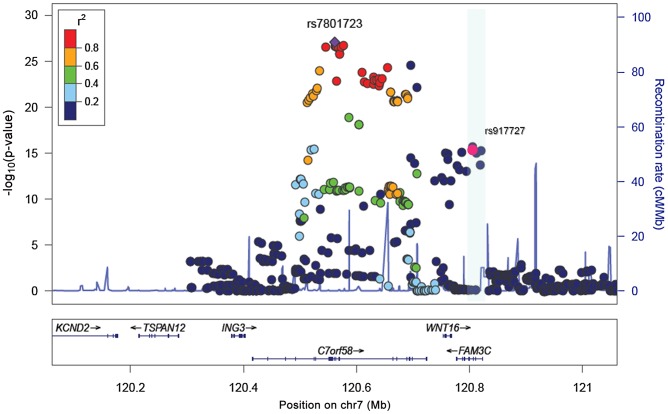
Association plots for skull-BMD. SNP association plot for the skull BMD-associated region in chromosome 7q31.31, based on 13,712 individuals from the five different cohorts with GWAS information.

The impact of weight correction on the standard errors of the association led us to hypothesize that, at least in children, the effect of variants in the *WNT16* region on total-body BMD was independent of skeletal loading (of which body weight is a proxy). For this reason, we analyzed the 721 SNPs of the same genomic region in relation to skull BMD across all five cohorts with GWAS data and total body BMD (without Generation R mothers). Skull BMD measured by the total body DXA scan constitutes an independent measurement in children since the head region is excluded from the total body BMD assessment. This is done given the large variation in density and area inflicted by the skull on the head region, which is particularly evident in paediatric populations [13]. The head DXA region is suitable to evaluate the relationships with skeletal loading considering that its direct influence on the mineralization process of the skull is negligible. Despite being a skeletal region composed of laminar bones, the proportion of mostly cortical (95% for the inner and outer layers) but also trabecular (inner lamella) bone in the skull is similarly high to that of the overall skeleton (80% cortical) as compared to other skeletal sites. In addition, the BMD of the skull is subject to the same patterns of peak bone mass accrual and decrease with aging observed across the lifetime (Figure 4A). Since, as expected, weight was not a significant covariate in the analysis of skull BMD (P = 0.09) we performed the meta-analysis using a sex- age-, height- and PCs-corrected model across the five cohorts with GWAS data. The strongest association signal with skull BMD mapped to *C7orf58* (the gene underlying the secondary signal of TB-BMD). The most significantly associated SNP was rs7801723 with MAF between 0.33 and 0.39 across cohorts and in moderate LD (r^2^ = 0.56) with rs4609139 (secondary signal in TB-BMD). The combined effect of rs7801723 was −0.14 BMD standard deviations (SE:0.012) per copy of the minor allele (P = 8.9×10^−28^) showing a high heterogeneity I^2^ of 60.7% and P_het_ = 0.03 (Figure 4B). Moreover, we identified 147 variants in the 7q31.31 locus achieving genome-wide significance (Table S5) and suggesting the existence of two independent signals (Figure 5) in the regional meta-analysis of skull BMD. The rs917727 SNP (primary signal of total body BMD) was also associated at genome-wide significant level (P = 1.9×10^−16^) with skull BMD with an effect estimate of 0.12 BMD standard deviations (SE:0.014) per copy of the minor allele and no evidence for significant heterogeneity (I^2^ = 0%).

The heterogeneity at rs7801723 appeared to be driven by different effects in younger and older populations (Figure 4B). For this reason, we stratified the analysis according to whether individuals within the cohorts had achieved total skeletal maturation (RS-I, RS-II, RS-III) or were still in the process of peak bone accrual (GOOD, ALSPAC, GEN-R). The rs7801723 signal seemed to be strongest in the younger populations (B = −0.16; P = 2.06×10^−27^) since the effect was considerably weaker in older populations (B = −0.08; P = 2.7×10^−4^), though differences in power due to lower sample size may also play a role (Figure S2). Meta-regression across studies showed a significant relation between mean age and absolute effect sizes observed for rs7801723 (Figure S3) on skull BMD (Beta_age_ meta-regression = 0.0015; P = 0.006) but not on total BMD (Beta_age_ meta-regression = 0.0008; P = 0.12). In contrast, the effect of rs917727 on skull BMD seems to be uniform across older (B = 0.14; P = 4.16×10^−9^) and younger (B = 0.10; P = 4.28×10^−9^) populations, displaying no evidence of effect heterogeneity nor a significant relation with the mean age across studies (Beta_age_ meta-regression = −0.0006; P = 0.19). Similarly, the effect size of rs917727 on total body BMD was not related to the mean age of the studies (Beta_age_ meta-regression = −0.0004; P = 0.57).

### Functional evaluation of the 7q31.31 locus

The effect of variants from the 7q31.31 locus on both total body and skull BMD cannot be unequivocally attributed to any of the closest three genes in the GWAS signal region (*WNT16*, *FAM3C* and *C7orf58*). This is also complicated by the high LD across the region. Based on current knowledge, *WNT16* is the best candidate at the locus considering that it belongs to the Wnt family of proteins. The Wnt signaling pathway plays ubiquitous key roles in fundamental biological processes, including those critical for bone biology and specifically for bone formation [14], [15]. *FAM3C* is a widely expressed gene (including in osteoblasts) which belongs to a cytokine-like gene family without homology to any known cytokines [16]. Minimal information exits about the functional aspects of *C7orf58*. *C*onsidering the hypothesis-free nature of our GWAS approach we cannot exclude the possibility that any of these genes may code for proteins involved in BMD regulation.

### *Wnt16* and *Fam3c* KO mouse models

Further evidence implicating *WNT16* as the gene underlying these associations with total body BMD at the population level is provided by functional studies on *Wnt16* knockout (KO) mice generated at Lexicon Pharmaceuticals (Table 2). These KO mice have reduced total body BMD at 24 weeks of age, resulting from both reduced total body bone mineral content (BMC) and bone area. BMC and aBMD measured at the spine (a skeletal site more strongly influenced by trabecular bone than total body or femur measurements) were slightly reduced in KO mice but this reduction did not achieve statistical significance. Male and female knockout mice appeared healthy with no discernible morphological or growth defects, and normal femur length, body weight and body composition. We also examined mice from three *Fam3c* KO models testing for differences across DXA phenotypes with wild type animals. We failed to observe any significant differences across the skeletal phenotypes in each independent *Fam3c* KO strategy (Table S6). Even though these data suggest *Fam3c* does not influence bone mass in mice, the possibility of a false negative due to power limitations cannot be excluded.

**Table 2:** Summary statistics for densitometric properties of control (+/+) and *Wnt16* deficient (−/−) mice.

Parameter	Male WT Mice	Male *Wnt16* KO Mice	Statistics	Female WT Mice	Female *Wnt16* KO Mice	Statistics
Number of mice	9	12		24	16	
Body Weight (grams)	36.4±1.8	38.5±1.2	Δ = ↑5%, P = 0.42	28.2±0.8	26.8±1.2	Δ = ↓5%, P = 0.30
Lean Body Mass (grams)	27.0±0.9	27.9±0.7	Δ = ↑4%, P = 0.42	20.5±0.4	19.6±0.6	Δ = ↓4%, P = 0.20
Body Fat (percent)	23.1±2.1	24.9±1.4	Δ = ↑8%, P = 0.48	24.2±1.2	23.2±1.6	Δ = ↓4%, P = 0.63
Femur Length (mm)	16.3±0.2	16.2±0.2	Δ = ↑1%, P = 0.58	16.2±0.1	16.1±0.1	Δ = 0%, P = 0.86
Body aBMD (mg/cm2)	56.9±1.1	54.8±0.7	Δ = ↓4%, P = 0.11	53.9±0.6	48.7±0.6	**Δ = ↓10%, P<0.001**
Body Bone Area (cm2)	9.4±0.2	8.6±0.3	**Δ = ↓9%, P = 0.03**	8.9±0.2	8.1±0.1	**Δ = ↓8%, P = 0.002**
Body BMC (mg)	532±16	470±18	**Δ = ↓12%, P = 0.02**	479±11	396±10	**Δ = ↓17%, P<0.001**
Femur aBMD (mg/cm2)	89.4±3.3	83.6±1.5	Δ = ↓7%, P = 0.10	78.5±1.3	62.8±1.2	**Δ = ↓20%, P<0.001**
Femur Bone Area (cm2)	0.36±0.01	0.34±0.01	Δ = ↓7%, P = 0.13	0.35±0.01	0.31±0.01	**Δ = ↓13%, P<0.001**
Femur BMC (mg)	32.6±1.8	28.0±1.0	**Δ = ↓17%, P = 0.03**	27.8±0.6	19.5±0.7	**Δ = ↓30%, P<0.001**
Spine aBMD (mg/cm2)	61.9±1.5	58.8±3.7	Δ = ↓5%, P = 0.50	62.8±1.8	58.2±1.6	Δ = ↓7%, P = 0.08
Spine BMC (mg)	25.8±0.9	23.2±2.0	Δ = ↓10%, P = 0.29	26.3±0.9	24.3±0.9	Δ = ↓8%, P = 0.16

Results provided as [mean +/− SEM].

### Gene transcript–phenotype correlations

We examined the correlation of gene expression transcript levels derived from iliac bone crest biopsies in relation to BMD levels in a distinct cohort of 78 unrelated-Norwegian women with total body scans (of which 51% have osteoporosis) and who are part of a set (n = 84) described in detail previously [17]. The investigated region comprised +/−500 Kb of rs917727 and contained seven different genes including *TSPAN12* (3 transcripts), *ING3* (4 transcripts), *C7orf58* (2 transcripts), *WNT16* (2 transcripts), *FAM3C* (3 transcripts), *PTPRZ1* (1 transcript) and one represented by the Affymetrix probe with ID *217206_at* lacking annotation. We only identified significant correlations with BMD measurements of the donors in transcripts from *C7orf58* and W*NT16* (Table 3). Expression levels in one of the transcripts in *WNT16* (224022_x_at) was significantly associated with BMD measured at several skeletal sites including the total body, skull, legs, total hip and lumbar spine (L1–L4 vertebrae). The correlation was positive and of similar magnitude across sites, ranging between 0.25 and 0.31, indicating that higher expression of this gene is correlated with higher BMD, results which are in line with the *Wnt16* KO mice data. Significant correlation with total body lean mass was also observed (r^2^ = 0.31) for this transcript. This suggests a pleiotropic effect on muscle considering that correction for BMI did not importantly influence the correlation of the transcript with total body BMD. There is even a stronger (inverse) correlation of total body and skull BMD with expression levels of one of the *C7orf58* transcripts (228728_at), even approaching a correlation of −0.50 with total body BMD. This suggests high *C7orf58* expression levels of this transcript are related to lower BMD. This transcript was inversely correlated with body weight as well, but still maintained a strong (inverse) correlation (r^2^ = −0.45) with skull BMD (which as discussed above, is not readily affected by body weight) and also with BMI-adjusted total body BMD (r^2^ = −0.42). In addition, levels of both *C7orf58* transcripts were significantly correlated with age, which further supports the age-specific effects seen for the GWAS variants mapping to *C7orf58*.

**Table 3:** Expression analysis of *C7orf58, WNT16*, and *FAM3C* transcripts in hip bone biopsies from 78 Norwegian women.

Gene symbol		*C7orf58*		*C7orf58*		*WNT16*		*WNT16*		*FAM3C*		*FAM3C*		*FAM3C*	
Affymetrix transcript ID		220032_at		228728_at		221113_s_at		224022_x_at		240062_at		236316_at		201889_at	
Exons covered by probeset		16–19		24		4–5		5		1a		6–7		10	
	Mean (SD)	Pearson r	P	Pearson r	P	Pearson r	P	Pearson r	P	Pearson r	P	Pearson r	P	Pearson r	P
**Age (years)**	64.2 (9.6)	**0.24**	**0.032**	**0.24**	**0.031**	0.04	0.720	−0.05	0.680	0.14	0.230	0.04	0.710	0.16	0.170
**Weight (kg)**	66.0 (12.0)	**−0.26**	**0.022**	0.02	0.860	−0.13	0.250	0.19	0.089	−0.05	0.640	−0.07	0.530	−0.05	0.650
**BMI (kg/cm2)**	23.9 (3.5)	**−0.26**	**0.021**	0.04	0.760	−0.16	0.170	0.13	0.280	−0.02	0.880	0.00	0.980	−0.03	0.760
**Height (cm)**	165.9 (7.6)	−0.12	0.320	−0.04	0.740	−0.01	0.940	0.18	0.110	−0.09	0.450	−0.14	0.220	−0.08	0.460
**Total body BMD (g/cm^2^)**	1.06 (0.15)	**−0.49**	**5.00E-06**	−0.16	0.150	−0.20	0.085	**0.29**	**0.011**	−0.03	0.810	−0.02	0.860	−0.20	0.083
**Total body T-score**	−0.81 (1.83)	**−0.49**	**5.20E-06**	−0.16	0.150	−0.19	0.090	**0.29**	**0.010**	−0.03	0.780	−0.02	0.850	−0.19	0.091
**Total body Z-score**	0.19 (1.52)	**−0.44**	**6.20E-05**	−0.13	0.270	−0.17	0.150	**0.26**	**0.020**	0.02	0.870	0.00	0.990	−0.17	0.140
**Total body Z-score, BMI adj**	−0.19 (1.51)	**−0.42**	**1.20E-04**	−0.13	0.250	−0.16	0.170	**0.26**	**0.024**	0.02	0.870	0.00	1.000	−0.17	0.150
**Head BMD (g/cm^2^)**	2.07 (0.42)	**−0.46**	**2.90E-05**	−0.09	0.440	**−0.24**	**0.037**	**0.28**	**0.012**	−0.07	0.560	−0.11	0.320	−0.21	0.071
**Arms BMD (g/cm^2^)**	0.80 (0.12)	**−0.37**	**7.90E-04**	−0.18	0.110	−0.21	0.070	0.19	0.089	−0.06	0.630	0.02	0.880	−0.17	0.140
**Legs BMD (g/cm^2^)**	1.13 (0.18)	**−0.47**	**1.70E-05**	−0.19	0.087	−0.14	0.210	**0.27**	**0.016**	−0.03	0.820	0.01	0.910	−0.17	0.140
**Total Hip BMD (g/cm^2^)**	0.88 (0.19)	**−0.42**	**1.90E-04**	−0.18	0.110	−0.16	0.160	**0.29**	**0.012**	−0.01	0.940	0.06	0.630	−0.11	0.350
**Total Hip T-score**	−0.99 (1.61)	**−0.43**	**9.70E-05**	−0.18	0.120	−0.14	0.210	**0.28**	**0.014**	0.00	0.990	0.07	0.550	−0.12	0.310
**Total Hip Z-score**	0.03 (1.39)	**−0.36**	**1.10E-03**	−0.13	0.270	−0.12	0.300	**0.25**	**0.025**	0.05	0.660	0.10	0.370	−0.08	0.490
**Total Hip Z-score, BMI adj**	−0.19 (1.35)	**−0.31**	**5.50E-03**	−0.14	0.230	−0.09	0.460	**0.23**	**0.040**	0.06	0.630	0.11	0.360	−0.07	0.520
**L1–L4 BMD (g/cm^2^)**	0.97 (0.25)	**−0.45**	**3.20E-05**	−0.06	0.630	−0.22	0.054	**0.31**	**0.006**	0.00	0.970	−0.05	0.660	−0.21	0.068
**L1–L4 T-score**	−1.68 (2.01)	**−0.44**	**7.00E-05**	−0.05	0.680	−0.20	0.082	**0.30**	**0.007**	0.03	0.820	−0.03	0.770	−0.20	0.074
**L1–L4 Z-score**	−0.47 (1.81)	**−0.38**	**7.20E-04**	0.01	0.950	−0.18	0.110	**0.29**	**0.011**	0.06	0.600	−0.02	0.860	−0.18	0.110
**L1–L4 Z-score, BMI adj**	−0.29 (1.78)	**−0.34**	**2.60E-03**	0.00	0.990	−0.16	0.170	**0.27**	**0.017**	0.06	0.580	−0.02	0.850	−0.18	0.120
**Total body Lean mass (kg)**	40.4 (4.8)	−0.22	0.055	0.04	0.720	0.04	0.710	**0.31**	**0.005**	−0.03	0.780	−0.13	0.270	−0.02	0.830
**Total body Fat mass (kg)**	23.0 (8.4)	−0.22	0.058	0.00	0.970	−0.20	0.075	0.08	0.510	−0.07	0.560	−0.03	0.800	−0.07	0.560

## Discussion

In this study we carried out a genome-wide association scan of total body BMD in 2,660 children with regional replication of the top hit in 12,066 children and adults from six additional studies, including *de novo* genotyping of a sample of 1,014 Northwestern European pre-menopausal women, mothers of the children from the discovery cohort. We identified at least two independent signals (primary rs917727 and secondary rs4609139) associated with total body BMD mapping to the 7q31.31 locus harboring (among others) two genes and an open reading frame sequence including *WNT16, FAM3C* and *C7orf58*. To examine whether the observed associations were independent of skeletal loading we tested variants in the 7q31.31 region for association with skull BMD, a non-weight bearing skeletal site. We found the rs917727 influencing skull BMD and an even stronger signal arising from rs7801723 (in partial LD with rs4609139) which unlike the total body signals was largely driven by the younger (children) populations. A DXA-based assessment of the *Wnt16* KO mice showed a lower total body BMD phenotype which is compatible with an effect of *WNT16* variants playing a role on total body BMD at the human population level. Moreover, the analysis of gene transcript expression profiles supports the involvement of variants from both *WNT16* and *C7orf58*. Together, these findings postulate that the *WNT16/C7orf58* locus contains complex patterns of genetic variation, which play an important role in peak bone mass accrual and may likely impact BMD determination at later life.

Genetic variants in the region mapping to *FAM3C* as the closest gene, have been previously reported in the literature as associated with speed of sound as analyzed by quantitative ultrasound in radius and calcaneous in a Korean population [18] and in a recent follow-up study the same SNPs were genotyped *de novo* and were found to be associated with bone mineral density at different sites in individuals of European descent [19]. Nonetheless, to our knowledge this is the first time this locus is associated with total body BMD in children. Total body BMD measured in children corresponds only to the amount of bone accrued up to that point in time; consequently the variants described here have a definite role in the genetic determination of bone acquisition. Furthermore, considering the previously reported association in East Asians, the multiethnic background of our discovery population and the lack of heterogeneity in the total-body signal, one can conclude the effect of these variants is present across populations of different genetic background. To assess this in more detail we examined in the multiethnic Generation R discovery cohort the top associated SNPs of the meta-analysis across clusters of different ethnic backgrounds (Table S7). We confirmed that the effect of the top associated variants was not confined to individuals of European descent. Effect directions and magnitude of the markers were largely similar across groups, despite some evident differences in linkage disequilibrium patterns between markers.

Without additional functional evaluation it is not feasible to undisputedly distinguish which genes in the 7q31.31 region could be underlying the observed GWAS signals. The analysis of gene expression profiles provides supporting evidence for the involvement of variants from *WNT16* and *C7orf58*, while not for *FAM3C* in relation to total body BMD and skull BMD. Similarly, the lower likelihood of an effect arising from *FAM3C* is supported by the absence of an abnormal skeletal phenotype in the *Fam3c* KO mice, although we cannot exclude the possibility that variants resulting in gain of function of *Fam3c* affect bone. In contrast, functional evidence of the involvement of *WNT16* is well supported by the analysis of expression profiles and the observation of reduced BMD in the *Wnt16* KO mice. The *WNT16* human/mouse sequence alignment shows 93% identity favoring the plausibility of similarity in phenotypic effects across both species. Even though the *Wnt16* KO mice had reductions of both BMC (strongest) and bone area, in humans we found no indication of an effect on bone size (area) in either children or adults. This is consistent with the lack of any observed association between common genetic variation in this region and adult body height as assessed in a sufficiently powered GWAS [20]. We can infer a prominent effect of *WNT16* on cortical bone in humans considering the close resemblance of the cortical phenotype observed in the *Wnt16* KO mice to that reported in humans by Zheng et al. based on pQCT (accompanying submission). This prominent genetic effect on cortical bone is also manifested by the associations of *WNT16* variants with BMD traits measured at skeletal sites rich in cortical bone including the total body, the skull and the forearm as also shown by Zheng et al. (accompanying submission). In addition, Zheng et al. show that this effect on cortical bone influences bone strength in mice and fracture risk in humans. Nevertheless, the KO mouse data should be interpreted with caution since our functional validation sought the confirmation of the KO strategy but did not assess the integrity of the surrounding genomic region (i.e. intact *FAM3C* and/or *C7orf58* function). Further, total body BMD also involves components of trabecular bone which, together with the associations previously reported with ultrasound of the heel [18] and lumbar spine [19] (sites of rich trabecular content), do not fully exclude an effect of *WNT16* (or *FAM3C*) variants on this type of bone.

Both the *secondary signal* unveiled by our total body BMD GWAS and the *strongest signal* in the skull BMD GWAS analyses map to an open reading frame sequence (*C7orf58*) in the region. The effect size for rs4609139 on total body BMD (mapping to *C7orf58*) was up to 50% lower in magnitude than that of rs917727 (mapping in the vicinity of *WNT16*). In contrast, the associations with skull BMD were stronger for variants from the *C7orf58* signal than for those arising from *WNT16*. Further study of the mechanisms by which *C7orf58* exerts its role either independently or together with *WNT16* is warranted.

Several aspects derived from the analysis of skull BMD merit further discussion. Skull BMD is minimally influenced by loading, muscular activity, and in general less masked by environmental influences. From this perspective, fine-tuned mechanosensing mechanisms involved in the regulation of bone metabolism can be better dissected examining skull BMD. The skull BMD associations arising from the *C7orf58* signal are substantially more prominent in the younger populations. Such age dependency was not seen for the signals arising from *WNT16*, which also shows effects already evident at young age, but that do persist through adulthood until very old age, as corroborated by the replication studies in the older population cohorts. Also, from the strong associations with skull BMD we can infer that these effects likely to be arising from *WNT16* and *C7orf58* are not mediated by a mechanosensing response to skeletal loading. A recent study examining expression patterns at different skeletal sites in rats has shown differential gene expression patterns between the skull, the limbs and the total body, which likely reflects different responses to loading and mechanosensing between skeletal sites [21]. In the latter study, *WNT16* showed opposite expression patterns in arms than in skull. While in rats only one isoform is active, in humans different WNT16 isoforms could play distinct regulatory roles on skeletal development and/or metabolism [22]. Alternatively, modulation by other signaling factors could also modify the (up-/down-) streaming effects of *WNT16*. Recently, it has been shown that WNT16 influences hematopoietic stem cell differentiation via non-canonical Wnt signaling in zebra fish [23]. Whether this is also the case in bone biology remains to be confirmed, since its effect through canonical (beta-catenin mediated) activation has already been established in cartilage [24].

The allelic heterogeneity demonstrated by the conditional analysis on total body BMD is indicative of multiple (at least 2) causal variants in the region within and across phenotypes. The associations with TB-BMD observed for the top hits reported in the accompanying submission by Zheng et al. (namely rs2707466 for pQCT, rs2536189 for forearm BMD and rs7776725 for wrist fracture) are affected differently by conditional analysis on the top signal. As predicted by the complete linkage disequilibrium between rs917727 (top total body BMD SNP) and rs777625 (top wrist fracture SNP associated with TB-BMD with B = 0.156; P = 2.36×10^−27^), after conditioning, the effect of rs777625 on TB-BMD is largely gone (B = −0.0013; P = 0.93). In contrast, the effects on TB-BMD of rs2536189 (forearm BMD from B = 0.135 P = 3.06×10^−27^ to B = 0.042; P = 6.7×10^−4^) and rs2707466 (pQCT from B = 0.133 P = 5.13×10^−25^ to B = 0.043 P = 6.8×10^−4^) are not completely explained by their moderate LD (with r^2^ values between 0.51 and 0.55) with the top associated SNP of the TB-BMD GWAS signal.

In addition to the top associated pQCT SNP (rs2707466), there is yet another non-synonymous variant (rs2908004) annotated within the coding region of *WNT16* which is genome wide significant in our meta-analysis of total body BMD. After conditional analysis the effect sizes of these non-synonymous SNPs show considerable reduction suggesting there are not likely causal to the stronger top GWAS signal. Nevertheless, the residual effect is still significant after conditioning (indicating independence and) implying a weaker effect on TB-BMD or (more likely) partial linkage disequilibrium with yet other causal variants. Such relationships between genetic variants can follow diverse types of complex relationships as recently described for loci displaying allelic heterogeneity [25] for which follow-up investigations are warranted to elucidate the definitive involvement of the genes in this 7q31.31 region.

Finally, this region harbors one or more genes revealing critical effects on bone biology. While we have shown that genetic variants in this locus influence total body BMD variation in children of multiple ethnic background, the relevance of our findings are manifested by the persistence and consistency of the associations observed later in life. Further, the prominent effect on total body BMD we describe agrees with associations observed in adults across diverse skeletal traits [18], [19] (see also Zheng et al. accompanying submission), but most importantly, by their effect on risk of fracture (see also Zheng et al. accompanying submission), the most deleterious consequence of osteoporosis.

In summary, this study detected at least two independent GWAS signals influencing total body and skull BMD variation in children, thus confirming the presence of allelic heterogeneity in this *WNT16* locus. In addition, we showed how the effects observed in children are consistently replicated in adults. Specific genetic determination of peak bone mass (rather than bone loss later in life) is suggested by more prominent effects of some markers in children than in adults. These genetic effects likely influence the attainment of peak bone mass accrual and impact the risk of osteoporosis and fracture later in life.

## Methods

### Ethics statement

All research aims and the specific measurements in the participating studies involving human beings have been approved by the correspondent Medical Ethical Committee. Written informed consent was provided by all subjects or their parents in the case of children. Mouse studies were performed in accordance with institutional and regulatory guidelines for animal care and use at Lexicon Pharmaceuticals.

### Subjects

#### Generation R Study

The Generation R Study is a prospective cohort study in which 9,778 pregnant women living in Rotterdam and with delivery date from April 2002 until January 2006 were enrolled. Details of study design and data collection can be found elsewhere [26]. The current study comprised 2,660 children (mean age 6.16, SD = 0.39 years), of which 1,511 are of Dutch Northern European origin, who had both GWAS and DXA-based BMD measurements. DXA measurements were recorded on children visiting a unique research centre at around 5 years old accompanied by their mothers. All research aims and the specific measurements in the Generation R Study have been approved by the Medical Ethical Committee of the Erasmus Medical Center, Rotterdam and written informed consent was provided by all parents.

#### Avon Longitudinal Study of Parents and Children (ALSPAC)

In-silico replication of the GWAS signals was initially pursued in The Avon Longitudinal Study of Parents and Children (ALSPAC). This is a longitudinal population-based birth cohort that recruited pregnant women residing in Avon, UK, with an expected delivery date between 1^st^ April 1991 and 31^st^ December 1992. This cohort is described in detail on the website (http://www.alspac.bris.ac.uk) and elsewhere [27], [28]. Total body BMD and genome-wide SNP data were available for 5,334 unrelated children (mean age = 9.9, SD = 0.32 years) all of Northern-European descent. Ethical approval was obtained from the ALSPAC Law and Ethics committee and relevant local ethics committees, and written informed consent was provided by all parents.

#### The Gothenburg Osteoporosis and Obesity Determinants (GOOD)

The GOOD Study is a population-based cohort in which male subjects from between 18 and 20 years of age in the Gothenburg area in Sweden were randomly selected using national population registers and invited to participate in this initiative by phone. From the selected candidates 1,068 agreed to participate providing oral and written informed consent [29], [30]. The GOOD Study was approved by the local ethics committee at Gothenburg University. A subset of 938 individuals from this study with DXA measurements and GWAS data were included in this analysis.

#### Rotterdam Study (RS I, II, and III)

Additional in-silico replication in elderly adults was pursued in participants of the Rotterdam Study, a large prospective population-based cohort study of white subjects aged 45 years and older living in the Ommoord District of Rotterdam, The Netherlands who are studied for the occurrence of chronic diseases and disability [31]. Subjects were derived from the three different cohorts of the Rotterdam Study including RS-I (n = 2,436), RS-II (n = 750) and RS-III (n = 1,594) comprising individuals of Northwestern European Ancestry with available BMD-DXA measurements and GWAS data. Approval of the Medical Ethics Committee of the Erasmus University Rotterdam was obtained for the three cohorts of the Rotterdam Study. From all participants written informed consent was acquired.

### Bone mineral density measurements

Total body and Head BMD were measured in all participants using dual-energy X-ray absorptiometry (DXA) following standard manufacturer protocols. GE-Lunar iDXA was the devise used in the Generation R Study while the other cohorts employed GE Lunar (GE-Lunar Prodigy; GE Healthcare, Chalfont St Giles, UK). Bone mineral content (BMC) was derived from the projected bone area (BA) as BMC (mg) = BMD g/cm^2^×BA (cm^2^). As recommended by the International Society for Clinical Densitometry total body less head (TBLH) was the measurement used in the Generation R Study and ALSPAC instead of total body BMD [2].

### Genotype assessment

Genotyping was performed using the Illumina HumanHap 610 QUAD microarray in The Generation R, GOOD and RS-III cohorts while Illumina HumanHap 550 was the platform used for ALSPAC, RS-I and RS-II cohorts. Stringent quality control of the genotype and imputation process was performed in each study (Table S8). Samples with gender discrepancy, excess of heterozygosity or duplicates were excluded from analysis. *De novo* genotyping for the Generation R mothers was performed as part of a GEFOS initiative at Kbiosciences for specific SNPs of interest in the Osteoporosis field among those rs3801387.

### Imputation

For the imputation in the discovery Generation R cohort, we built a panel of reference haplotypes using HapMap phase II (release 22, build 36) CEU, YRI and CHB/JPT data. A two-step imputation process was performed, haplotype phasing and genotype imputation were carried out using MACH and minimac software, respectively. Imputation of the replication cohorts was done using MACH v1 based on the Phase II CEU HapMap data (release 22, build 36). Detailed descriptions of quality control and imputation procedures are summarized in Table S8.

### Statistical methods

Association between Total Body BMD and GWAS SNPs was carried out using a regression framework adjusting for age, gender, weight and population stratification in the Generation R discovery cohort using MACH2QTL as implemented in GRIMP [32]. Since this is a population-based study on unrelated individuals of different ethnic background, 20 genomic principal components obtained after SNP quality exclusion criteria and LD pruning were used to adjust for population sub-structure reaching a Genomic Inflation Factor (λ) of 1. We selected the most associated SNP and SNPs located at +/−500 kb from the top SNP for replication including all markers with a MAF>0.01 and an r^2^ imputation quality score >0.3 in all the participating studies. Additionally, rs3801387 a proxy of the ‘top hit’ (r^2^ = 1 in HapMap CEU populations) was genotyped in a subset of mothers of the Generation R Study of Dutch Northern European background. All replication cohorts included only individuals of North European ancestry and thus the correction for stratification was not as astringent as for the discovery cohort.

In the genome-wide association study, the association test of SNPs with standardized residuals of total body (skull) BMD after adjusting for age, gender, population stratification and weight (height) was implemented via Mach2QTL for all cohorts. Moreover, association in the mature adults and elderly cohorts, in which ample ranges of age are seen (Rotterdam Studies I, II, III and Mothers of Generation R) allowed for a non-linear relationship between age and BMD by inclusion of a squared term. On the conditional analysis we selected the most associated genotyped SNP (rs3801382) and applied a regression model including that marker besides the mentioned covariates in order to evaluate its effect in the originally detected signal(s).

We carried out regional meta-analyses in METAL using the minor allele from HapMap CEU genotypes as the coding allele, and applying inverse-variance methodology assuming fixed effects. A P value less than 5×10^−8^ was considered genome-wide significant (GWS). Heterogeneity was evaluated using Cochran's Q statistic and was quantified by I^2^. (http://www.sph.umich.edu/csg/abecasis/Metal/). For the meta-regression the absolute value of the effect size of the selected SNP (i.e. rs7801723, rs917727) in each trait was regressed on mean age of each of the six studies. These analyses were weighted by the inverse of variance of the effect; in line with the methodology applied for the meta-analyses.

### Knockout mice

*Wnt16* and *Fam3c* knockout (KO) mice were obtained from a program scrutinizing targets for drug discovery at Lexicon Pharmaceuticals. F2 hybrid littermates were derived from C57BL/6J and 129 SvEv parental strains. The 28 knockout mice (16 females) were generated by homologous recombination removing the first three exons of *Wnt16*. A three-fold strategy was used to generate *Fam3c* KO mice including a gene-trap (G-T) disrupting the intron between the first two exons of 6 mice (3 females); homologous recombination removing the first two exons (HR#1) of 8 mice (4 females); and homologous recombination replacing the whole gene of 8 mice (4 females) by the human gene (HR#2) resulting in loss of function. Confirmation of the exon disruption in *Wnt16* and *Fam3c* (HR#1) was achieved with Southern blot hybridization analysis while RT-PCR was used to confirm lack of gene expression of *Fam3c* (G-T) in KO mice (see Zheng et al. for details). Successful disruption of the *Fam3c* gene in all three KO strategies was demonstrated by a consistent hematological phenotype (data not shown). Male and female mice were scanned using a PIXImus DXA at 24 weeks (*Wnt16* KO comparison) and 14 weeks (*Fam3c* KO comparison) of age. BMD (and body composition) measurements were obtained from total body (excluding skull), femur and spine scans. Bone mineral content (BMC) was derived from the projected bone area (BA) as BMC (mg) = BMD mg/cm^2^×BA (cm^2^). Student's t-tests were used to assess statistical significance (P<0.05) of the differences within each sex.

### Gene transcripts expression levels from trans-iliacal bone biopsies

Gene expression profiles from all transcripts located within +/−500 kb of the rs917727 SNP in locus 7q31.31 were analyzed within an eQTL dataset of 78 Norwegian women, who make part of the set published by Reppe and colleagues [17]. Of these 78 women, 40 had osteoporosis (T-score less than −2.5), 7 had osteopenia (T-score between −2.5 and −1) and 31 were normal (T-score greater than −1) as ascertained by the BMD measurement at the total hip or lumbar spine (L1–L4 verterbrae). The Affymetrix HG U133 2.0 plus array was used for the expression analysis. The Affymetrix Cel files were imported into Partek Genomics Suite (Partek Inc., St Louis, MO, USA), and normalized using the RMA (Robust Multichip Average) algorithm. Further normalization was done by removing batch effects and patterns of gene expression levels due to differences in synthesis times across samples.

## Supporting Information

Figure S1 Forest plot for the genome wide association of the rs4609139 with TB-BMD. Results after conditioning on rs3801382, age, gender and weight. The results are reported per copy of the T-allele (MAF = 0.328–0.356).
(TIF)

Figure S2 Skull BMD Association plots for adults and children. A: SNP association plot for adult skull-BMD-associated region of Chromosome 7q31. B: SNP association plot for children skull-BMD-associated region of Chromosome 7q31. Genetic coordinates are as per Hapmap phase II-CEU.
(TIF)

Figure S3 Meta-regression for TB- and Skull BMD on rs7801723. Sample size weighted scatter plot of the absolute effect size versus the mean age of the studies for rs780123 in relation to A. skull and B. total body BMD.
(TIF)

Table S1 Characteristics of the participants in the complete discovery cohort overall and by ethnicity. Characteristics of subjects from the most numerous ethnicities defined according the classification of Statistics Netherlands.
(PDF)

Table S2 Characteristics of participants from the replication cohorts.
(PDF)

Table S3 SNPs showing GWS association with TB-BMD after conditioning by rs3801382.
(PDF)

Table S4 Evaluation of the covariates in the Generation R Study. The reduction of SE when weight is included in the model, allows the identification of the genetic signal mapping to 7q31, here represented by rs917727.
(PDF)

Table S5 SNPs showing GWS association with skull BMD.
(PDF)

Table S6 *Fam3c* KO mouse data for each knockout strategy. Gene trap and two types of homologous recombination: 1 and 2 (HR #1, HR #2).
(PDF)

Table S7 SNPs showing association with TB-BMD in the discovery cohort, overall and by ethnic clustering.
(PDF)

Table S8 Information of genetic data for each of the study cohorts. Genotyping methods, quality control of SNPs, imputation, and statistical analysis for the genome-wide association studies.
(PDF)
