# Supplementary figures and images for: Meta-Analysis of Genome-Wide Scans for Total Body BMD in Children and Adults Reveals Allelic Heterogeneity and Age-Specific Effects at the WNT16 Locus
Source: PLoS Genet. 2012 Jul 5;8(7):e1002718. doi: 10.1371/journal.pgen.1002718 (PMC3390371; doi:10.1371/journal.pgen.1002718)

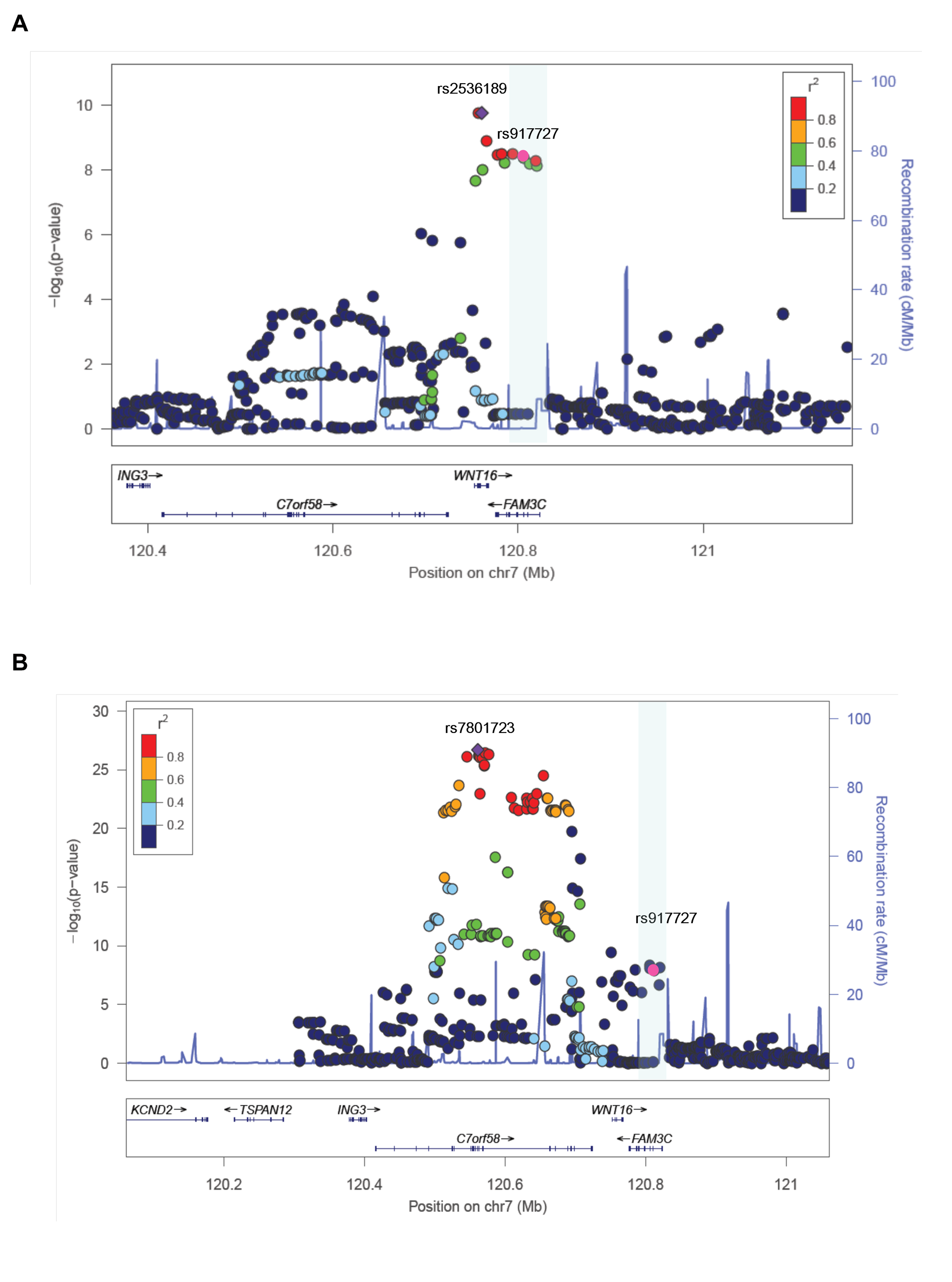

Supplement: Figure S1 — Forest plot for the genome wide association of the rs4609139 with TB-BMD. Results after conditioning on rs3801382, age, gender and weight. The results are reported per copy of the T-allele (MAF = 0.328–0.356). (TIF) [file pgen.1002718.s001.tif]

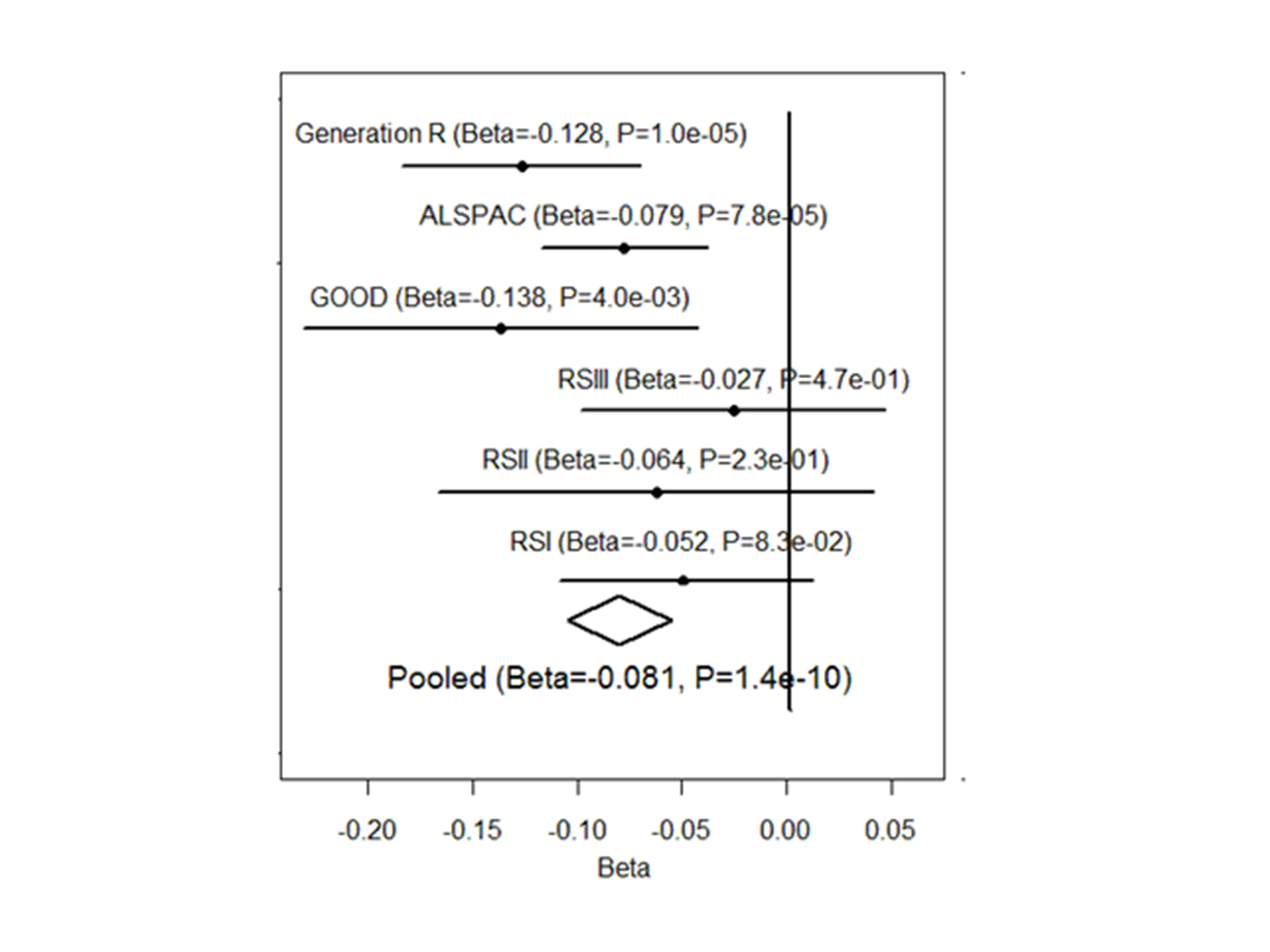

Supplement: Figure S2 — Skull BMD Association plots for adults and children. A: SNP association plot for adult skull-BMD-associated region of Chromosome 7q31. B: SNP association plot for children skull-BMD-associated region of Chromosome 7q31. Genetic coordinates are as per Hapmap phase II-CEU. (TIF) [file pgen.1002718.s002.tif]

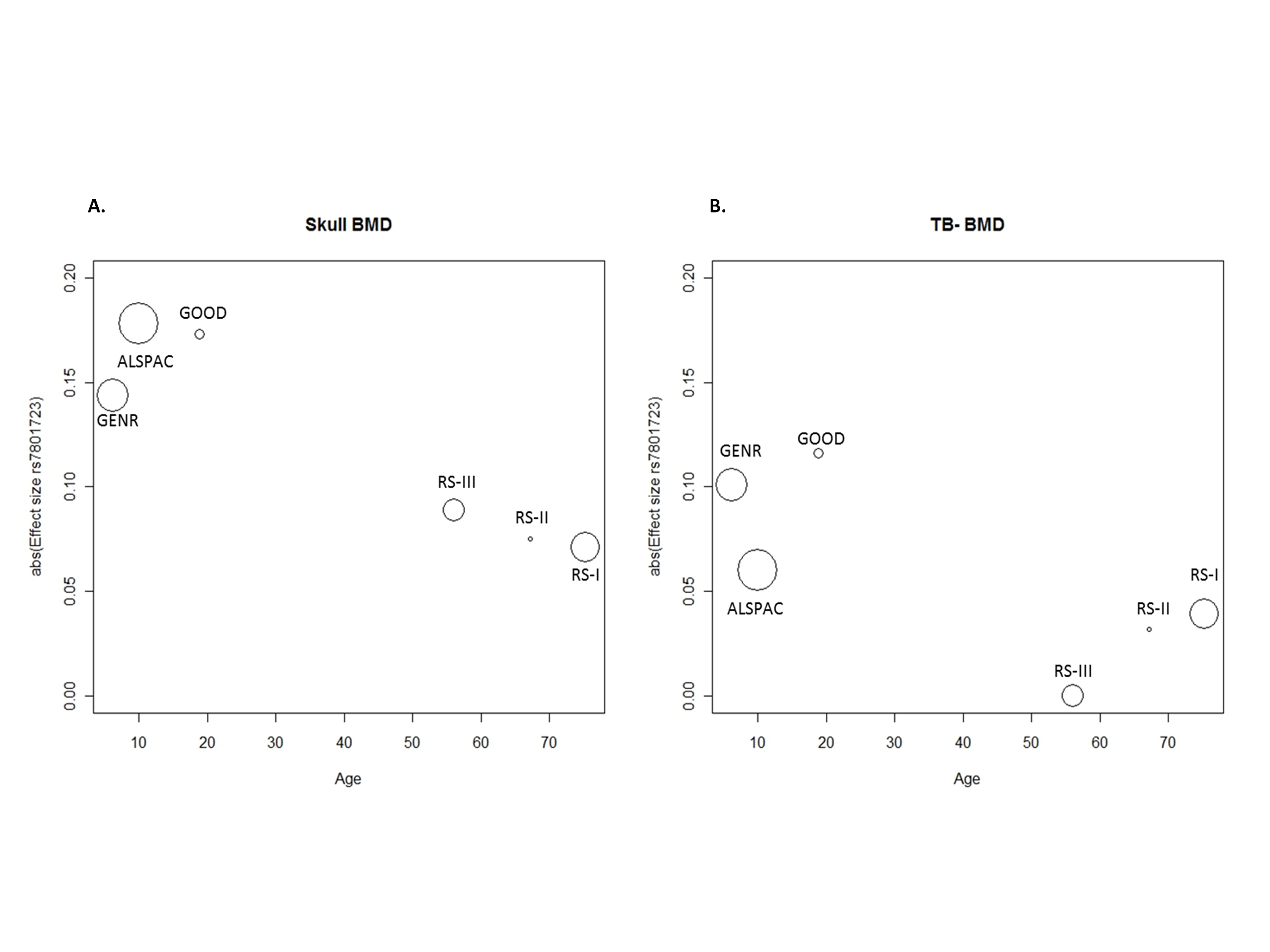

Supplement: Figure S3 — Meta-regression for TB- and Skull BMD on rs7801723. Sample size weighted scatter plot of the absolute effect size versus the mean age of the studies for rs780123 in relation to A. skull and B. total body BMD. (TIF) [file pgen.1002718.s003.tif]
