# Supplementary material for: Meta-Analysis of Genome-Wide Scans for Total Body BMD in Children and Adults Reveals Allelic Heterogeneity and Age-Specific Effects at the WNT16 Locus
Source: PLoS Genet. 2012 Jul 5;8(7):e1002718. doi: 10.1371/journal.pgen.1002718 (PMC3390371; doi:10.1371/journal.pgen.1002718)
Supplement: Table S1 — Characteristics of the participants in the complete discovery cohort overall and by ethnicity. Characteristics of subjects from the most numerous ethnicities defined according the classification of Statistics Netherlands. (PDF) [file pgen.1002718.s004.pdf]

	Dutch-Other European		Surinam		Turkish		Morrocan		Other		All GEN-R*	
	n=1,834		n=168		n=158		n=131		n=368		n=2,660	
**Age, years**	6.15	(0.36)	6.19	(0.42)	6.2	(0.42)	6.29	(0.53)	6.22	(0.41)	6.16	(0.28)
**Women, %**	934	50.87%	82	48.81%	74	46.84%	66	50.38%	191	51.90%	1,347	50.60%
**N. E ancestry %**	1,459	79.47%	3	1.79%	1	0.63%	1	0.76%	47	12.77%	1,511	69%
**Height, m**	1.19	(0.05)	1.19	(0.06)	1.18	(0.05)	1.19	(0.05)	1.19	(0.06)	1.19	(0.05)
**Weight, kg**	22.64	(3.33)	22.84	(5.19)	22.84	(4.52)	23.78	(4.07)	23.2	(3.96)	22.84	(3.54)
***Total body BMD, g/cm2**	0.55	(0.044)	0.56	(0.056)	0.55	(0.053)	0.56	(0.054)	0.57	(0.052)	0.55	(0.046)
**Total body BMC, g**	514	(87.23)	524.9	(111.4)	520.5	(103.61)	538.5	(105.66)	534.8	(109.02)	519.1	(91.14)

Mean (SD) except for n and percentages. *GEN-R, ALSPAC= Total body refers to Headless Total Body
