# Supplementary material for: Meta-Analysis of Genome-Wide Scans for Total Body BMD in Children and Adults Reveals Allelic Heterogeneity and Age-Specific Effects at the WNT16 Locus
Source: PLoS Genet. 2012 Jul 5;8(7):e1002718. doi: 10.1371/journal.pgen.1002718 (PMC3390371; doi:10.1371/journal.pgen.1002718)
Supplement: Table S2 — Characteristics of participants from the replication cohorts. (PDF) [file pgen.1002718.s005.pdf]

	ALSPAC*		GOOD		MoGENR		RS3		RS2		RS1	
	n=5,334		n=938		n=1,014		n=1,594		n=750		n=2,436	
**Age, years**	9.94	(0.32)	18.90	(0.556)	38.32	(4.23)	56.12	(5.77)	67.16	(6.44)	75.29	(5.49)
**Women, %**	2735	51.27%	0.00	0.00%	1014	100.00%	914	57.34%	426	56.72%	1385	56.85%
**Height, m**	1.4	(0.64)	1.81	(0.66)	1.7	(0.07)	1.709	(0.09)	1.69	(0.09)	1.67	(0.09)
**Weight, kg**	34.7	(7.42)	73.89	(11.62)	70.91	(12.88)	80	(14.93)	77.25	(12.76)	76.08	(15.41)
***Total body BMD, g/cm2**	0.777	(0.053)	1.25	(0.10)	1.176	(0.09)	1.1835	(0.11)	1.15	(0.11)	1.11	(0.11)
**Total body BMC, g**	891.34	(181.88)	3211.11	(199.78)	2586	(324.45)	2932	(591.04)	2779.98	(599.88)	2605.1	(592.44)

Mean (SD) except for n and percentages. *GEN-R, ALSPAC= Total body refers to Headless Total Body

Supplementary Table 3. SNPs showing GWS association with TB-BMD after conditioning by rs3801382

			DISCOVERY				REPLICATION														COMBINED			
VARIANT			Generation R			ALSPAC			GOOD			RS-III			RS-II			RS-I						
			n= 2,660			n= 5,334			n= 938			n= 1,594			n= 750			n= 2,436			n=13,712			
SNP	A1	R2*	Freq.	BETA**	P	Freq	BETA**	P	Freq	BETA**	P	Freq	BETA**	P	Freq	BETA**	P	Freq	BETA**	P	BETA**	P	I2	HetP
**rs4609139**	T	1.00	0.355	-0.128	8.81E-06	0.356	-0.079	9.72E-05	0.328	-0.138	0.004	0.351	-0.027	0.472	0.341	-0.064	0.225	0.354	-0.052	0.081	-0.081	1.42E-10	31	0.205
rs4731006	T	1.00	0.346	-0.123	1.89E-05	0.356	-0.079	9.80E-05	0.327	-0.139	0.004	0.350	-0.027	0.465	0.340	-0.065	0.221	0.354	-0.053	0.080	-0.080	1.99E-10	25	0.248
rs6967129	G	1.00	0.346	-0.122	2.13E-05	0.353	-0.078	1.15E-04	0.327	-0.142	0.003	0.350	-0.028	0.452	0.340	-0.067	0.211	0.353	-0.053	0.076	-0.080	2.14E-10	25	0.250
rs12706334	G	1.00	0.346	-0.122	2.17E-05	0.353	-0.078	1.12E-04	0.326	-0.142	0.003	0.350	-0.028	0.454	0.340	-0.066	0.214	0.353	-0.053	0.077	-0.080	2.21E-10	25	0.250
rs798943	A	0.56	0.384	-0.117	2.32E-05	0.386	-0.080	6.02E-05	0.333	-0.139	0.004	0.383	-0.014	0.699	0.367	-0.066	0.206	0.393	-0.055	0.061	-0.078	2.59E-10	31	0.204
**rs7801723**	T	0.56	0.374	-0.117	2.39E-05	0.386	-0.080	6.10E-05	0.333	-0.140	0.004	0.383	-0.014	0.698	0.367	-0.066	0.204	0.393	-0.055	0.060	-0.078	2.72E-10	33	0.189
rs13232048	T	0.56	0.378	-0.117	2.59E-05	0.386	-0.080	6.32E-05	0.333	-0.140	0.004	0.383	-0.014	0.698	0.367	-0.066	0.204	0.393	-0.055	0.060	-0.078	2.72E-10	33	0.189
rs12706318	G	0.56	0.378	-0.117	2.59E-05	0.386	-0.080	6.25E-05	0.333	-0.140	0.004	0.383	-0.014	0.698	0.367	-0.066	0.204	0.393	-0.055	0.060	-0.078	2.72E-10	33	0.189
rs6950680	G	0.56	0.374	-0.118	2.14E-05	0.385	-0.079	6.97E-05	0.333	-0.140	0.004	0.383	-0.013	0.715	0.367	-0.067	0.203	0.393	-0.055	0.061	-0.078	3.10E-10	35	0.177
rs872007	T	0.56	0.379	-0.117	2.69E-05	0.385	-0.079	6.78E-05	0.333	-0.140	0.004	0.383	-0.014	0.699	0.367	-0.067	0.203	0.393	-0.055	0.060	-0.078	3.23E-10	33	0.190
rs6952113	A	0.56	0.379	-0.117	2.67E-05	0.385	-0.079	6.49E-05	0.333	-0.140	0.004	0.383	-0.014	0.699	0.367	-0.066	0.204	0.393	-0.055	0.060	-0.078	3.32E-10	33	0.189
rs13245690	G	0.56	0.376	-0.117	2.84E-05	0.387	-0.080	5.56E-05	0.337	-0.137	0.005	0.385	-0.015	0.689	0.370	-0.064	0.220	0.396	-0.053	0.070	-0.078	3.37E-10	30	0.208
rs10261671	T	0.46	0.388	-0.115	3.66E-05	0.386	-0.080	5.82E-05	0.334	-0.139	0.004	0.383	-0.012	0.734	0.367	-0.067	0.201	0.393	-0.054	0.069	-0.078	4.19E-10	31	0.204
rs10275439	A	0.53	0.386	-0.116	2.58E-05	0.383	-0.079	8.07E-05	0.330	-0.141	0.004	0.380	-0.012	0.750	0.362	-0.062	0.234	0.390	-0.055	0.063	-0.078	4.57E-10	33	0.191
rs10500083	C	0.93	0.351	-0.122	2.41E-05	0.350	-0.075	2.53E-04	0.326	-0.141	0.003	0.347	-0.025	0.511	0.336	-0.067	0.209	0.350	-0.053	0.078	-0.078	4.87E-10	27	0.233
rs2272196	A	0.93	0.351	-0.121	2.45E-05	0.350	-0.075	2.45E-04	0.326	-0.142	0.003	0.347	-0.025	0.512	0.336	-0.067	0.211	0.350	-0.053	0.079	-0.078	5.17E-10	26	0.237
rs7795692	G	0.93	0.351	-0.121	2.47E-05	0.350	-0.075	2.40E-04	0.326	-0.142	0.003	0.347	-0.025	0.513	0.336	-0.066	0.213	0.350	-0.053	0.079	-0.078	5.32E-10	26	0.236
rs10480747	C	0.93	0.341	-0.120	3.46E-05	0.351	-0.075	2.60E-04	0.326	-0.141	0.004	0.347	-0.025	0.510	0.336	-0.067	0.207	0.350	-0.053	0.077	-0.078	5.89E-10	25	0.250
rs7795660	T	0.93	0.344	-0.119	3.46E-05	0.350	-0.075	2.42E-04	0.326	-0.142	0.003	0.347	-0.025	0.513	0.336	-0.067	0.212	0.350	-0.053	0.079	-0.078	6.26E-10	24	0.253
rs7778938	C	0.93	0.343	-0.118	4.35E-05	0.350	-0.075	2.39E-04	0.326	-0.142	0.003	0.347	-0.025	0.513	0.336	-0.066	0.213	0.350	-0.053	0.080	-0.078	7.07E-10	23	0.261
rs11765163	T	0.93	0.344	-0.118	4.15E-05	0.351	-0.074	2.68E-04	0.327	-0.141	0.004	0.348	-0.025	0.509	0.337	-0.068	0.203	0.350	-0.054	0.076	-0.077	7.73E-10	22	0.270
rs11771945	G	0.93	0.344	-0.118	4.15E-05	0.351	-0.074	2.72E-04	0.327	-0.140	0.004	0.348	-0.025	0.509	0.337	-0.068	0.203	0.350	-0.054	0.076	-0.077	8.00E-10	21	0.275
rs7792071	G	0.84	0.446	-0.127	4.51E-06	0.436	-0.057	0.004	0.405	-0.140	0.003	0.420	-0.049	0.174	0.408	-0.082	0.111	0.427	-0.045	0.121	-0.074	8.53E-10	37	0.159
rs6948725	C	0.87	0.440	-0.126	5.90E-06	0.435	-0.058	0.003	0.403	-0.143	0.002	0.419	-0.048	0.184	0.408	-0.080	0.118	0.426	-0.045	0.126	-0.074	9.00E-10	36	0.165
rs6979948	T	0.84	0.435	-0.121	1.60E-05	0.436	-0.057	0.003	0.404	-0.141	0.002	0.420	-0.049	0.177	0.408	-0.081	0.115	0.426	-0.045	0.123	-0.073	1.76E-09	30	0.210
rs11509199	T	0.87	0.432	-0.119	2.07E-05	0.435	-0.058	0.003	0.403	-0.142	0.002	0.419	-0.048	0.181	0.408	-0.081	0.116	0.426	-0.045	0.125	-0.073	1.78E-09	28	0.227

Variant			Generation R			ALSPAC			GOOD			RS-III			RS-II			RS-I			Combined			
SNP	A1	R2*	Freq.	BETA**	P	Freq	BETA**	P	Freq	BETA**	P	Freq	BETA**	P	Freq	BETA**	P	Freq	BETA**	P	BETA**	P	I2	HetP
rs10953933	T	0.87	0.432	-0.119	2.08E-05	0.435	-0.058	0.003	0.403	-0.142	0.002	0.419	-0.048	0.182	0.408	-0.081	0.116	0.426	-0.045	0.125	-0.073	1.78E-09	28	0.227
rs7805374	G	0.84	0.440	-0.119	2.09E-05	0.436	-0.057	0.004	0.405	-0.140	0.003	0.420	-0.049	0.173	0.408	-0.082	0.110	0.427	-0.045	0.121	-0.073	1.84E-09	28	0.226
rs1534019	T	0.84	0.440	-0.119	2.06E-05	0.437	-0.056	0.004	0.405	-0.139	0.003	0.420	-0.049	0.172	0.409	-0.083	0.108	0.427	-0.046	0.120	-0.072	1.98E-09	28	0.228
rs7808120	A	0.84	0.440	-0.119	2.07E-05	0.437	-0.056	0.004	0.405	-0.140	0.003	0.420	-0.049	0.173	0.408	-0.082	0.109	0.427	-0.046	0.120	-0.072	2.07E-09	28	0.224
rs6466774	C	0.84	0.439	-0.119	2.10E-05	0.436	-0.057	0.004	0.404	-0.141	0.003	0.420	-0.049	0.176	0.408	-0.081	0.114	0.426	-0.045	0.122	-0.073	2.13E-09	28	0.228
rs6942652	C	0.87	0.434	-0.117	2.50E-05	0.435	-0.058	0.003	0.403	-0.142	0.002	0.419	-0.048	0.181	0.408	-0.081	0.115	0.426	-0.045	0.125	-0.072	2.15E-09	25	0.245
rs10266975	C	0.87	0.434	-0.117	2.53E-05	0.435	-0.058	0.003	0.403	-0.142	0.002	0.419	-0.048	0.183	0.408	-0.081	0.117	0.426	-0.045	0.126	-0.072	2.15E-09	25	0.245
rs12706333	T	0.87	0.434	-0.117	2.53E-05	0.435	-0.058	0.003	0.403	-0.142	0.002	0.419	-0.048	0.182	0.408	-0.081	0.117	0.426	-0.045	0.126	-0.072	2.15E-09	25	0.245
rs10225276	G	0.87	0.434	-0.117	2.54E-05	0.435	-0.058	0.003	0.403	-0.142	0.002	0.419	-0.048	0.183	0.408	-0.081	0.118	0.426	-0.045	0.126	-0.072	2.15E-09	25	0.245
rs4731007	G	0.87	0.437	-0.120	1.62E-05	0.438	-0.058	0.003	0.404	-0.138	0.003	0.419	-0.046	0.199	0.409	-0.078	0.130	0.427	-0.043	0.137	-0.072	2.21E-09	29	0.215
rs6970383	C	0.87	0.435	-0.119	2.01E-05	0.438	-0.058	0.003	0.404	-0.140	0.003	0.419	-0.047	0.193	0.408	-0.079	0.125	0.426	-0.044	0.133	-0.072	2.22E-09	27	0.229
rs1534014	C	0.84	0.440	-0.119	2.08E-05	0.436	-0.056	0.004	0.405	-0.140	0.003	0.420	-0.049	0.173	0.408	-0.082	0.110	0.427	-0.045	0.120	-0.072	2.26E-09	29	0.219
rs12539571	C	0.87	0.434	-0.117	2.85E-05	0.435	-0.058	0.003	0.402	-0.143	0.002	0.419	-0.048	0.185	0.407	-0.080	0.120	0.426	-0.044	0.127	-0.072	2.33E-09	27	0.235
rs7806875	T	0.84	0.440	-0.118	2.30E-05	0.436	-0.057	0.003	0.404	-0.141	0.002	0.420	-0.049	0.177	0.408	-0.081	0.115	0.426	-0.045	0.123	-0.072	2.34E-09	26	0.237
rs6947934	C	0.87	0.437	-0.119	1.70E-05	0.438	-0.058	0.003	0.404	-0.138	0.003	0.419	-0.046	0.197	0.408	-0.078	0.128	0.427	-0.043	0.135	-0.072	2.43E-09	28	0.224
rs1534017	C	0.78	0.465	-0.120	1.21E-05	0.460	-0.052	0.007	0.424	-0.133	0.004	0.439	-0.042	0.237	0.427	-0.082	0.108	0.447	-0.050	0.089	-0.071	2.99E-09	32	0.197
rs6978080	A	0.78	0.464	-0.120	1.22E-05	0.460	-0.052	0.007	0.424	-0.133	0.004	0.439	-0.042	0.242	0.427	-0.082	0.109	0.447	-0.050	0.090	-0.071	2.99E-09	32	0.197
rs12672898	A	0.78	0.464	-0.120	1.21E-05	0.460	-0.052	0.007	0.424	-0.133	0.004	0.439	-0.042	0.240	0.427	-0.082	0.108	0.447	-0.050	0.089	-0.071	2.99E-09	32	0.197
rs10953932	G	0.78	0.464	-0.120	1.21E-05	0.460	-0.052	0.007	0.424	-0.132	0.004	0.439	-0.042	0.239	0.427	-0.082	0.108	0.447	-0.050	0.089	-0.071	3.09E-09	31	0.201
rs17357115	C	0.78	0.510	-0.119	1.56E-05	0.460	-0.052	0.007	0.424	-0.133	0.004	0.439	-0.042	0.241	0.427	-0.082	0.109	0.447	-0.050	0.089	-0.071	3.30E-09	31	0.206
rs17284918	G	0.78	0.509	-0.118	1.77E-05	0.460	-0.052	0.007	0.424	-0.133	0.004	0.439	-0.042	0.242	0.428	-0.081	0.117	0.447	-0.050	0.091	-0.071	3.94E-09	29	0.217
rs1524503	C	0.60	0.392	-0.128	8.65E-06	0.405	-0.071	4.55E-04	0.356	-0.139	0.004	0.396	-0.018	0.637	0.398	-0.058	0.279	0.416	-0.038	0.202	-0.074	5.24E-09	45	0.106
rs10085590	G	0.60	0.420	-0.121	1.05E-05	0.412	-0.068	5.30E-04	0.359	-0.139	0.003	0.411	-0.017	0.638	0.401	-0.061	0.244	0.421	-0.035	0.227	-0.072	5.44E-09	47	0.095
rs1554634	C	0.60	0.414	-0.120	1.08E-05	0.412	-0.068	5.30E-04	0.359	-0.139	0.003	0.411	-0.017	0.632	0.401	-0.063	0.236	0.420	-0.035	0.228	-0.072	5.71E-09	46	0.101
rs7798060	T	0.60	0.396	-0.121	1.07E-05	0.412	-0.069	4.10E-04	0.359	-0.139	0.003	0.412	-0.018	0.615	0.401	-0.065	0.215	0.421	-0.037	0.195	-0.072	5.91E-09	43	0.116
rs1524506	T	0.78	0.457	-0.111	5.31E-05	0.460	-0.053	0.006	0.424	-0.133	0.004	0.439	-0.042	0.237	0.427	-0.082	0.108	0.447	-0.050	0.089	-0.070	5.97E-09	18	0.295
rs6965195	A	0.86	0.361	-0.108	1.20E-04	0.374	-0.068	6.49E-04	0.345	-0.130	0.006	0.366	-0.017	0.635	0.355	-0.067	0.203	0.370	-0.057	0.056	-0.073	6.03E-09	12	0.338
rs1534016	C	0.86	0.361	-0.108	1.19E-04	0.374	-0.068	6.52E-04	0.345	-0.130	0.006	0.366	-0.017	0.635	0.355	-0.067	0.201	0.371	-0.057	0.055	-0.073	6.03E-09	12	0.338
rs6947494	T	0.60	0.421	-0.121	1.04E-05	0.412	-0.068	5.34E-04	0.359	-0.137	0.004	0.412	-0.015	0.672	0.401	-0.058	0.273	0.421	-0.035	0.222	-0.071	7.04E-09	47	0.093
rs1949803	G	0.75	0.509	-0.121	1.43E-05	0.460	-0.053	0.007	0.420	-0.140	0.003	0.441	-0.042	0.250	0.429	-0.080	0.124	0.450	-0.047	0.115	-0.072	7.97E-09	33	0.188
rs7797976	T	0.60	0.398	-0.123	7.98E-06	0.412	-0.068	5.32E-04	0.359	-0.138	0.004	0.412	-0.016	0.668	0.401	-0.058	0.270	0.421	-0.035	0.223	-0.071	9.75E-09	47	0.093

Variant			Generation R			ALSPAC			GOOD			RS-III			RS-II			RS-I			Combined			
SNP	A1	R2*	Freq.	BETA**	P	Freq	BETA**	P	Freq	BETA**	P	Freq	BETA**	P	Freq	BETA**	P	Freq	BETA**	P	BETA**	P	I2	HetP
rs1917118	T	0.60	0.410	-0.123	9.10E-06	0.412	-0.068	5.38E-04	0.359	-0.137	0.004	0.412	-0.015	0.679	0.401	-0.057	0.282	0.421	-0.035	0.221	-0.071	1.09E-08	47	0.092
rs11770502	G	0.79	0.453	-0.110	1.30E-04	0.450	-0.057	0.004	0.423	-0.144	0.003	0.437	-0.050	0.176	0.426	-0.090	0.086	0.447	-0.044	0.138	-0.072	1.19E-08	15	0.319
rs6954210	A	0.60	0.410	-0.123	9.23E-06	0.412	-0.068	5.47E-04	0.360	-0.135	0.004	0.412	-0.014	0.697	0.402	-0.055	0.295	0.421	-0.036	0.218	-0.071	1.19E-08	47	0.095
rs6947453	T	0.51	0.377	-0.107	1.59E-04	0.388	-0.077	1.93E-04	0.345	-0.150	0.003	0.383	-0.017	0.645	0.369	-0.048	0.378	0.393	-0.039	0.200	-0.073	1.42E-08	34	0.183
rs1404268	A	0.60	0.427	-0.118	3.68E-05	0.406	-0.071	4.62E-04	0.357	-0.138	0.005	0.396	-0.018	0.638	0.402	-0.053	0.318	0.416	-0.038	0.202	-0.071	1.50E-08	37	0.163
rs6970762	T	0.60	0.400	-0.122	1.02E-05	0.412	-0.068	5.49E-04	0.360	-0.130	0.006	0.412	-0.014	0.701	0.402	-0.055	0.298	0.421	-0.036	0.217	-0.070	1.54E-08	44	0.111
rs1357756	T	0.60	0.421	-0.121	1.08E-05	0.412	-0.068	5.51E-04	0.360	-0.130	0.006	0.412	-0.014	0.708	0.402	-0.054	0.305	0.421	-0.036	0.217	-0.070	1.73E-08	44	0.116
rs1534015	A	0.60	0.421	-0.121	1.09E-05	0.412	-0.068	5.54E-04	0.360	-0.129	0.006	0.412	-0.013	0.715	0.402	-0.053	0.316	0.421	-0.036	0.216	-0.069	1.94E-08	44	0.114
rs2968349	C	0.47	0.457	-0.101	2.24E-04	0.468	-0.070	2.89E-04	0.374	-0.120	0.013	0.436	-0.037	0.301	0.428	-0.055	0.282	0.454	-0.026	0.371	-0.067	2.23E-08	11	0.345
rs7786203	A	0.60	0.401	-0.121	1.19E-05	0.412	-0.067	6.66E-04	0.360	-0.129	0.006	0.412	-0.012	0.734	0.402	-0.052	0.321	0.422	-0.036	0.216	-0.069	2.51E-08	44	0.109

Shaded rs7801723 top-hit for association with Head BMD *Correlation coefficients with rs4609139 based on HapMap release22 CEU population.**Effect estimates expressed as standardized adjusted SD per copy of allele (A1).

Supplementary Table 4. Evaluation of the covariates in the Generation R Study. The reduction of SE when weight is included in the model, allows the identification of the genetic signal mapping to 7q31, here represented by rs917727

Predictor variables	Model 0 (M0)		Model 1 (M1)		Final Model (MF)	
	Beta	P	Beta	P	Beta	P
**rs9117727**	0.131	6.61E-13	0.097	**1.02E-07**	0.090	**5.90E-11**
**Age (Years)**	0.337	< 2.00E-16	0.317	**< 2.00E-16**	0.101	**3.52E-12**
**Sex (Male)**	0.023	0.209	0.020	**2.55E-01**	0.160	0.242
**Weight (Kg)**	-	-	-	**-**	0.646	**< 2.00E-16**
**PC1**	-	-	-0.149	**9.25E-16**	-0.104	**1.28E-13**
**PC2**	-	-	0.100	**2.37E-08**	0.046	**0.001**
**PC3**	-	-	-0.010	**5.84E-01**	-0.038	**0.005**
**PC4**	-	-	0.029	0.101	0.005	0.717
**PC5**	-	-	-0.025	0.162	0.007	0.586
**PC6**	-	-	-0.027	0.135	-0.034	**0.013**
**PC7**	-	-	-0.004	0.801	-0.001	0.936
**PC8**	-	-	0.025	0.162	0.024	0.075
**PC9**	-	-	0.033	0.065	0.033	**0.015**
**PC10**	-	-	-0.012	0.516	0.007	0.621
**PC11**	-	-	-0.008	0.648	-0.010	0.466
**PC12**	-	-	-0.017	0.345	-0.010	0.468
**PC13**	-	-	0.027	0.127	0.022	0.1
**PC14**	-	-	-0.018	0.313	-0.007	0.605
**PC15**	-	-	0.025	0.163	0.020	0.143
**PC16**	-	-	0.000	0.99	-0.020	0.134
**PC17**	-	-	0.001	0.954	0.004	0.762
**PC18**	-	-	0.001	0.966	-0.019	0.164
**PC19**	-	-	-0.007	0.691	0.002	0.891
**PC20**	-	-	-0.007	0.692	-0.001	0.954

Supplementary Table 5. SNPs showing GWS association with skull BMD

			DISCOVERY			REPLICATION															COMBINED			
VARIANT			Generation R			ALSPAC			GOOD			RS-III			RS-II			RS-I						
			n= 2,660			n= 5,334			n= 938			n= 1,594			n= 750			n= 2,436			n=13,712			
SNP	A1	R2*	Freq.	BETA**	P	Freq	BETA**	P	Freq	BETA**	P	Freq	BETA**	P	Freq	BETA**	P	Freq	BETA**	P	BETA**	P	I2	HetP
**rs7801723**	T	1	0.374	-0.144	2.75E-07	0.386	-0.178	**6.00E-19**	0.333	-0.173	3.42E-04	0.383	-0.089	0.014	0.367	-0.075	0.154	0.393	-0.071	0.017	-0.137	**8.91E-28**	61	0.026
rs6950680	G	1	0.374	-0.144	2.62E-07	0.385	-0.176	**1.22E-18**	0.333	-0.173	3.39E-04	0.383	-0.09	0.014	0.367	-0.075	0.154	0.393	-0.07	0.017	-0.136	**1.86E-27**	60	0.030
rs12706318	G	1	0.378	-0.139	6.79E-07	0.386	-0.178	**6.70E-19**	0.333	-0.173	3.42E-04	0.383	-0.089	0.014	0.367	-0.075	0.154	0.393	-0.071	0.017	-0.136	**2.14E-27**	61	0.027
rs13232048	T	1	0.378	-0.139	6.80E-07	0.386	-0.178	**7.00E-19**	0.333	-0.173	3.42E-04	0.383	-0.089	0.014	0.367	-0.075	0.154	0.393	-0.071	0.017	-0.136	**2.14E-27**	61	0.027
rs798943	A	1	0.384	-0.138	8.13E-07	0.386	-0.178	**6.18E-19**	0.333	-0.173	3.37E-04	0.383	-0.089	0.015	0.367	-0.075	0.155	0.393	-0.071	0.017	-0.135	**2.54E-27**	60	0.027
rs6952113	A	1	0.379	-0.139	6.80E-07	0.385	-0.177	**7.86E-19**	0.333	-0.173	3.41E-04	0.383	-0.09	0.014	0.367	-0.075	0.154	0.393	-0.071	0.017	-0.135	**2.72E-27**	60	0.030
rs872007	T	1	0.379	-0.139	6.88E-07	0.385	-0.177	**9.53E-19**	0.333	-0.173	3.41E-04	0.383	-0.09	0.014	0.367	-0.075	0.154	0.393	-0.071	0.017	-0.135	**2.72E-27**	60	0.030
rs13245690	G	0.93	0.376	-0.144	2.90E-07	0.387	-0.177	**1.06E-18**	0.337	-0.172	3.44E-04	0.385	-0.089	0.015	0.370	-0.071	0.176	0.396	-0.067	0.023	-0.134	**2.98E-27**	63	0.018
rs10275439	A	0.90	0.386	-0.134	1.55E-06	0.383	-0.176	**1.48E-18**	0.330	-0.172	3.83E-04	0.380	-0.088	0.017	0.362	-0.072	0.172	0.390	-0.071	0.017	-0.133	**1.35E-26**	60	0.030
rs10261671	T	0.80	0.388	-0.134	1.83E-06	0.386	-0.176	**1.35E-18**	0.334	-0.173	3.55E-04	0.383	-0.088	0.016	0.367	-0.072	0.171	0.393	-0.069	0.020	-0.133	**1.72E-26**	61	0.027
rs2536150	C	0.10	0.215	-0.2	**4.58E-09**	0.176	-0.173	**1.27E-11**	0.168	-0.173	0.006	0.180	-0.104	0.026	0.174	-0.166	0.017	0.182	-0.143	0.000	-0.165	**2.59E-25**	0	0.671
rs1524503	C	0.77	0.392	-0.133	4.61E-06	0.405	-0.172	**3.63E-17**	0.356	-0.186	1.44E-04	0.396	-0.095	0.011	0.398	-0.073	0.176	0.416	-0.054	0.071	-0.130	**4.66E-25**	65	0.015
rs13223036	G	0.62	0.345	-0.137	1.49E-06	0.371	-0.17	**3.09E-17**	0.299	-0.147	0.004	0.351	-0.081	0.028	0.340	-0.057	0.286	0.361	-0.073	0.015	-0.129	**1.05E-24**	56	0.045
rs7798060	T	0.77	0.396	-0.122	1.03E-05	0.412	-0.165	**8.43E-17**	0.359	-0.177	1.80E-04	0.412	-0.1	0.006	0.401	-0.088	0.099	0.421	-0.059	0.044	-0.126	**1.60E-24**	56	0.044
rs7797976	T	0.77	0.398	-0.123	9.58E-06	0.412	-0.164	**1.36E-16**	0.359	-0.177	1.79E-04	0.412	-0.098	0.007	0.401	-0.082	0.124	0.421	-0.055	0.057	-0.125	**5.44E-24**	59	0.034
rs6970762	T	0.77	0.400	-0.123	1.07E-05	0.412	-0.164	**1.41E-16**	0.360	-0.176	1.94E-04	0.412	-0.097	0.007	0.402	-0.079	0.140	0.421	-0.055	0.060	0.125	**7.25E-24**	59	0.033
rs1404268	A	0.77	0.427	-0.117	5.17E-05	0.406	-0.171	**3.81E-17**	0.357	-0.184	1.50E-04	0.396	-0.095	0.011	0.402	-0.076	0.155	0.416	-0.054	0.071	-0.127	**7.29E-24**	64	0.016
rs1917118	T	0.77	0.410	-0.119	1.97E-05	0.412	-0.164	**1.37E-16**	0.359	-0.177	1.79E-04	0.412	-0.098	0.007	0.401	-0.081	0.130	0.421	-0.055	0.058	-0.124	**1.08E-23**	59	0.033
rs6954210	A	0.77	0.410	-0.119	1.93E-05	0.412	-0.164	**1.40E-16**	0.360	-0.177	1.81E-04	0.412	-0.098	0.007	0.402	-0.079	0.138	0.421	-0.055	0.060	-0.124	**1.18E-23**	59	0.032
rs6947453	T	0.93	0.377	-0.128	7.62E-06	0.388	-0.177	**1.66E-17**	0.345	-0.175	4.76E-04	0.383	-0.088	0.020	0.369	-0.08	0.148	0.393	-0.057	0.063	-0.129	**1.47E-23**	64	0.017
rs1554634	C	0.77	0.414	-0.113	4.03E-05	0.412	-0.164	**1.09E-16**	0.359	-0.177	1.80E-04	0.411	-0.099	0.006	0.401	-0.086	0.104	0.420	-0.056	0.054	-0.123	**1.76E-23**	58	0.036
rs7786203	A	0.77	0.401	-0.119	1.96E-05	0.412	-0.164	**1.24E-16**	0.360	-0.176	1.97E-04	0.412	-0.096	0.008	0.402	-0.076	0.154	0.422	-0.054	0.064	-0.123	**2.01E-23**	60	0.029
rs10085590	G	0.77	0.420	-0.111	5.44E-05	0.412	-0.164	**1.16E-16**	0.359	-0.177	1.80E-04	0.411	-0.099	0.006	0.401	-0.085	0.109	0.421	-0.056	0.054	-0.123	**2.53E-23**	58	0.035
rs6947494	T	0.77	0.421	-0.112	4.97E-05	0.412	-0.164	**1.37E-16**	0.359	-0.177	1.79E-04	0.412	-0.098	0.007	0.401	-0.082	0.125	0.421	-0.055	0.057	-0.123	**3.14E-23**	59	0.031
rs1357756	T	0.77	0.421	-0.113	4.81E-05	0.412	-0.164	**1.41E-16**	0.360	-0.176	1.95E-04	0.412	-0.097	0.007	0.402	-0.078	0.143	0.421	-0.055	0.061	-0.123	**3.72E-23**	60	0.030
rs1534015	A	0.77	0.421	-0.113	4.80E-05	0.412	-0.164	**1.42E-16**	0.360	-0.176	1.97E-04	0.412	-0.097	0.007	0.402	-0.076	0.150	0.421	-0.054	0.061	-0.122	**4.71E-23**	60	0.028
rs2952559	C	0.09	0.265	-0.186	**2.51E-08**	0.183	-0.158	**7.29E-10**	0.165	-0.174	0.007	0.180	-0.128	0.007	0.184	-0.147	0.035	0.184	-0.128	0.001	-0.156	**6.22E-23**	0	0.873

Variant			Generation R			ALSPAC			GOOD			RS-III			RS-II			RS-I			Combined			
SNP	A1	R2*	Freq.	BETA**	P	Freq	BETA**	P	Freq	BETA**	P	Freq	BETA**	P	Freq	BETA**	P	Freq	BETA**	P	BETA**	P	I2	HetP
rs6954757	A	0.56	0.339	-0.136	1.55E-06	0.364	-0.161	**1.38E-15**	0.295	-0.15	0.003	0.341	-0.079	0.034	0.329	-0.027	0.621	0.350	-0.074	0.014	-0.123	**8.52E-23**	56	0.045
rs6466769	G	0.56	0.339	-0.136	1.62E-06	0.364	-0.16	**1.51E-15**	0.295	-0.15	0.003	0.341	-0.078	0.035	0.330	-0.026	0.627	0.351	-0.073	0.016	-0.123	**1.53E-22**	56	0.044
rs1534016	C	0.68	0.361	-0.141	6.61E-07	0.374	-0.154	**2.28E-14**	0.345	-0.182	1.21E-04	0.366	-0.084	0.023	0.355	-0.019	0.715	0.371	-0.058	0.054	-0.121	**2.29E-22**	64	0.015
rs6965195	A	0.68	0.361	-0.141	6.66E-07	0.374	-0.154	**2.28E-14**	0.345	-0.182	1.22E-04	0.366	-0.084	0.023	0.355	-0.019	0.717	0.370	-0.058	0.054	-0.121	**2.29E-22**	64	0.015
rs1917113	A	0.56	0.339	-0.136	1.64E-06	0.364	-0.159	**2.37E-15**	0.296	-0.151	0.003	0.341	-0.077	0.038	0.331	-0.024	0.662	0.352	-0.069	0.023	-0.122	**3.43E-22**	58	0.036
rs6967129	G	0.56	0.346	-0.148	3.59E-07	0.353	-0.156	**2.08E-14**	0.327	-0.149	0.002	0.350	-0.094	0.012	0.340	-0.017	0.749	0.353	-0.058	0.054	-0.122	**3.88E-22**	61	0.024
rs12706334	G	0.56	0.346	-0.147	3.78E-07	0.353	-0.156	**2.32E-14**	0.326	-0.148	0.002	0.350	-0.094	0.012	0.340	-0.017	0.753	0.353	-0.058	0.054	-0.122	**4.06E-22**	61	0.025
rs12673968	A	0.56	0.347	-0.132	3.19E-06	0.364	-0.159	**2.37E-15**	0.296	-0.151	0.003	0.341	-0.077	0.038	0.331	-0.024	0.662	0.352	-0.069	0.023	-0.121	**6.41E-22**	58	0.038
rs4731006	T	0.56	0.346	-0.148	3.85E-07	0.356	-0.154	**4.20E-14**	0.327	-0.146	0.002	0.350	-0.094	0.012	0.340	-0.016	0.760	0.354	-0.058	0.054	-0.121	**7.51E-22**	60	0.028
rs6466767	G	0.56	0.348	-0.133	2.93E-06	0.364	-0.159	**2.30E-15**	0.296	-0.151	0.003	0.342	-0.075	0.044	0.331	-0.019	0.723	0.354	-0.068	0.026	-0.120	**9.25E-22**	60	0.030
**rs4609139**	T	0.56	0.355	-0.147	3.52E-07	0.356	-0.154	**4.88E-14**	0.328	-0.145	0.003	0.351	-0.093	0.013	0.341	-0.016	0.764	0.354	-0.058	0.054	-0.121	**1.00E-21**	60	0.028
rs2968345	G	0.56	0.359	-0.133	2.81E-06	0.365	-0.159	**2.28E-15**	0.296	-0.151	0.003	0.342	-0.073	0.050	0.332	-0.017	0.748	0.355	-0.066	0.028	-0.120	**1.57E-21**	61	0.024
rs10500083	C	0.62	0.351	-0.154	1.04E-07	0.350	-0.155	**4.26E-14**	0.326	-0.164	0.001	0.347	-0.09	0.017	0.336	-0.025	0.639	0.350	-0.056	0.067	-0.122	**1.91E-21**	62	0.021
rs2272196	A	0.62	0.351	-0.154	1.08E-07	0.350	-0.155	**4.45E-14**	0.326	-0.163	7.28E-04	0.347	-0.09	0.017	0.336	-0.025	0.642	0.350	-0.056	0.067	-0.122	**2.02E-21**	62	0.022
rs7795660	T	0.62	0.344	-0.154	1.19E-07	0.350	-0.155	**4.56E-14**	0.326	-0.163	7.41E-04	0.347	-0.09	0.017	0.336	-0.025	0.643	0.350	-0.056	0.067	-0.122	**2.02E-21**	62	0.022
rs7795692	G	0.62	0.351	-0.154	1.12E-07	0.350	-0.155	**4.61E-14**	0.326	-0.163	7.46E-04	0.347	-0.09	0.017	0.336	-0.025	0.643	0.350	-0.056	0.067	-0.122	**2.02E-21**	62	0.022
rs11771945	G	0.62	0.344	-0.154	1.18E-07	0.351	-0.155	**3.88E-14**	0.327	-0.164	6.61E-04	0.348	-0.09	0.017	0.337	-0.026	0.634	0.350	-0.055	0.068	-0.122	**2.11E-21**	62	0.021
rs10480747	C	0.62	0.341	-0.154	1.27E-07	0.351	-0.155	**4.11E-14**	0.326	-0.164	6.86E-04	0.347	-0.09	0.017	0.336	-0.025	0.637	0.350	-0.055	0.068	-0.122	**2.20E-21**	63	0.020
rs7778938	C	0.62	0.343	-0.153	1.40E-07	0.350	-0.155	**4.63E-14**	0.326	-0.163	7.52E-04	0.347	-0.09	0.017	0.336	-0.025	0.644	0.350	-0.056	0.067	-0.122	**2.34E-21**	62	0.022
rs11765163	T	0.62	0.344	-0.153	1.19E-07	0.351	-0.155	**3.95E-14**	0.327	-0.164	6.70E-04	0.348	-0.09	0.017	0.337	-0.026	0.634	0.350	-0.055	0.068	-0.122	**2.44E-21**	62	0.021
rs2110281	A	0.56	0.351	-0.135	2.24E-06	0.365	-0.159	**2.27E-15**	0.297	-0.151	0.003	0.343	-0.071	0.057	0.332	-0.016	0.762	0.355	-0.066	0.028	-0.119	**3.15E-21**	62	0.022
rs10228519	G	0.50	0.488	-0.102	1.72E-04	0.486	-0.144	**1.11E-13**	0.463	-0.113	0.014	0.495	-0.071	0.046	0.481	-0.078	0.121	0.499	-0.066	0.022	-0.108	**1.20E-19**	31	0.204
rs6971407	T	0.45	0.491	0.088	0.001	0.487	0.141	**2.85E-13**	0.510	0.123	0.007	0.475	0.086	0.017	0.484	0.091	0.074	0.472	0.058	0.045	0.106	**7.50E-19**	30	0.213
rs6972481	C	0.45	0.492	0.088	0.001	0.514	0.141	**2.95E-13**	0.510	0.123	0.007	0.475	0.085	0.017	0.484	0.092	0.071	0.472	0.058	0.045	0.106	**7.82E-19**	30	0.211
**rs917727**	T	0.04	0.296	0.119	2.02E-04	0.273	0.085	1.77E-04	0.238	0.167	0.003	0.274	0.141	4.73E-04	0.263	0.113	0.061	0.269	0.143	1.64E-05	0.116	**1.90E-16**	0	0.586
rs1005400	A	0.28	0.468	-0.107	7.99E-05	0.462	-0.13	**2.36E-11**	0.426	-0.075	0.101	0.452	-0.06	0.089	0.439	-0.046	0.376	0.455	-0.062	0.032	-0.098	**3.41E-16**	28	0.227
rs917726	T	0.04	0.282	0.114	3.61E-04	0.273	0.085	1.78E-04	0.238	0.167	0.003	0.274	0.141	4.75E-04	0.263	0.113	0.061	0.269	0.143	1.66E-05	0.115	**3.90E-16**	0	0.592
rs1917114	A	0.28	0.470	-0.11	4.87E-05	0.462	-0.129	**2.58E-11**	0.426	-0.075	0.102	0.452	-0.059	0.093	0.440	-0.046	0.377	0.456	-0.06	0.040	-0.097	**4.09E-16**	29	0.216
rs7776725	*C*	*0.04*	*0.264*	*0.12*	*2.29E-04*	*0.462*	*0.086*	*1.91E-04*	*0.239*	*0.176*	*0.003*	*0.274*	*0.142*	*4.81E-04*	*0.263*	*0.114*	*0.060*	*0.269*	*0.145*	*1.71E-05*	*0.117*	***4.74E-16***	*0*	*0.567*
rs3801382	G	0.04	0.275	0.104	7.44E-04	0.462	0.083	1.56E-04	0.238	0.164	0.003	0.274	0.139	4.95E-04	0.263	0.111	0.062	0.269	0.142	1.71E-05	0.111	**8.14E-16**	0	0.549
rs718766	C	0.04	0.272	0.11	6.15E-04	0.462	0.085	1.82E-04	0.238	0.169	0.003	0.274	0.141	4.76E-04	0.263	0.113	0.061	0.269	0.144	1.69E-05	0.114	**8.78E-16**	0	0.578

Variant			Generation R			ALSPAC			GOOD			RS-III			RS-II			RS-I			Combined			
SNP	A1	R2*	Freq.	BETA**	P	Freq	BETA**	P	Freq	BETA**	P	Freq	BETA**	P	Freq	BETA**	P	Freq	BETA**	P	BETA**	P	I2	HetP
rs2908004	A	0.05	0.501	0.102	2.06E-04	0.462	0.072	3.09E-04	0.431	0.071	0.145	0.456	0.127	0.001	0.437	0.157	0.003	0.449	0.14	4.97E-06	0.100	**9.11E-16**	12	0.340
rs2536189	*G*	*0.05*	*0.498*	*0.099*	*2.72E-04*	*0.442*	*0.073*	*3.07E-04*	*0.431*	*0.071*	*0.145*	*0.456*	*0.127*	*0.001*	*0.437*	*0.157*	*0.003*	*0.449*	*0.14*	*5.01E-06*	*0.100*	***1.07E-15***	*10*	*0.355*
rs2536149	A	0.05	0.139	-0.256	**3.67E-10**	0.115	-0.151	9.49E-07	0.085	-0.196	0.017	0.102	-0.076	0.199	0.103	-0.114	0.188	0.110	-0.068	0.137	0.152	**3.30E-15**	58	0.036
rs3801387	G	0.04	0.274	0.092	0.003	0.272	0.083	1.51E-04	0.238	0.166	0.002	0.275	0.139	4.66E-04	0.263	0.11	0.065	0.270	0.138	2.67E-05	0.108	**4.68E-15**	0	0.530
rs2968349	C	0.63	0.457	-0.112	5.23E-05	0.468	-0.129	2.74E-11	0.374	-0.121	0.012	0.436	-0.051	0.149	0.428	-0.048	0.356	0.454	-0.03	0.295	-0.095	**5.20E-15**	55	0.048
rs2536148	T	0.05	0.153	-0.241	**2.49E-09**	0.111	-0.154	1.00E-06	0.083	-0.21	0.012	0.101	-0.071	0.237	0.102	-0.119	0.172	0.108	-0.06	0.198	-0.150	**1.27E-14**	56	0.044
rs4727924	T	0.06	0.467	0.117	6.03E-05	0.459	0.067	0.001	0.454	0.063	0.192	0.477	0.115	0.001	0.463	0.156	0.003	0.476	0.118	6.65E-05	0.096	**1.93E-14**	4	0.389
rs2536182	G	0.05	0.471	0.101	3.14E-04	0.454	0.065	9.08E-04	0.439	0.095	0.047	0.466	0.119	0.001	0.452	0.163	0.002	0.463	0.111	1.73E-04	0.094	**2.07E-14**	0	0.438
rs2536180	C	0.06	0.495	0.098	3.33E-04	0.463	0.063	0.001	0.456	0.061	0.193	0.478	0.114	0.001	0.465	0.153	0.003	0.477	0.116	6.68E-05	0.090	**6.63E-14**	5	0.382
rs2254595	C	0.06	0.504	0.096	3.85E-04	0.463	0.063	0.001	0.456	0.061	0.193	0.478	0.114	0.001	0.464	0.153	0.003	0.477	0.116	6.38E-05	0.089	**8.51E-14**	5	0.387
rs798903	C	0.32	0.498	0.107	2.04E-04	0.463	0.118	**2.67E-09**	0.528	0.147	0.001	0.510	0.04	0.266	0.517	0.001	0.989	0.504	0.06	0.038	0.092	**1.64E-13**	49	0.083
rs3757552	G	0	0.112	-0.176	6.49E-05	0.122	-0.125	3.04E-05	0.117	-0.198	0.006	0.106	-0.094	0.092	0.122	-0.212	0.005	0.118	-0.099	0.024	-0.136	**4.06E-13**	0	0.554
rs10953925	G	0.23	0.457	-0.096	5.43E-04	0.506	-0.116	**2.16E-09**	0.428	-0.092	0.043	0.481	-0.08	0.022	0.474	-0.017	0.744	0.495	-0.033	0.257	-0.087	**6.07E-13**	36	0.166
rs13246689	C	0.23	0.456	-0.096	5.63E-04	0.507	-0.116	**2.43E-09**	0.429	-0.091	0.046	0.482	-0.08	0.022	0.475	-0.017	0.744	0.495	-0.032	0.266	-0.086	**7.02E-13**	37	0.159
rs10953924	C	0.23	0.453	-0.094	7.36E-04	0.507	-0.116	**2.18E-09**	0.428	-0.092	0.043	0.481	-0.08	0.022	0.474	-0.017	0.744	0.495	-0.033	0.260	-0.086	**7.60E-13**	36	0.168
rs11766764	T	0.23	0.454	-0.094	6.92E-04	0.507	-0.116	**2.22E-09**	0.428	-0.092	0.044	0.481	-0.08	0.022	0.474	-0.017	0.744	0.495	-0.032	0.261	-0.086	**8.43E-13**	37	0.161
rs3779381	G	0.05	0.264	0.083	0.009	0.255	0.074	0.001	0.227	0.155	0.006	0.254	0.13	0.002	0.241	0.101	0.106	0.250	0.154	9.02E-06	0.103	**8.84E-13**	7	0.372
rs2707466	*T*	*0.06*	*0.485*	*0.096*	*5.04E-04*	*0.425*	*0.058*	*0.005*	*0.418*	*0.056*	*0.255*	*0.440*	*0.119*	*0.002*	*0.424*	*0.166*	*0.002*	*0.435*	*0.132*	*2.05E-05*	*0.092*	***9.98E-13***	*31*	*0.204*
rs13247600	C	0.03	0.064	-0.18	0.005	0.076	-0.186	2.14E-05	0.089	-0.158	0.100	0.082	-0.173	0.018	0.084	-0.181	0.117	0.079	-0.239	8.74E-05	-0.191	**1.02E-12**	0	0.974
rs12706314	G	0.53	0.451	0.089	0.001	0.443	0.116	**2.62E-09**	0.487	0.088	0.055	0.446	0.047	0.188	0.461	0.08	0.121	0.439	0.036	0.210	0.085	**1.48E-12**	25	0.247
rs798949	C	0.53	0.441	0.088	0.001	0.443	-0.116	**2.51E-09**	0.487	0.088	0.055	0.446	0.047	0.188	0.461	0.08	0.121	0.439	0.036	0.210	0.085	**1.65E-12**	25	0.248
rs2110280	C	0.22	0.459	-0.091	9.69E-04	0.515	-0.115	**3.31E-09**	0.440	-0.105	0.021	0.487	-0.072	0.040	0.486	-0.01	0.848	0.503	-0.032	0.267	-0.085	**1.96E-12**	40	0.137
rs6466766	C	0.23	0.453	-0.095	6.54E-04	0.506	-0.118	**2.49E-09**	0.429	-0.091	0.049	0.482	-0.08	0.022	0.475	-0.017	0.745	0.496	-0.032	0.275	-0.086	**2.85E-12**	38	0.156
rs10953932	G	0.55	0.464	-0.118	1.98E-05	0.460	-0.104	8.11E-08	0.424	-0.148	0.001	0.439	-0.049	0.167	0.427	-0.015	0.772	0.447	-0.019	0.515	-0.084	**3.87E-12**	59	0.032
rs12672898	A	0.55	0.464	-0.118	1.94E-05	0.460	-0.104	8.09E-08	0.424	-0.148	0.001	0.439	-0.049	0.166	0.427	-0.015	0.773	0.447	-0.019	0.518	-0.084	**3.87E-12**	59	0.032
rs1534017	C	0.55	0.465	-0.118	1.94E-05	0.460	-0.104	8.22E-08	0.424	-0.148	0.001	0.439	-0.049	0.167	0.427	-0.015	0.774	0.447	-0.019	0.508	-0.084	**3.87E-12**	59	0.032
rs6978080	A	0.55	0.464	-0.118	1.81E-05	0.460	-0.104	8.05E-08	0.424	-0.147	0.001	0.439	-0.049	0.166	0.427	-0.015	0.773	0.447	-0.019	0.528	-0.084	**4.03E-12**	59	0.032
rs1524506	T	0.55	0.457	-0.116	2.75E-05	0.460	-0.104	8.29E-08	0.424	-0.148	0.001	0.439	-0.049	0.167	0.427	-0.015	0.775	0.447	-0.02	0.507	-0.084	**4.35E-12**	58	0.036
rs10215148	A	0.43	0.439	0.085	0.002	0.442	0.117	**2.25E-09**	0.487	0.086	0.062	0.444	0.049	0.173	0.459	0.082	0.109	0.437	0.038	0.187	0.084	**5.24E-12**	19	0.288
rs10215475	C	0.43	0.439	0.085	0.002	0.442	0.117	**2.22E-09**	0.487	0.086	0.062	0.444	0.049	0.173	0.459	0.082	0.109	0.437	0.038	0.187	0.084	**5.24E-12**	19	0.288
rs1125447	A	0.43	0.439	0.085	0.002	0.442	0.117	**2.27E-09**	0.487	0.086	0.061	0.444	0.049	0.174	0.459	0.082	0.109	0.437	0.038	0.188	0.084	**5.24E-12**	19	0.288

Variant			Generation R			ALSPAC			GOOD			RS-III			RS-II			RS-I			Combined			
**SNP**	**A1**	**R2***	**Freq.**	**BETA****	**P**	**Freq**	**BETA****	**P**	**Freq**	**BETA****	**P**	**Freq**	**BETA****	**P**	**Freq**	**BETA****	**P**	**Freq**	**BETA****	**P**	**BETA****	**P**	**I2**	**HetP**
rs1357755	C	0.43	0.439	0.085	0.002	0.442	0.117	**2.19E-09**	0.487	0.086	0.062	0.444	0.049	0.172	0.459	0.082	0.108	0.437	0.038	0.186	0.084	**5.24E-12**	19	0.288
rs2402560	T	0.43	0.439	0.085	0.002	0.442	0.117	**2.32E-09**	0.487	0.086	0.061	0.444	0.048	0.174	0.459	0.082	0.109	0.437	0.038	0.188	0.084	**5.60E-12**	20	0.283
rs11531545	G	0.43	0.433	0.084	0.002	0.442	0.117	**2.21E-09**	0.487	0.086	0.062	0.444	0.049	0.173	0.459	0.082	0.108	0.437	0.038	0.186	0.084	**5.89E-12**	19	0.288
rs10251139	C	0.53	0.441	0.086	0.002	0.443	0.116	**2.79E-09**	0.486	0.088	0.057	0.446	0.047	0.186	0.461	0.079	0.122	0.439	0.036	0.210	0.084	**8.22E-12**	22	0.271
rs10235934	G	0.43	0.439	0.084	0.002	0.442	0.117	**2.30E-09**	0.487	0.086	0.061	0.444	0.048	0.174	0.459	0.082	0.109	0.437	0.038	0.188	0.084	**8.91E-12**	20	0.283
rs1524498	C	0.53	0.433	0.085	0.002	0.443	0.116	**2.73E-09**	0.486	0.088	0.056	0.446	0.047	0.188	0.461	0.08	0.121	0.439	0.036	0.210	0.083	**8.94E-12**	22	0.272
rs1558541	C	0.53	0.433	0.085	0.002	0.443	0.116	**2.59E-09**	0.487	0.088	0.056	0.446	0.047	0.188	0.461	0.08	0.121	0.439	0.036	0.210	0.083	**8.94E-12**	22	0.272
rs1949803	G	0.58	0.509	-0.108	1.30E-04	0.460	-0.11	**2.85E-08**	0.420	-0.152	0.001	0.441	-0.047	0.197	0.429	-0.019	0.713	0.450	-0.021	0.491	-0.085	**9.52E-12**	57	0.043
rs10246521	T	0.53	0.439	0.085	0.002	0.442	0.115	**4.12E-09**	0.487	0.088	0.057	0.446	0.047	0.183	0.460	0.081	0.115	0.438	0.037	0.205	0.083	**9.67E-12**	18	0.297
rs1112208	G	0.53	0.433	0.085	0.002	0.442	0.115	**4.12E-09**	0.487	0.088	0.057	0.446	0.047	0.183	0.460	0.081	0.116	0.438	0.037	0.206	0.083	**9.67E-12**	18	0.297
rs2177578	A	0.53	0.433	0.085	0.002	0.442	0.115	**3.83E-09**	0.487	0.088	0.056	0.446	0.047	0.185	0.461	0.08	0.118	0.439	0.037	0.206	0.083	**1.00E-11**	18	0.297
rs10248011	A	0.53	0.433	0.085	0.002	0.443	0.115	**3.54E-09**	0.487	0.088	0.056	0.446	0.047	0.186	0.461	0.08	0.118	0.439	0.036	0.207	0.083	**1.11E-11**	20	0.287
rs10216123	G	0.38	0.421	0.072	0.010	0.414	0.116	**3.56E-09**	0.461	0.101	0.028	0.414	0.066	0.071	0.423	0.092	0.075	0.408	0.03	0.308	0.084	**1.17E-11**	25	0.250
rs10259383	T	0.53	0.441	0.087	0.002	0.443	0.115	**3.38E-09**	0.487	0.088	0.056	0.446	0.047	0.186	0.461	0.08	0.119	0.439	0.036	0.207	0.083	**1.27E-11**	20	0.285
rs17284918	G	0.55	0.509	-0.103	2.12E-04	0.460	-0.104	7.79E-08	0.424	-0.148	0.001	0.439	-0.049	0.167	0.428	-0.018	0.737	0.447	-0.019	0.526	-0.081	**1.77E-11**	55	0.051
rs6979948	T	0.49	0.435	-0.129	4.78E-06	0.436	-0.101	2.81E-07	0.404	-0.124	0.008	0.420	-0.053	0.146	0.408	-0.019	0.708	0.426	-0.018	0.550	-0.083	**2.10E-11**	57	0.042
rs1534019	T	0.49	0.440	-0.125	8.28E-06	0.437	-0.102	2.05E-07	0.405	-0.127	0.006	0.420	-0.053	0.146	0.409	-0.021	0.692	0.427	-0.016	0.578	-0.082	**2.32E-11**	57	0.042
rs7792071	G	0.49	0.446	-0.126	7.08E-06	0.436	-0.101	2.36E-07	0.405	-0.126	0.007	0.420	-0.053	0.146	0.408	-0.02	0.699	0.427	-0.017	0.566	-0.082	**2.50E-11**	56	0.044
rs1917112	G	0.36	0.434	0.095	5.21E-04	0.435	0.112	**1.03E-08**	0.497	0.061	0.189	0.455	0.049	0.165	0.465	0.079	0.126	0.446	0.032	0.272	0.081	**2.51E-11**	24	0.256
rs1534014	C	0.49	0.440	-0.125	8.83E-06	0.436	-0.101	2.19E-07	0.405	-0.127	0.006	0.420	-0.053	0.146	0.408	-0.02	0.696	0.427	-0.017	0.571	-0.082	**2.68E-11**	56	0.046
rs7808120	A	0.49	0.440	-0.125	8.67E-06	0.437	-0.101	2.17E-07	0.405	-0.127	0.006	0.420	-0.053	0.146	0.408	-0.02	0.696	0.427	-0.017	0.573	-0.082	**2.68E-11**	56	0.046
rs12706326	A	0.10	0.044	-0.213	0.005	0.051	-0.245	3.80E-06	0.053	-0.102	0.398	0.054	-0.18	0.047	0.057	-0.238	0.084	0.055	-0.189	0.003	-0.208	**2.75E-11**	0	0.914
rs17357115	C	0.55	0.510	-0.1	2.83E-04	0.460	-0.104	8.06E-08	0.424	-0.147	0.001	0.439	-0.049	0.166	0.427	-0.015	0.773	0.447	-0.019	0.522	-0.081	**2.76E-11**	54	0.052
rs7805374	G	0.49	0.440	-0.125	9.10E-06	0.436	-0.101	2.30E-07	0.405	-0.126	0.007	0.420	-0.053	0.146	0.408	-0.02	0.698	0.427	-0.017	0.568	-0.082	**2.78E-11**	56	0.046
rs17143161	G	0.36	0.443	0.09	9.79E-04	0.434	0.111	**1.16E-08**	0.496	0.062	0.181	0.455	0.051	0.146	0.467	0.08	0.118	0.448	0.036	0.221	0.081	**3.00E-11**	12	0.339
rs6466774	C	0.49	0.439	-0.124	9.61E-06	0.436	-0.101	2.59E-07	0.404	-0.126	0.007	0.420	-0.053	0.146	0.408	-0.02	0.705	0.426	-0.017	0.556	-0.082	**3.57E-11**	55	0.049
rs7806875	T	0.49	0.440	-0.124	1.02E-05	0.436	-0.101	2.80E-07	0.404	-0.124	0.008	0.420	-0.053	0.146	0.408	-0.019	0.707	0.426	-0.018	0.551	-0.082	**3.59E-11**	54	0.053
rs10274486	C	0.38	0.411	0.068	0.015	0.415	0.108	3.76E-08	0.460	0.102	0.027	0.415	0.065	0.075	0.424	0.09	0.082	0.407	0.027	0.358	0.079	**1.39E-10**	16	0.314
rs6942652	C	0.47	0.434	-0.114	5.10E-05	0.435	-0.099	4.26E-07	0.403	-0.123	0.008	0.419	-0.052	0.146	0.408	-0.02	0.704	0.426	-0.018	0.537	-0.079	**1.66E-10**	49	0.084
rs10953933	T	0.47	0.432	-0.114	5.01E-05	0.435	-0.099	4.44E-07	0.403	-0.122	0.009	0.419	-0.052	0.146	0.408	-0.02	0.705	0.426	-0.018	0.534	-0.079	**1.72E-10**	48	0.085
rs11509199	T	0.47	0.432	-0.114	4.96E-05	0.435	-0.099	4.41E-07	0.403	-0.122	0.009	0.419	-0.052	0.146	0.408	-0.02	0.705	0.426	-0.018	0.535	-0.079	**1.72E-10**	48	0.085

Variant			Generation R			ALSPAC			GOOD			RS-III			RS-II			RS-I			Combined			
SNP	A1	R2*	Freq.	BETA**	P	Freq	BETA**	P	Freq	BETA**	P	Freq	BETA**	P	Freq	BETA**	P	Freq	BETA**	P	BETA**	P	I2	HetP
rs12706333	T	0.47	0.434	-0.114	5.29E-05	0.435	-0.099	4.64E-07	0.403	-0.122	0.009	0.419	-0.052	0.146	0.408	-0.02	0.707	0.426	-0.018	0.530	-0.079	**1.72E-10**	48	0.085
rs10266975	C	0.47	0.434	-0.114	5.35E-05	0.435	-0.099	4.71E-07	0.403	-0.122	0.009	0.419	-0.052	0.146	0.408	-0.019	0.708	0.426	-0.018	0.529	-0.079	**1.77E-10**	49	0.084
rs17536644	G	0.34	0.419	0.093	0.001	0.432	0.111	**1.77E-08**	0.499	0.054	0.248	0.455	0.045	0.206	0.465	0.081	0.115	0.444	0.029	0.316	0.079	**1.87E-10**	28	0.224
rs6948725	C	0.47	0.440	-0.114	4.54E-05	0.435	-0.098	4.92E-07	0.403	-0.121	0.009	0.419	-0.052	0.146	0.408	-0.019	0.710	0.426	-0.019	0.525	-0.079	**2.04E-10**	47	0.093
rs10225276	G	0.47	0.434	-0.113	5.40E-05	0.435	-0.098	4.89E-07	0.403	-0.122	0.009	0.419	-0.052	0.146	0.408	-0.019	0.709	0.426	-0.019	0.526	-0.078	**2.18E-10**	47	0.095
rs12539571	C	0.47	0.434	-0.113	5.91E-05	0.435	-0.098	5.29E-07	0.402	-0.121	0.009	0.419	-0.052	0.146	0.407	-0.019	0.713	0.426	-0.019	0.522	-0.078	**2.26E-10**	47	0.096
rs10251901	G	0.38	0.413	0.07	0.013	0.413	0.107	4.31E-08	0.460	0.101	0.028	0.414	0.064	0.078	0.423	0.086	0.097	0.406	0.024	0.412	0.078	**2.30E-10**	18	0.299
rs6970383	C	0.47	0.435	-0.114	5.17E-05	0.438	-0.096	8.00E-07	0.404	-0.118	0.011	0.419	-0.052	0.150	0.408	-0.019	0.720	0.426	-0.019	0.522	-0.078	**3.39E-10**	45	0.104
rs4731007	G	0.47	0.437	-0.114	4.74E-05	0.438	-0.096	8.69E-07	0.404	-0.117	0.012	0.419	-0.051	0.154	0.409	-0.018	0.726	0.427	-0.019	0.523	-0.077	**3.40E-10**	46	0.101
rs6947934	C	0.47	0.437	-0.114	4.82E-05	0.438	-0.096	8.57E-07	0.404	-0.117	0.012	0.419	-0.051	0.153	0.408	-0.018	0.724	0.427	-0.019	0.523	-0.077	**3.40E-10**	46	0.101
rs3801385	C	0.00	0.101	-0.172	1.96E-04	0.105	-0.112	4.25E-04	0.101	-0.181	0.017	0.087	-0.088	0.151	0.100	-0.149	0.070	0.098	-0.101	0.035	-0.126	**3.86E-10**	0	0.784
rs11770502	G	0.41	0.453	-0.113	9.01E-05	0.450	-0.1	5.42E-07	0.423	-0.127	0.008	0.437	-0.042	0.258	0.426	-0.019	0.720	0.447	-0.017	0.575	-0.078	**4.48E-10**	50	0.074
rs12706321	G	0.07	0.034	-0.231	0.007	0.042	-0.251	3.50E-05	0.042	-0.101	0.474	0.045	-0.189	0.066	0.045	-0.254	0.126	0.047	-0.221	0.003	-0.223	**5.93E-10**	0	0.953
rs13226812	G	0.07	0.035	-0.234	0.007	0.042	-0.251	4.23E-05	0.042	-0.106	0.462	0.046	-0.175	0.095	0.046	-0.214	0.207	0.048	-0.223	0.004	-0.221	**1.30E-09**	0	0.954
rs10261386	T	0.27	0.384	0.074	0.010	0.390	0.102	3.72E-07	0.455	0.069	0.132	0.407	0.063	0.080	0.413	0.063	0.227	0.399	0.022	0.466	0.073	**4.14E-09**	6	0.378
rs17143147	T	0.39	0.403	0.076	0.010	0.443	0.102	1.53E-07	0.521	0.058	0.211	0.469	0.044	0.210	0.476	0.062	0.222	0.457	0.011	0.691	0.069	**1.01E-08**	35	0.176
rs6962509	A	0.14	0.445	0.076	0.007	0.424	0.099	5.98E-07	0.479	0.064	0.155	0.443	0.061	0.085	0.449	0.022	0.670	0.435	0.025	0.393	0.070	**1.28E-08**	9	0.359
rs10275526	C	0.14	0.440	0.072	0.010	0.424	0.099	5.35E-07	0.479	0.065	0.150	0.444	0.061	0.084	0.449	0.022	0.670	0.435	0.025	0.384	0.069	**1.78E-08**	8	0.365
rs2192291	T	0.14	0.427	0.073	0.010	0.424	0.099	6.38E-07	0.478	0.063	0.161	0.443	0.061	0.085	0.448	0.022	0.670	0.435	0.024	0.400	0.069	**1.90E-08**	10	0.351
rs12666340	C	0.14	0.443	0.071	0.012	0.424	0.099	5.49E-07	0.479	0.065	0.150	0.444	0.061	0.084	0.449	0.022	0.670	0.435	0.025	0.384	0.069	**1.95E-08**	8	0.365
rs12666345	G	0.14	0.442	0.07	0.012	0.424	0.099	5.38E-07	0.479	0.065	0.150	0.444	0.061	0.084	0.449	0.022	0.670	0.435	0.025	0.384	0.069	**2.13E-08**	8	0.366
rs12672958	C	0.14	0.442	0.07	0.012	0.424	0.099	5.57E-07	0.479	0.065	0.150	0.444	0.061	0.084	0.449	0.022	0.670	0.435	0.025	0.384	0.069	**2.13E-08**	8	0.366
rs11977285	A	0.14	0.442	0.07	0.012	0.424	0.099	6.04E-07	0.479	0.064	0.157	0.443	0.061	0.085	0.448	0.022	0.670	0.435	0.025	0.395	0.069	**2.21E-08**	8	0.365
rs2402555	T	0.14	0.442	0.07	0.012	0.424	0.099	6.19E-07	0.478	0.064	0.158	0.443	0.061	0.085	0.448	0.022	0.670	0.435	0.025	0.396	0.069	**2.21E-08**	8	0.365
rs798905	C	0.06	0.346	-0.124	3.66E-05	0.260	-0.06	7.04E-03	0.232	-0.093	0.098	0.244	-0.047	0.260	0.250	-0.146	0.018	0.252	-0.047	0.157	-0.076	**3.51E-08**	12	0.336
rs798913	A	0.02	0.073	0.11	0.056	0.081	0.156	1.66E-05	0.075	0.074	0.407	0.068	0.132	0.060	0.068	-0.046	0.672	0.071	0.157	0.009	0.130	**4.81E-08**	0	0.573
rs2691034	G	0.07	0.329	-0.123	7.59E-05	0.266	-0.063	0.005	0.237	-0.09	0.107	0.248	-0.044	0.293	0.255	-0.144	0.020	0.256	-0.046	0.172	-0.076	**5.38E-08**	5	0.384

Shaded rs917727 top-hit for GW- association with TB-BMD and rs4609139 top-hit of the secondary signal in TB-BMD *Correlation coefficients with rs7801723 based on HapMap release22 CEU population. **Effect estimates expressed as standardized adjusted SD per copy of allele (A1). Underline: rs7776725 Top-hit wrist fracture GWAS, rs2536189 Top-hit forearm BMD GWAS, rs2707466 Top-hit for Cortical thickness in Zheng et al (accompanying submission).

Supplementary Table 6. *Fam3c* KO mouse data for each knockout strategy. Gene trap and two types of homologous recombination: 1 and 2 (HR #1, HR #2)

	KO	Male WT	Male KO	Female WT	Female KO
**Number of Mice**	Gene Trap	2	3	2	3
	HR #1	2	4	2	4
	HR #2	2	4	2	4
**Body Weight (grams)**	Gene Trap	33.9 ± 1.8	35.9 ± 1.4	20.4 ± 2.5	24.7 ± 1.5
	HR #1	27.1 ± 3.4	29.5 ± 2.6	22.7 ± 1.7	23.2 ± 1.3
	HR #2	33.0 ± 2.7	26.6 ± 1.4	20.6 ± 1.2	23.9 ± 0.7
**LBM (grams)**	Gene Trap	25.2 ± 1.2	26.2 ± 1.1	14.9 ± 1.7	17.2 ± 0.5
	HR #1	21.0 ± 1.9	22.9 ± 1.7	17.3 ± 0.8	17.0 ± 0.9
	HR #2	24.2 ± 1.6	20.7 ± 1.1	16.4 ± 1.1	18.4 ± 0.4
**Body Fat (percent)**	Gene Trap	21.4 ± 1.3	24.1 ± 1.3	20.9 ± 2.5	26.6 ± 5.6
	HR #1	18.3 ± 3.8	19.0 ± 1.9	19.7 ± 2.4	22.5 ± 3.5
	HR #2	26.0 ± 2.3	21.5 ± 1.1	17.2 ± 0.0	20.4 ± 2.8
**Body aBMD (mg/cm2)**	Gene Trap	53.6 ± 2.5	53.6 ± 0.8	48.3 ± 0.7	49.5 ± 0.8
	HR #1	51.4 ± 3.9	51.8 ± 1.9	47.3 ± 1.3	47.0 ± 1.3
	HR #2	51.4 ± 0.1	50.5 ± 1.4	47.6 ± 0.9	50.9 ± 0.9
**Body Bone Area (cm2)**	Gene Trap	10.0 ± 0.2	10.0 ± 0.4	9.4 ± 0.2	9.0 ± 0.7
	HR #1	9.5 ± 0.3	9.4 ± 0.2	8.5 ± 0.1	8.8 ± 0.3
	HR #2	8.7 ± 0.0	8.9 ± 0.4	8.8 ± 0.6	8.8 ± 0.2
**Body BMC (mg)**	Gene Trap	533 ± 35	537 ± 24	452 ± 18	447 ± 40
	HR #1	485 ± 19	486 ± 21	404 ± 17	414 ± 25
	HR #2	446 ± 3	450 ± 28	421 ± 35	450 ± 17
**Femur aBMD (mg/cm2)**	Gene Trap	84.8 ± 4.6	85.1 ± 4.5	64.9 ± 1.7	72.8 ± 2.4
	HR #1	76.8 ± 3.2	78.9 ± 4.1	71.6 ± 0.1	69.8 ± 0.9
	HR #2	74.8 ± 0.3	80.1 ± 3.4	72.7 ± 2.0	77.2 ± 1.1
**Femur Bone Area (cm2)**	Gene Trap	0.40 ± 0.01	0.40 ± 0.01	0.37 ± 0.05	0.36 ± 0.02
	HR #1	0.38 ± 0.03	0.37 ± 0.01	0.33 ± 0.02	0.33 ± 0.01
	HR #2	0.39 ± 0.01	0.33 ± 0.02	0.33 ± 0.01	0.36 ± 0.01
**Femur BMC (mg)**	Gene Trap	33.9 ± 2.8	34.3 ± 1.8	24.0 ± 2.8	26.3 ± 1.1
	HR #1	29.1 ± 3.3	29.0 ± 2.3	23.5 ± 1.7	22.9 ± 0.9
	HR #2	29.1 ± 1.0	26.4 ± 2.7	23.9 ± 1.5	27.3 ± 0.7
**Spine aBMD (mg/ cm2)**	Gene Trap	68.5 ± 1.3	62.8 ± 0.9	63.6 ± 1.6	63.6 ± 0.4
	HR #1	58.5 ± 5.6	62.7 ± 1.9	54.8 ± 1.9	52.8 ± 3.6
	HR #2	56.2 ± 4.8	59.5 ± 2.7	53.8 ± 4.0	65.0 ± 3.6
**Spine BMC (mg)**	Gene Trap	31.4 ± 0.8	29.6 ± 0.8	28.4 ± 0.4	26.8 ± 1.2
	HR #1	26.6 ± 3.2	27.4 ± 0.6	23.6 ± 0.6	21.4 ± 0.9
	HR #2	22.9 ± 1.6	26.2 ± 1.0	23.7 ± 1.1	29.0 ± 2.0

Results provided as [mean +/- SEM]

Supplementary Table 7. SNPs showing association with TB-BMD in the discovery cohort, overall and by ethnic clustering

					All Generation R participants													
					Combined Generation R (n=2,660)			Generation R CEU (n=1,511)			Generation R Non-CEU							
		CEU	YRI	JPT/CHB							Overall (n=1,149)			CLUSTER 1 (n=263)		CLUSTER 2 (n=866)	
SNP	A1	R2*	R2*	R2*	Freq.	BETA**	P	Freq	BETA**	P	Freq	BETA**	P	BETA**	P	BETA**	P
**rs917727**	T	1	1	1	0.296	0.21	**4.11E-11**	0.263	0.233	1.30E-07	0.32	0.183	9.19E-05	0.214	0.03	0.173	0.001
rs2908004	A	0.55	0	0.563	0.501	0.16	**1.23E-08**	0.457	0.178	1.59E-06	0.526	0.131	0.001	0.156	0.094	0.127	0.009
rs917726	T	1	0.74	1	0.282	0.208	**6.42E-11**	0.256	0.231	1.34E-07	0.3	0.178	1.32E-04	0.149	0.123	0.182	0.001
rs718766	C	1	0.51	1	0.272	0.208	**8.63E-11**	0.253	0.232	1.17E-07	0.283	0.175	2.05E-04	0.084	0.397	0.189	0.001
rs3801382	G	1	0.51	0.778	0.275	0.199	**1.04E-10**	0.256	0.224	1.16E-07	0.287	0.165	2.64E-04	0.072	0.448	0.182	0
rs7776725	*C*	*1*	*0.38*	*1*	*0.264*	*0.214*	***4.58E-11***	*0.251*	*0.233*	*1.46E-07*	*0.27*	*0.188*	*1.02E-04*	*0.13*	*0.204*	*0.19*	*0.001*
rs2536189	*G*	*0.55*	*0.01*	*0.563*	*0.498*	*0.153*	***2.23E-08***	*0.455*	*0.177*	*1.71E-06*	*0.521*	*0.126*	*0.002*	*0.15*	*0.107*	*0.123*	*0.011*
rs3801387	G	1	0.32	0.778	0.274	0.197	**1.61E-10**	0.255	0.223	1.24E-07	0.287	0.162	3.25E-04	0.004	0.968	0.187	0
rs4727924	T	0.513	0.25	0.641	0.467	0.172	**3.88E-09**	0.444	0.19	1.11E-06	0.474	0.155	4.84E-04	0.242	0.011	0.129	0.012
rs2536182	G	0.531	0	0.778	0.471	0.156	**2.69E-08**	0.443	0.186	7.23E-07	0.484	0.117	0.006	0.123	0.194	0.112	0.024
rs2536180	C	0.513	0.005	0.563	0.495	0.143	1.57E-07	0.464	0.172	2.70E-06	0.509	0.11	0.008	0.092	0.323	0.111	0.02
rs2707466	*T*	*0.511*	*0.01*	*0.563*	*0.485*	*0.152*	***3.55E-08***	*0.443*	*0.181*	*1.36E-06*	*0.51*	*0.118*	*0.004*	*0.139*	*0.134*	*0.116*	*0.018*
rs2254595	C	0.513	0.019	0.641	0.504	0.142	1.20E-07	0.469	0.166	4.90E-06	0.521	0.117	0.004	0.132	0.144	0.111	0.019
rs3779381	G	0.868	0.323	0.778	0.264	0.18	**1.57E-08**	0.241	0.197	7.31E-06	0.279	0.156	7.53E-04	-0.007	0.944	0.184	0.001
rs2536150	C	0.068	0.005	0	0.215	-0.136	6.22E-05	0.185	-0.166	5.08E-04	0.231	-0.114	0.02	-0.029	0.759	-0.125	0.03
rs2952559	C	0.055	0.001	0	0.265	-0.133	6.34E-05	0.211	-0.191	8.59E-05	0.306	-0.096	0.04	-0.118	0.2	-0.094	0.095
rs13247600	C	0.039	Monomorphic	Monomorphic	0.064	-0.219	7.07E-04	0.084	-0.189	0.013	0.043	-0.347	0.005	-0.471	0.302	-0.3	0.02
rs2707520	C	0.249	Monomorphic	0.053	0.444	-0.091	0.001	0.505	-0.093	0.013	0.386	-0.096	0.026	-0.114	0.275	-0.084	0.084
rs17509082	T	0.386	0.005	0.22	0.173	0.119	0.001	0.193	0.167	4.47E-04	0.171	0.048	0.386	0.074	0.547	0.018	0.767
rs2908007	A	0.37	0.028	0.083	0.492	-0.1	4.12E-04	0.57	-0.067	0.092	0.434	-0.133	1.77E-03	-0.151	0.137	-0.139	0.004

Bolded rs917727 top-hit * Correlation coefficients with rs917727 based on HapMap release22 Three different panel populations. **Effect estimates expressed as standardized adjusted SD per copy of allele (A1). Underline: rs7776725 Top-hit wrist fracture GWAS, rs2536189 Top-hit forearm BMD GWAS, rs2707466 Top-hit for Cortical thickness all reported in Zheng et al (accompanying submission).

Supplementary Table 8. Information on genotyping methods, quality control of SNPs, imputation, and statistical analysis for genome-wide association study cohorts

Cohort	Genotyping						Imputation			Association analyses			
	Platform	Genotype calling algorithm	Inclusion criteria			SNPs that met QC criteria	Imputation software	Inclusion criteria		Analyses software	No. analyzed SNPs	men	women
			MAF	Call rate	p for HWE			MAF	Imputation quality				
GENERATION R	Illumina HumanHap 550K Quad	Genome Studio	≥ 1%	≥ 98%	> 10-6	469,644	MACH/minimac	≥1%	MACH R2 ≥ 0.3	MACH2QTL via GRIMP	3,021,329	1,313	1,347
ALSPAC	Illumina HumanHap 550K Quad	Genome Studio	≥ 1%	≥ 97%	>5x10-7	464,311	MACH	≥1%	MACH R2 ≥ 0.3	MACH2QTL	2,543,887	2,647	2,787
GOOD	Illumina / HumanHap 610 Quad	Beadstudio Genecall	≥ 1%	≥ 97.5%	> 10-6	521,160	MACH	≥1%	MACH R2 ≥ 0.3	MACH2QTL via GRIMP	2,543,887	938	0
RS-III	Illumina / HumanHap 610 QUAD	Genome Studio	≥ 1%	≥ 97.5%	> 10-6	514,073	MACH	≥1%	MACH R2 ≥ 0.3	MACH2QTL via GRIMP	2,543,887	680	914
RS-II	Illumina / HumanHap 550 V.3 DUO; Illumina / HumanHap 610 QUAD	Genome Studio	≥ 1%	≥ 97.5%	> 10-6	466,389	MACH	≥1%	MACH R2 ≥ 0.3	MACH2QTL via GRIMP	2,543,887	345	405
RS-III	Illumina / HumanHap 610 QUAD	Genome Studio	≥ 1%	≥ 97.5%	> 10-6	514,073	MACH	≥1%	MACH R2 ≥ 0.3	MACH2QTL via GRIMP	2,543,887	1000	594
RS-I	Illumina / HumanHap 550K V.3 ADHumanHap 550 V.3 DUO;	Beadstudio Genecall	≥ 1%	≥ 97.5%	> 10-6	512,349	MACH	≥1%	MACH R2 ≥ 0.3	MACH2QTL via GRIMP	2,448,227	1,051	1,385

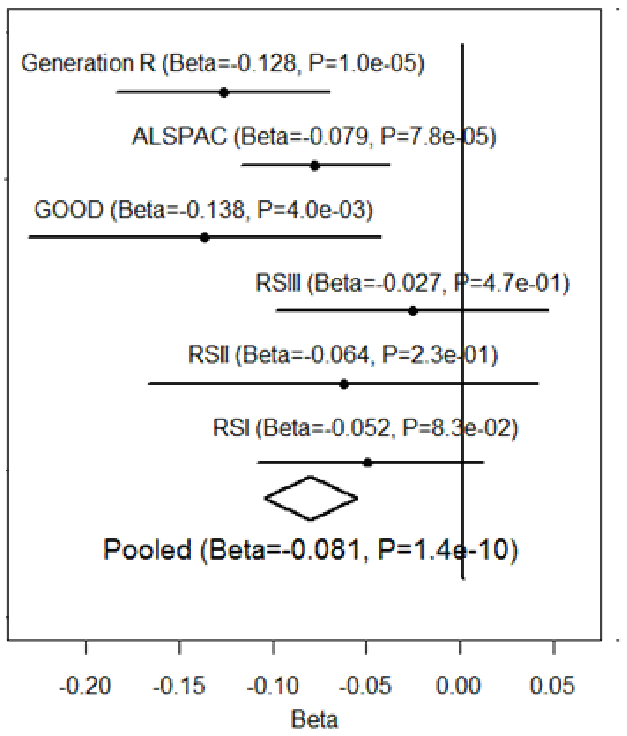

Supplementary Figure 1. Forest plot for the genome wide association of the rs4609139 with TB-BMD after conditioning on rs3801382, age, gender and weight. The results are reported per copy of the T-allele (MAF=0.328-0.356)

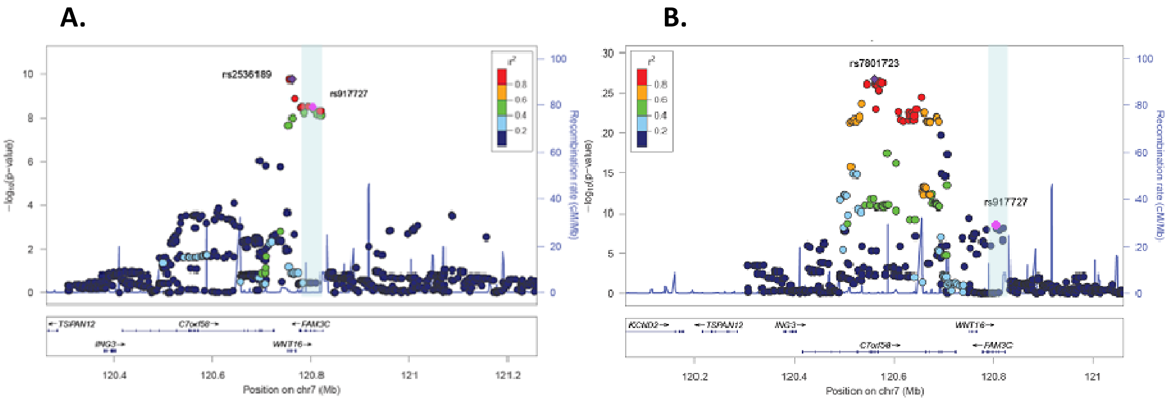

Supplementary Figure 2. A: SNP association plot for adults skull-BMD-associated region of Chromosome 7q31. **B**: SNP association plot for children skull-BMD-associated region of Chromosome 7q31. Genetic coordinates are as per Hapmap phase II-CEU.

A. **B.**

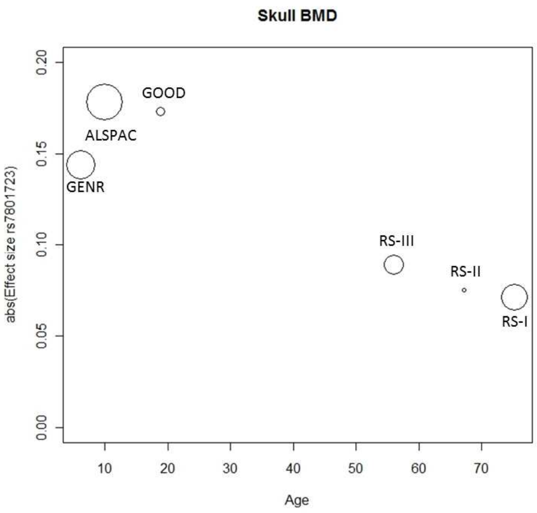

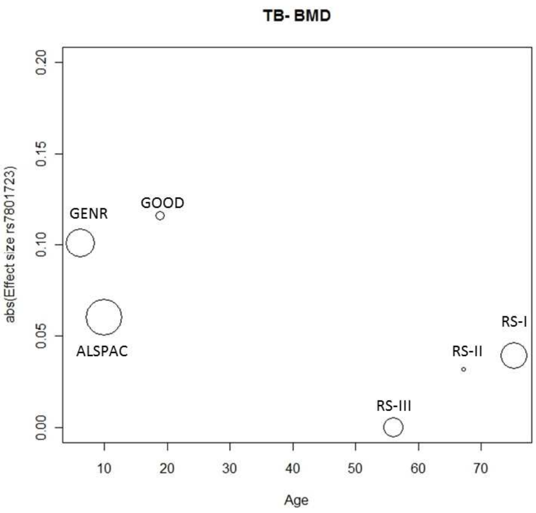

Supplementary Figure 3. Sample size weighted scatter plot of the absolute effect size versus the mean age of the studies for rs780123 in relation to **A.** skull and **B.** total body (right) BMD.
