# Supplementary material for: Meta-Analysis of Genome-Wide Scans for Total Body BMD in Children and Adults Reveals Allelic Heterogeneity and Age-Specific Effects at the WNT16 Locus
Source: PLoS Genet. 2012 Jul 5;8(7):e1002718. doi: 10.1371/journal.pgen.1002718 (PMC3390371; doi:10.1371/journal.pgen.1002718)
Supplement: Table S3 — SNPs showing GWS association with TB-BMD after conditioning by rs3801382. (PDF) [file pgen.1002718.s006.pdf]

			DISCOVERY			REPLICATION															COMBINED			
VARIANT			Generation R			ALSPAC			GOOD			RS-III			RS-II			RS-I						
			n= 2,660			n= 5,334			n= 938			n= 1,594			n= 750			n= 2,436			n=13,712			
SNP	A1	R2*	Freq.	BETA**	P	Freq	BETA**	P	Freq	BETA**	P	Freq	BETA**	P	Freq	BETA**	P	Freq	BETA**	P	BETA**	P	I2	HetP
**rs4609139**	T	1.00	0.355	-0.128	8.81E-06	0.356	-0.079	9.72E-05	0.328	-0.138	0.004	0.351	-0.027	0.472	0.341	-0.064	0.225	0.354	-0.052	0.081	-0.081	1.42E-10	31	0.205
rs4731006	T	1.00	0.346	-0.123	1.89E-05	0.356	-0.079	9.80E-05	0.327	-0.139	0.004	0.350	-0.027	0.465	0.340	-0.065	0.221	0.354	-0.053	0.080	-0.080	1.99E-10	25	0.248
rs6967129	G	1.00	0.346	-0.122	2.13E-05	0.353	-0.078	1.15E-04	0.327	-0.142	0.003	0.350	-0.028	0.452	0.340	-0.067	0.211	0.353	-0.053	0.076	-0.080	2.14E-10	25	0.250
rs12706334	G	1.00	0.346	-0.122	2.17E-05	0.353	-0.078	1.12E-04	0.326	-0.142	0.003	0.350	-0.028	0.454	0.340	-0.066	0.214	0.353	-0.053	0.077	-0.080	2.21E-10	25	0.250
rs798943	A	0.56	0.384	-0.117	2.32E-05	0.386	-0.080	6.02E-05	0.333	-0.139	0.004	0.383	-0.014	0.699	0.367	-0.066	0.206	0.393	-0.055	0.061	-0.078	2.59E-10	31	0.204
**rs7801723**	T	0.56	0.374	-0.117	2.39E-05	0.386	-0.080	6.10E-05	0.333	-0.140	0.004	0.383	-0.014	0.698	0.367	-0.066	0.204	0.393	-0.055	0.060	-0.078	2.72E-10	33	0.189
rs13232048	T	0.56	0.378	-0.117	2.59E-05	0.386	-0.080	6.32E-05	0.333	-0.140	0.004	0.383	-0.014	0.698	0.367	-0.066	0.204	0.393	-0.055	0.060	-0.078	2.72E-10	33	0.189
rs12706318	G	0.56	0.378	-0.117	2.59E-05	0.386	-0.080	6.25E-05	0.333	-0.140	0.004	0.383	-0.014	0.698	0.367	-0.066	0.204	0.393	-0.055	0.060	-0.078	2.72E-10	33	0.189
rs6950680	G	0.56	0.374	-0.118	2.14E-05	0.385	-0.079	6.97E-05	0.333	-0.140	0.004	0.383	-0.013	0.715	0.367	-0.067	0.203	0.393	-0.055	0.061	-0.078	3.10E-10	35	0.177
rs872007	T	0.56	0.379	-0.117	2.69E-05	0.385	-0.079	6.78E-05	0.333	-0.140	0.004	0.383	-0.014	0.699	0.367	-0.067	0.203	0.393	-0.055	0.060	-0.078	3.23E-10	33	0.190
rs6952113	A	0.56	0.379	-0.117	2.67E-05	0.385	-0.079	6.49E-05	0.333	-0.140	0.004	0.383	-0.014	0.699	0.367	-0.066	0.204	0.393	-0.055	0.060	-0.078	3.32E-10	33	0.189
rs13245690	G	0.56	0.376	-0.117	2.84E-05	0.387	-0.080	5.56E-05	0.337	-0.137	0.005	0.385	-0.015	0.689	0.370	-0.064	0.220	0.396	-0.053	0.070	-0.078	3.37E-10	30	0.208
rs10261671	T	0.46	0.388	-0.115	3.66E-05	0.386	-0.080	5.82E-05	0.334	-0.139	0.004	0.383	-0.012	0.734	0.367	-0.067	0.201	0.393	-0.054	0.069	-0.078	4.19E-10	31	0.204
rs10275439	A	0.53	0.386	-0.116	2.58E-05	0.383	-0.079	8.07E-05	0.330	-0.141	0.004	0.380	-0.012	0.750	0.362	-0.062	0.234	0.390	-0.055	0.063	-0.078	4.57E-10	33	0.191
rs10500083	C	0.93	0.351	-0.122	2.41E-05	0.350	-0.075	2.53E-04	0.326	-0.141	0.003	0.347	-0.025	0.511	0.336	-0.067	0.209	0.350	-0.053	0.078	-0.078	4.87E-10	27	0.233
rs2272196	A	0.93	0.351	-0.121	2.45E-05	0.350	-0.075	2.45E-04	0.326	-0.142	0.003	0.347	-0.025	0.512	0.336	-0.067	0.211	0.350	-0.053	0.079	-0.078	5.17E-10	26	0.237
rs7795692	G	0.93	0.351	-0.121	2.47E-05	0.350	-0.075	2.40E-04	0.326	-0.142	0.003	0.347	-0.025	0.513	0.336	-0.066	0.213	0.350	-0.053	0.079	-0.078	5.32E-10	26	0.236
rs10480747	C	0.93	0.341	-0.120	3.46E-05	0.351	-0.075	2.60E-04	0.326	-0.141	0.004	0.347	-0.025	0.510	0.336	-0.067	0.207	0.350	-0.053	0.077	-0.078	5.89E-10	25	0.250
rs7795660	T	0.93	0.344	-0.119	3.46E-05	0.350	-0.075	2.42E-04	0.326	-0.142	0.003	0.347	-0.025	0.513	0.336	-0.067	0.212	0.350	-0.053	0.079	-0.078	6.26E-10	24	0.253
rs7778938	C	0.93	0.343	-0.118	4.35E-05	0.350	-0.075	2.39E-04	0.326	-0.142	0.003	0.347	-0.025	0.513	0.336	-0.066	0.213	0.350	-0.053	0.080	-0.078	7.07E-10	23	0.261
rs11765163	T	0.93	0.344	-0.118	4.15E-05	0.351	-0.074	2.68E-04	0.327	-0.141	0.004	0.348	-0.025	0.509	0.337	-0.068	0.203	0.350	-0.054	0.076	-0.077	7.73E-10	22	0.270
rs11771945	G	0.93	0.344	-0.118	4.15E-05	0.351	-0.074	2.72E-04	0.327	-0.140	0.004	0.348	-0.025	0.509	0.337	-0.068	0.203	0.350	-0.054	0.076	-0.077	8.00E-10	21	0.275
rs7792071	G	0.84	0.446	-0.127	4.51E-06	0.436	-0.057	0.004	0.405	-0.140	0.003	0.420	-0.049	0.174	0.408	-0.082	0.111	0.427	-0.045	0.121	-0.074	8.53E-10	37	0.159
rs6948725	C	0.87	0.440	-0.126	5.90E-06	0.435	-0.058	0.003	0.403	-0.143	0.002	0.419	-0.048	0.184	0.408	-0.080	0.118	0.426	-0.045	0.126	-0.074	9.00E-10	36	0.165
rs6979948	T	0.84	0.435	-0.121	1.60E-05	0.436	-0.057	0.003	0.404	-0.141	0.002	0.420	-0.049	0.177	0.408	-0.081	0.115	0.426	-0.045	0.123	-0.073	1.76E-09	30	0.210
rs11509199	T	0.87	0.432	-0.119	2.07E-05	0.435	-0.058	0.003	0.403	-0.142	0.002	0.419	-0.048	0.181	0.408	-0.081	0.116	0.426	-0.045	0.125	-0.073	1.78E-09	28	0.227

Variant			Generation R			ALSPAC			GOOD			RS-III			RS-II			RS-I			Combined			
SNP	A1	R2*	Freq.	BETA**	P	Freq	BETA**	P	Freq	BETA**	P	Freq	BETA**	P	Freq	BETA**	P	Freq	BETA**	P	BETA**	P	I2	HetP
rs10953933	T	0.87	0.432	-0.119	2.08E-05	0.435	-0.058	0.003	0.403	-0.142	0.002	0.419	-0.048	0.182	0.408	-0.081	0.116	0.426	-0.045	0.125	-0.073	1.78E-09	28	0.227
rs7805374	G	0.84	0.440	-0.119	2.09E-05	0.436	-0.057	0.004	0.405	-0.140	0.003	0.420	-0.049	0.173	0.408	-0.082	0.110	0.427	-0.045	0.121	-0.073	1.84E-09	28	0.226
rs1534019	T	0.84	0.440	-0.119	2.06E-05	0.437	-0.056	0.004	0.405	-0.139	0.003	0.420	-0.049	0.172	0.409	-0.083	0.108	0.427	-0.046	0.120	-0.072	1.98E-09	28	0.228
rs7808120	A	0.84	0.440	-0.119	2.07E-05	0.437	-0.056	0.004	0.405	-0.140	0.003	0.420	-0.049	0.173	0.408	-0.082	0.109	0.427	-0.046	0.120	-0.072	2.07E-09	28	0.224
rs6466774	C	0.84	0.439	-0.119	2.10E-05	0.436	-0.057	0.004	0.404	-0.141	0.003	0.420	-0.049	0.176	0.408	-0.081	0.114	0.426	-0.045	0.122	-0.073	2.13E-09	28	0.228
rs6942652	C	0.87	0.434	-0.117	2.50E-05	0.435	-0.058	0.003	0.403	-0.142	0.002	0.419	-0.048	0.181	0.408	-0.081	0.115	0.426	-0.045	0.125	-0.072	2.15E-09	25	0.245
rs10266975	C	0.87	0.434	-0.117	2.53E-05	0.435	-0.058	0.003	0.403	-0.142	0.002	0.419	-0.048	0.183	0.408	-0.081	0.117	0.426	-0.045	0.126	-0.072	2.15E-09	25	0.245
rs12706333	T	0.87	0.434	-0.117	2.53E-05	0.435	-0.058	0.003	0.403	-0.142	0.002	0.419	-0.048	0.182	0.408	-0.081	0.117	0.426	-0.045	0.126	-0.072	2.15E-09	25	0.245
rs10225276	G	0.87	0.434	-0.117	2.54E-05	0.435	-0.058	0.003	0.403	-0.142	0.002	0.419	-0.048	0.183	0.408	-0.081	0.118	0.426	-0.045	0.126	-0.072	2.15E-09	25	0.245
rs4731007	G	0.87	0.437	-0.120	1.62E-05	0.438	-0.058	0.003	0.404	-0.138	0.003	0.419	-0.046	0.199	0.409	-0.078	0.130	0.427	-0.043	0.137	-0.072	2.21E-09	29	0.215
rs6970383	C	0.87	0.435	-0.119	2.01E-05	0.438	-0.058	0.003	0.404	-0.140	0.003	0.419	-0.047	0.193	0.408	-0.079	0.125	0.426	-0.044	0.133	-0.072	2.22E-09	27	0.229
rs1534014	C	0.84	0.440	-0.119	2.08E-05	0.436	-0.056	0.004	0.405	-0.140	0.003	0.420	-0.049	0.173	0.408	-0.082	0.110	0.427	-0.045	0.120	-0.072	2.26E-09	29	0.219
rs12539571	C	0.87	0.434	-0.117	2.85E-05	0.435	-0.058	0.003	0.402	-0.143	0.002	0.419	-0.048	0.185	0.407	-0.080	0.120	0.426	-0.044	0.127	-0.072	2.33E-09	27	0.235
rs7806875	T	0.84	0.440	-0.118	2.30E-05	0.436	-0.057	0.003	0.404	-0.141	0.002	0.420	-0.049	0.177	0.408	-0.081	0.115	0.426	-0.045	0.123	-0.072	2.34E-09	26	0.237
rs6947934	C	0.87	0.437	-0.119	1.70E-05	0.438	-0.058	0.003	0.404	-0.138	0.003	0.419	-0.046	0.197	0.408	-0.078	0.128	0.427	-0.043	0.135	-0.072	2.43E-09	28	0.224
rs1534017	C	0.78	0.465	-0.120	1.21E-05	0.460	-0.052	0.007	0.424	-0.133	0.004	0.439	-0.042	0.237	0.427	-0.082	0.108	0.447	-0.050	0.089	-0.071	2.99E-09	32	0.197
rs6978080	A	0.78	0.464	-0.120	1.22E-05	0.460	-0.052	0.007	0.424	-0.133	0.004	0.439	-0.042	0.242	0.427	-0.082	0.109	0.447	-0.050	0.090	-0.071	2.99E-09	32	0.197
rs12672898	A	0.78	0.464	-0.120	1.21E-05	0.460	-0.052	0.007	0.424	-0.133	0.004	0.439	-0.042	0.240	0.427	-0.082	0.108	0.447	-0.050	0.089	-0.071	2.99E-09	32	0.197
rs10953932	G	0.78	0.464	-0.120	1.21E-05	0.460	-0.052	0.007	0.424	-0.132	0.004	0.439	-0.042	0.239	0.427	-0.082	0.108	0.447	-0.050	0.089	-0.071	3.09E-09	31	0.201
rs17357115	C	0.78	0.510	-0.119	1.56E-05	0.460	-0.052	0.007	0.424	-0.133	0.004	0.439	-0.042	0.241	0.427	-0.082	0.109	0.447	-0.050	0.089	-0.071	3.30E-09	31	0.206
rs17284918	G	0.78	0.509	-0.118	1.77E-05	0.460	-0.052	0.007	0.424	-0.133	0.004	0.439	-0.042	0.242	0.428	-0.081	0.117	0.447	-0.050	0.091	-0.071	3.94E-09	29	0.217
rs1524503	C	0.60	0.392	-0.128	8.65E-06	0.405	-0.071	4.55E-04	0.356	-0.139	0.004	0.396	-0.018	0.637	0.398	-0.058	0.279	0.416	-0.038	0.202	-0.074	5.24E-09	45	0.106
rs10085590	G	0.60	0.420	-0.121	1.05E-05	0.412	-0.068	5.30E-04	0.359	-0.139	0.003	0.411	-0.017	0.638	0.401	-0.061	0.244	0.421	-0.035	0.227	-0.072	5.44E-09	47	0.095
rs1554634	C	0.60	0.414	-0.120	1.08E-05	0.412	-0.068	5.30E-04	0.359	-0.139	0.003	0.411	-0.017	0.632	0.401	-0.063	0.236	0.420	-0.035	0.228	-0.072	5.71E-09	46	0.101
rs7798060	T	0.60	0.396	-0.121	1.07E-05	0.412	-0.069	4.10E-04	0.359	-0.139	0.003	0.412	-0.018	0.615	0.401	-0.065	0.215	0.421	-0.037	0.195	-0.072	5.91E-09	43	0.116
rs1524506	T	0.78	0.457	-0.111	5.31E-05	0.460	-0.053	0.006	0.424	-0.133	0.004	0.439	-0.042	0.237	0.427	-0.082	0.108	0.447	-0.050	0.089	-0.070	5.97E-09	18	0.295
rs6965195	A	0.86	0.361	-0.108	1.20E-04	0.374	-0.068	6.49E-04	0.345	-0.130	0.006	0.366	-0.017	0.635	0.355	-0.067	0.203	0.370	-0.057	0.056	-0.073	6.03E-09	12	0.338
rs1534016	C	0.86	0.361	-0.108	1.19E-04	0.374	-0.068	6.52E-04	0.345	-0.130	0.006	0.366	-0.017	0.635	0.355	-0.067	0.201	0.371	-0.057	0.055	-0.073	6.03E-09	12	0.338
rs6947494	T	0.60	0.421	-0.121	1.04E-05	0.412	-0.068	5.34E-04	0.359	-0.137	0.004	0.412	-0.015	0.672	0.401	-0.058	0.273	0.421	-0.035	0.222	-0.071	7.04E-09	47	0.093
rs1949803	G	0.75	0.509	-0.121	1.43E-05	0.460	-0.053	0.007	0.420	-0.140	0.003	0.441	-0.042	0.250	0.429	-0.080	0.124	0.450	-0.047	0.115	-0.072	7.97E-09	33	0.188
rs7797976	T	0.60	0.398	-0.123	7.98E-06	0.412	-0.068	5.32E-04	0.359	-0.138	0.004	0.412	-0.016	0.668	0.401	-0.058	0.270	0.421	-0.035	0.223	-0.071	9.75E-09	47	0.093

Variant			Generation R			ALSPAC				GOOD		RS-III			RS-II			RS-I			Combined			
SNP	A1	R2*	Freq.	BETA**	P	Freq	BETA**	P	Freq	BETA**	P	Freq	BETA**	P	Freq	BETA**	P	Freq	BETA**	P	BETA**	P	I2	HetP
rs1917118	T	0.60	0.410	-0.123	9.10E-06	0.412	-0.068	5.38E-04	0.359	-0.137	0.004	0.412	-0.015	0.679	0.401	-0.057	0.282	0.421	-0.035	0.221	-0.071	1.09E-08	47	0.092
rs11770502	G	0.79	0.453	-0.110	1.30E-04	0.450	-0.057	0.004	0.423	-0.144	0.003	0.437	-0.050	0.176	0.426	-0.090	0.086	0.447	-0.044	0.138	-0.072	1.19E-08	15	0.319
rs6954210	A	0.60	0.410	-0.123	9.23E-06	0.412	-0.068	5.47E-04	0.360	-0.135	0.004	0.412	-0.014	0.697	0.402	-0.055	0.295	0.421	-0.036	0.218	-0.071	1.19E-08	47	0.095
rs6947453	T	0.51	0.377	-0.107	1.59E-04	0.388	-0.077	1.93E-04	0.345	-0.150	0.003	0.383	-0.017	0.645	0.369	-0.048	0.378	0.393	-0.039	0.200	-0.073	1.42E-08	34	0.183
rs1404268	A	0.60	0.427	-0.118	3.68E-05	0.406	-0.071	4.62E-04	0.357	-0.138	0.005	0.396	-0.018	0.638	0.402	-0.053	0.318	0.416	-0.038	0.202	-0.071	1.50E-08	37	0.163
rs6970762	T	0.60	0.400	-0.122	1.02E-05	0.412	-0.068	5.49E-04	0.360	-0.130	0.006	0.412	-0.014	0.701	0.402	-0.055	0.298	0.421	-0.036	0.217	-0.070	1.54E-08	44	0.111
rs1357756	T	0.60	0.421	-0.121	1.08E-05	0.412	-0.068	5.51E-04	0.360	-0.130	0.006	0.412	-0.014	0.708	0.402	-0.054	0.305	0.421	-0.036	0.217	-0.070	1.73E-08	44	0.116
rs1534015	A	0.60	0.421	-0.121	1.09E-05	0.412	-0.068	5.54E-04	0.360	-0.129	0.006	0.412	-0.013	0.715	0.402	-0.053	0.316	0.421	-0.036	0.216	-0.069	1.94E-08	44	0.114
rs2968349	C	0.47	0.457	-0.101	2.24E-04	0.468	-0.070	2.89E-04	0.374	-0.120	0.013	0.436	-0.037	0.301	0.428	-0.055	0.282	0.454	-0.026	0.371	-0.067	2.23E-08	11	0.345
rs7786203	A	0.60	0.401	-0.121	1.19E-05	0.412	-0.067	6.66E-04	0.360	-0.129	0.006	0.412	-0.012	0.734	0.402	-0.052	0.321	0.422	-0.036	0.216	-0.069	2.51E-08	44	0.109

Shaded rs7801723 top-hit for association with Head BMD *Correlation coefficients with rs4609139 based on HapMap release22 CEU population.**Effect estimates expressed as standardized adjusted SD per copy of allele (A1).
