# Supplementary material for: Meta-Analysis of Genome-Wide Scans for Total Body BMD in Children and Adults Reveals Allelic Heterogeneity and Age-Specific Effects at the WNT16 Locus
Source: PLoS Genet. 2012 Jul 5;8(7):e1002718. doi: 10.1371/journal.pgen.1002718 (PMC3390371; doi:10.1371/journal.pgen.1002718)
Supplement: Table S4 — Evaluation of the covariates in the Generation R Study. The reduction of SE when weight is included in the model, allows the identification of the genetic signal mapping to 7q31, here represented by rs917727. (PDF) [file pgen.1002718.s007.pdf]

Predictor variables	Model 0 (M0)		Model 1 (M1)		Final Model (MF)	
	Beta	P	Beta	P	Beta	P
**rs9117727**	0.131	6.61E-13	0.097	**1.02E-07**	0.090	**5.90E-11**
**Age (Years)**	0.337	< 2.00E-16	0.317	**< 2.00E-16**	0.101	**3.52E-12**
**Sex (Male)**	0.023	0.209	0.020	**2.55E-01**	0.160	0.242
**Weight (Kg)**	-	-	-	**-**	0.646	**< 2.00E-16**
**PC1**	-	-	-0.149	**9.25E-16**	-0.104	**1.28E-13**
**PC2**	-	-	0.100	**2.37E-08**	0.046	**0.001**
**PC3**	-	-	-0.010	**5.84E-01**	-0.038	**0.005**
**PC4**	-	-	0.029	0.101	0.005	0.717
**PC5**	-	-	-0.025	0.162	0.007	0.586
**PC6**	-	-	-0.027	0.135	-0.034	**0.013**
**PC7**	-	-	-0.004	0.801	-0.001	0.936
**PC8**	-	-	0.025	0.162	0.024	0.075
**PC9**	-	-	0.033	0.065	0.033	**0.015**
**PC10**	-	-	-0.012	0.516	0.007	0.621
**PC11**	-	-	-0.008	0.648	-0.010	0.466
**PC12**	-	-	-0.017	0.345	-0.010	0.468
**PC13**	-	-	0.027	0.127	0.022	0.1
**PC14**	-	-	-0.018	0.313	-0.007	0.605
**PC15**	-	-	0.025	0.163	0.020	0.143
**PC16**	-	-	0.000	0.99	-0.020	0.134
**PC17**	-	-	0.001	0.954	0.004	0.762
**PC18**	-	-	0.001	0.966	-0.019	0.164
**PC19**	-	-	-0.007	0.691	0.002	0.891
**PC20**	-	-	-0.007	0.692	-0.001	0.954
