# Supplementary material for: Meta-Analysis of Genome-Wide Scans for Total Body BMD in Children and Adults Reveals Allelic Heterogeneity and Age-Specific Effects at the WNT16 Locus
Source: PLoS Genet. 2012 Jul 5;8(7):e1002718. doi: 10.1371/journal.pgen.1002718 (PMC3390371; doi:10.1371/journal.pgen.1002718)
Supplement: Table S5 — SNPs showing GWS association with skull BMD. (PDF) [file pgen.1002718.s008.pdf]

			DISCOVERY			REPLICATION															COMBINED			
**VARIANT**			**Generation R**			**ALSPAC**			**GOOD**			**RS-III**			**RS-II**			**RS-I**						
			**n= 2,660**			**n= 5,334**			**n= 938**			**n= 1,594**			**n= 750**			**n= 2,436**			**n=13,712**			
**SNP**	**A1**	**R2***	**Freq.**	**BETA****	**P**	**Freq**	**BETA****	**P**	**Freq**	**BETA****	**P**	**Freq**	**BETA****	**P**	**Freq**	**BETA****	**P**	**Freq**	**BETA****	**P**	**BETA****	**P**	**I2**	**HetP**
**rs7801723**	T	1	0.374	-0.144	2.75E-07	0.386	-0.178	**6.00E-19**	0.333	-0.173	3.42E-04	0.383	-0.089	0.014	0.367	-0.075	0.154	0.393	-0.071	0.017	-0.137	**8.91E-28**	61	0.026
rs6950680	G	1	0.374	-0.144	2.62E-07	0.385	-0.176	**1.22E-18**	0.333	-0.173	3.39E-04	0.383	-0.09	0.014	0.367	-0.075	0.154	0.393	-0.07	0.017	-0.136	**1.86E-27**	60	0.030
rs12706318	G	1	0.378	-0.139	6.79E-07	0.386	-0.178	**6.70E-19**	0.333	-0.173	3.42E-04	0.383	-0.089	0.014	0.367	-0.075	0.154	0.393	-0.071	0.017	-0.136	**2.14E-27**	61	0.027
rs13232048	T	1	0.378	-0.139	6.80E-07	0.386	-0.178	**7.00E-19**	0.333	-0.173	3.42E-04	0.383	-0.089	0.014	0.367	-0.075	0.154	0.393	-0.071	0.017	-0.136	**2.14E-27**	61	0.027
rs798943	A	1	0.384	-0.138	8.13E-07	0.386	-0.178	**6.18E-19**	0.333	-0.173	3.37E-04	0.383	-0.089	0.015	0.367	-0.075	0.155	0.393	-0.071	0.017	-0.135	**2.54E-27**	60	0.027
rs6952113	A	1	0.379	-0.139	6.80E-07	0.385	-0.177	**7.86E-19**	0.333	-0.173	3.41E-04	0.383	-0.09	0.014	0.367	-0.075	0.154	0.393	-0.071	0.017	-0.135	**2.72E-27**	60	0.030
rs872007	T	1	0.379	-0.139	6.88E-07	0.385	-0.177	**9.53E-19**	0.333	-0.173	3.41E-04	0.383	-0.09	0.014	0.367	-0.075	0.154	0.393	-0.071	0.017	-0.135	**2.72E-27**	60	0.030
rs13245690	G	0.93	0.376	-0.144	2.90E-07	0.387	-0.177	**1.06E-18**	0.337	-0.172	3.44E-04	0.385	-0.089	0.015	0.370	-0.071	0.176	0.396	-0.067	0.023	-0.134	**2.98E-27**	63	0.018
rs10275439	A	0.90	0.386	-0.134	1.55E-06	0.383	-0.176	**1.48E-18**	0.330	-0.172	3.83E-04	0.380	-0.088	0.017	0.362	-0.072	0.172	0.390	-0.071	0.017	-0.133	**1.35E-26**	60	0.030
rs10261671	T	0.80	0.388	-0.134	1.83E-06	0.386	-0.176	**1.35E-18**	0.334	-0.173	3.55E-04	0.383	-0.088	0.016	0.367	-0.072	0.171	0.393	-0.069	0.020	-0.133	**1.72E-26**	61	0.027
rs2536150	C	0.10	0.215	-0.2	**4.58E-09**	0.176	-0.173	**1.27E-11**	0.168	-0.173	0.006	0.180	-0.104	0.026	0.174	-0.166	0.017	0.182	-0.143	0.000	-0.165	**2.59E-25**	0	0.671
rs1524503	C	0.77	0.392	-0.133	4.61E-06	0.405	-0.172	**3.63E-17**	0.356	-0.186	1.44E-04	0.396	-0.095	0.011	0.398	-0.073	0.176	0.416	-0.054	0.071	-0.130	**4.66E-25**	65	0.015
rs13223036	G	0.62	0.345	-0.137	1.49E-06	0.371	-0.17	**3.09E-17**	0.299	-0.147	0.004	0.351	-0.081	0.028	0.340	-0.057	0.286	0.361	-0.073	0.015	-0.129	**1.05E-24**	56	0.045
rs7798060	T	0.77	0.396	-0.122	1.03E-05	0.412	-0.165	**8.43E-17**	0.359	-0.177	1.80E-04	0.412	-0.1	0.006	0.401	-0.088	0.099	0.421	-0.059	0.044	-0.126	**1.60E-24**	56	0.044
rs7797976	T	0.77	0.398	-0.123	9.58E-06	0.412	-0.164	**1.36E-16**	0.359	-0.177	1.79E-04	0.412	-0.098	0.007	0.401	-0.082	0.124	0.421	-0.055	0.057	-0.125	**5.44E-24**	59	0.034
rs6970762	T	0.77	0.400	-0.123	1.07E-05	0.412	-0.164	**1.41E-16**	0.360	-0.176	1.94E-04	0.412	-0.097	0.007	0.402	-0.079	0.140	0.421	-0.055	0.060	0.125	**7.25E-24**	59	0.033
rs1404268	A	0.77	0.427	-0.117	5.17E-05	0.406	-0.171	**3.81E-17**	0.357	-0.184	1.50E-04	0.396	-0.095	0.011	0.402	-0.076	0.155	0.416	-0.054	0.071	-0.127	**7.29E-24**	64	0.016
rs1917118	T	0.77	0.410	-0.119	1.97E-05	0.412	-0.164	**1.37E-16**	0.359	-0.177	1.79E-04	0.412	-0.098	0.007	0.401	-0.081	0.130	0.421	-0.055	0.058	-0.124	**1.08E-23**	59	0.033
rs6954210	A	0.77	0.410	-0.119	1.93E-05	0.412	-0.164	**1.40E-16**	0.360	-0.177	1.81E-04	0.412	-0.098	0.007	0.402	-0.079	0.138	0.421	-0.055	0.060	-0.124	**1.18E-23**	59	0.032
rs6947453	T	0.93	0.377	-0.128	7.62E-06	0.388	-0.177	**1.66E-17**	0.345	-0.175	4.76E-04	0.383	-0.088	0.020	0.369	-0.08	0.148	0.393	-0.057	0.063	-0.129	**1.47E-23**	64	0.017
rs1554634	C	0.77	0.414	-0.113	4.03E-05	0.412	-0.164	**1.09E-16**	0.359	-0.177	1.80E-04	0.411	-0.099	0.006	0.401	-0.086	0.104	0.420	-0.056	0.054	-0.123	**1.76E-23**	58	0.036
rs7786203	A	0.77	0.401	-0.119	1.96E-05	0.412	-0.164	**1.24E-16**	0.360	-0.176	1.97E-04	0.412	-0.096	0.008	0.402	-0.076	0.154	0.422	-0.054	0.064	-0.123	**2.01E-23**	60	0.029
rs10085590	G	0.77	0.420	-0.111	5.44E-05	0.412	-0.164	**1.16E-16**	0.359	-0.177	1.80E-04	0.411	-0.099	0.006	0.401	-0.085	0.109	0.421	-0.056	0.054	-0.123	**2.53E-23**	58	0.035
rs6947494	T	0.77	0.421	-0.112	4.97E-05	0.412	-0.164	**1.37E-16**	0.359	-0.177	1.79E-04	0.412	-0.098	0.007	0.401	-0.082	0.125	0.421	-0.055	0.057	-0.123	**3.14E-23**	59	0.031
rs1357756	T	0.77	0.421	-0.113	4.81E-05	0.412	-0.164	**1.41E-16**	0.360	-0.176	1.95E-04	0.412	-0.097	0.007	0.402	-0.078	0.143	0.421	-0.055	0.061	-0.123	**3.72E-23**	60	0.030
rs1534015	A	0.77	0.421	-0.113	4.80E-05	0.412	-0.164	**1.42E-16**	0.360	-0.176	1.97E-04	0.412	-0.097	0.007	0.402	-0.076	0.150	0.421	-0.054	0.061	-0.122	**4.71E-23**	60	0.028
rs2952559	C	0.09	0.265	-0.186	**2.51E-08**	0.183	-0.158	**7.29E-10**	0.165	-0.174	0.007	0.180	-0.128	0.007	0.184	-0.147	0.035	0.184	-0.128	0.001	-0.156	**6.22E-23**	0	0.873

**Variant**			**Generation R**			**ALSPAC**			**GOOD**			**RS-III**			**RS-II**			**RS-I**			**Combined**			
**SNP**	**A1**	**R2***	**Freq.**	**BETA****	**P**	**Freq**	**BETA****	**P**	**Freq**	**BETA****	**P**	**Freq**	**BETA****	**P**	**Freq**	**BETA****	**P**	**Freq**	**BETA****	**P**	**BETA****	**P**	**I2**	**HetP**
rs6954757	A	0.56	0.339	-0.136	1.55E-06	0.364	-0.161	**1.38E-15**	0.295	-0.15	0.003	0.341	-0.079	0.034	0.329	-0.027	0.621	0.350	-0.074	0.014	-0.123	**8.52E-23**	56	0.045
rs6466769	G	0.56	0.339	-0.136	1.62E-06	0.364	-0.16	**1.51E-15**	0.295	-0.15	0.003	0.341	-0.078	0.035	0.330	-0.026	0.627	0.351	-0.073	0.016	-0.123	**1.53E-22**	56	0.044
rs1534016	C	0.68	0.361	-0.141	6.61E-07	0.374	-0.154	**2.28E-14**	0.345	-0.182	1.21E-04	0.366	-0.084	0.023	0.355	-0.019	0.715	0.371	-0.058	0.054	-0.121	**2.29E-22**	64	0.015
rs6965195	A	0.68	0.361	-0.141	6.66E-07	0.374	-0.154	**2.28E-14**	0.345	-0.182	1.22E-04	0.366	-0.084	0.023	0.355	-0.019	0.717	0.370	-0.058	0.054	-0.121	**2.29E-22**	64	0.015
rs1917113	A	0.56	0.339	-0.136	1.64E-06	0.364	-0.159	**2.37E-15**	0.296	-0.151	0.003	0.341	-0.077	0.038	0.331	-0.024	0.662	0.352	-0.069	0.023	-0.122	**3.43E-22**	58	0.036
rs6967129	G	0.56	0.346	-0.148	3.59E-07	0.353	-0.156	**2.08E-14**	0.327	-0.149	0.002	0.350	-0.094	0.012	0.340	-0.017	0.749	0.353	-0.058	0.054	-0.122	**3.88E-22**	61	0.024
rs12706334	G	0.56	0.346	-0.147	3.78E-07	0.353	-0.156	**2.32E-14**	0.326	-0.148	0.002	0.350	-0.094	0.012	0.340	-0.017	0.753	0.353	-0.058	0.054	-0.122	**4.06E-22**	61	0.025
rs12673968	A	0.56	0.347	-0.132	3.19E-06	0.364	-0.159	**2.37E-15**	0.296	-0.151	0.003	0.341	-0.077	0.038	0.331	-0.024	0.662	0.352	-0.069	0.023	-0.121	**6.41E-22**	58	0.038
rs4731006	T	0.56	0.346	-0.148	3.85E-07	0.356	-0.154	**4.20E-14**	0.327	-0.146	0.002	0.350	-0.094	0.012	0.340	-0.016	0.760	0.354	-0.058	0.054	-0.121	**7.51E-22**	60	0.028
rs6466767	G	0.56	0.348	-0.133	2.93E-06	0.364	-0.159	**2.30E-15**	0.296	-0.151	0.003	0.342	-0.075	0.044	0.331	-0.019	0.723	0.354	-0.068	0.026	-0.120	**9.25E-22**	60	0.030
**rs4609139**	T	0.56	0.355	-0.147	3.52E-07	0.356	-0.154	**4.88E-14**	0.328	-0.145	0.003	0.351	-0.093	0.013	0.341	-0.016	0.764	0.354	-0.058	0.054	-0.121	**1.00E-21**	60	0.028
rs2968345	G	0.56	0.359	-0.133	2.81E-06	0.365	-0.159	**2.28E-15**	0.296	-0.151	0.003	0.342	-0.073	0.050	0.332	-0.017	0.748	0.355	-0.066	0.028	-0.120	**1.57E-21**	61	0.024
rs10500083	C	0.62	0.351	-0.154	1.04E-07	0.350	-0.155	**4.26E-14**	0.326	-0.164	0.001	0.347	-0.09	0.017	0.336	-0.025	0.639	0.350	-0.056	0.067	-0.122	**1.91E-21**	62	0.021
rs2272196	A	0.62	0.351	-0.154	1.08E-07	0.350	-0.155	**4.45E-14**	0.326	-0.163	7.28E-04	0.347	-0.09	0.017	0.336	-0.025	0.642	0.350	-0.056	0.067	-0.122	**2.02E-21**	62	0.022
rs7795660	T	0.62	0.344	-0.154	1.19E-07	0.350	-0.155	**4.56E-14**	0.326	-0.163	7.41E-04	0.347	-0.09	0.017	0.336	-0.025	0.643	0.350	-0.056	0.067	-0.122	**2.02E-21**	62	0.022
rs7795692	G	0.62	0.351	-0.154	1.12E-07	0.350	-0.155	**4.61E-14**	0.326	-0.163	7.46E-04	0.347	-0.09	0.017	0.336	-0.025	0.643	0.350	-0.056	0.067	-0.122	**2.02E-21**	62	0.022
rs11771945	G	0.62	0.344	-0.154	1.18E-07	0.351	-0.155	**3.88E-14**	0.327	-0.164	6.61E-04	0.348	-0.09	0.017	0.337	-0.026	0.634	0.350	-0.055	0.068	-0.122	**2.11E-21**	62	0.021
rs10480747	C	0.62	0.341	-0.154	1.27E-07	0.351	-0.155	**4.11E-14**	0.326	-0.164	6.86E-04	0.347	-0.09	0.017	0.336	-0.025	0.637	0.350	-0.055	0.068	-0.122	**2.20E-21**	63	0.020
rs7778938	C	0.62	0.343	-0.153	1.40E-07	0.350	-0.155	**4.63E-14**	0.326	-0.163	7.52E-04	0.347	-0.09	0.017	0.336	-0.025	0.644	0.350	-0.056	0.067	-0.122	**2.34E-21**	62	0.022
rs11765163	T	0.62	0.344	-0.153	1.19E-07	0.351	-0.155	**3.95E-14**	0.327	-0.164	6.70E-04	0.348	-0.09	0.017	0.337	-0.026	0.634	0.350	-0.055	0.068	-0.122	**2.44E-21**	62	0.021
rs2110281	A	0.56	0.351	-0.135	2.24E-06	0.365	-0.159	**2.27E-15**	0.297	-0.151	0.003	0.343	-0.071	0.057	0.332	-0.016	0.762	0.355	-0.066	0.028	-0.119	**3.15E-21**	62	0.022
rs10228519	G	0.50	0.488	-0.102	1.72E-04	0.486	-0.144	**1.11E-13**	0.463	-0.113	0.014	0.495	-0.071	0.046	0.481	-0.078	0.121	0.499	-0.066	0.022	-0.108	**1.20E-19**	31	0.204
rs6971407	T	0.45	0.491	0.088	0.001	0.487	0.141	**2.85E-13**	0.510	0.123	0.007	0.475	0.086	0.017	0.484	0.091	0.074	0.472	0.058	0.045	0.106	**7.50E-19**	30	0.213
rs6972481	C	0.45	0.492	0.088	0.001	0.514	0.141	**2.95E-13**	0.510	0.123	0.007	0.475	0.085	0.017	0.484	0.092	0.071	0.472	0.058	0.045	0.106	**7.82E-19**	30	0.211
**rs917727**	T	0.04	0.296	0.119	2.02E-04	0.273	0.085	1.77E-04	0.238	0.167	0.003	0.274	0.141	4.73E-04	0.263	0.113	0.061	0.269	0.143	1.64E-05	0.116	**1.90E-16**	0	0.586
rs1005400	A	0.28	0.468	-0.107	7.99E-05	0.462	-0.13	**2.36E-11**	0.426	-0.075	0.101	0.452	-0.06	0.089	0.439	-0.046	0.376	0.455	-0.062	0.032	-0.098	**3.41E-16**	28	0.227
rs917726	T	0.04	0.282	0.114	3.61E-04	0.273	0.085	1.78E-04	0.238	0.167	0.003	0.274	0.141	4.75E-04	0.263	0.113	0.061	0.269	0.143	1.66E-05	0.115	**3.90E-16**	0	0.592
rs1917114	A	0.28	0.470	-0.11	4.87E-05	0.462	-0.129	**2.58E-11**	0.426	-0.075	0.102	0.452	-0.059	0.093	0.440	-0.046	0.377	0.456	-0.06	0.040	-0.097	**4.09E-16**	29	0.216
rs7776725	*C*	*0.04*	*0.264*	*0.12*	*2.29E-04*	*0.462*	*0.086*	*1.91E-04*	*0.239*	*0.176*	*0.003*	*0.274*	*0.142*	*4.81E-04*	*0.263*	*0.114*	*0.060*	*0.269*	*0.145*	*1.71E-05*	*0.117*	***4.74E-16***	*0*	*0.567*
rs3801382	G	0.04	0.275	0.104	7.44E-04	0.462	0.083	1.56E-04	0.238	0.164	0.003	0.274	0.139	4.95E-04	0.263	0.111	0.062	0.269	0.142	1.71E-05	0.111	**8.14E-16**	0	0.549
rs718766	C	0.04	0.272	0.11	6.15E-04	0.462	0.085	1.82E-04	0.238	0.169	0.003	0.274	0.141	4.76E-04	0.263	0.113	0.061	0.269	0.144	1.69E-05	0.114	**8.78E-16**	0	0.578

Variant			Generation R			ALSPAC			GOOD			RS-III			RS-II			RS-I			Combined			
SNP	A1	R2*	Freq.	BETA**	P	Freq	BETA**	P	Freq	BETA**	P	Freq	BETA**	P	Freq	BETA**	P	Freq	BETA**	P	BETA**	P	I2	HetP
rs2908004	A	0.05	0.501	0.102	2.06E-04	0.462	0.072	3.09E-04	0.431	0.071	0.145	0.456	0.127	0.001	0.437	0.157	0.003	0.449	0.14	4.97E-06	0.100	**9.11E-16**	12	0.340
rs2536189	*G*	*0.05*	*0.498*	*0.099*	*2.72E-04*	*0.442*	*0.073*	*3.07E-04*	*0.431*	*0.071*	*0.145*	*0.456*	*0.127*	*0.001*	*0.437*	*0.157*	*0.003*	*0.449*	*0.14*	*5.01E-06*	*0.100*	***1.07E-15***	*10*	*0.355*
rs2536149	A	0.05	0.139	-0.256	**3.67E-10**	0.115	-0.151	9.49E-07	0.085	-0.196	0.017	0.102	-0.076	0.199	0.103	-0.114	0.188	0.110	-0.068	0.137	0.152	**3.30E-15**	58	0.036
rs3801387	G	0.04	0.274	0.092	0.003	0.272	0.083	1.51E-04	0.238	0.166	0.002	0.275	0.139	4.66E-04	0.263	0.11	0.065	0.270	0.138	2.67E-05	0.108	**4.68E-15**	0	0.530
rs2968349	C	0.63	0.457	-0.112	5.23E-05	0.468	-0.129	2.74E-11	0.374	-0.121	0.012	0.436	-0.051	0.149	0.428	-0.048	0.356	0.454	-0.03	0.295	-0.095	**5.20E-15**	55	0.048
rs2536148	T	0.05	0.153	-0.241	**2.49E-09**	0.111	-0.154	1.00E-06	0.083	-0.21	0.012	0.101	-0.071	0.237	0.102	-0.119	0.172	0.108	-0.06	0.198	-0.150	**1.27E-14**	56	0.044
rs4727924	T	0.06	0.467	0.117	6.03E-05	0.459	0.067	0.001	0.454	0.063	0.192	0.477	0.115	0.001	0.463	0.156	0.003	0.476	0.118	6.65E-05	0.096	**1.93E-14**	4	0.389
rs2536182	G	0.05	0.471	0.101	3.14E-04	0.454	0.065	9.08E-04	0.439	0.095	0.047	0.466	0.119	0.001	0.452	0.163	0.002	0.463	0.111	1.73E-04	0.094	**2.07E-14**	0	0.438
rs2536180	C	0.06	0.495	0.098	3.33E-04	0.463	0.063	0.001	0.456	0.061	0.193	0.478	0.114	0.001	0.465	0.153	0.003	0.477	0.116	6.68E-05	0.090	**6.63E-14**	5	0.382
rs2254595	C	0.06	0.504	0.096	3.85E-04	0.463	0.063	0.001	0.456	0.061	0.193	0.478	0.114	0.001	0.464	0.153	0.003	0.477	0.116	6.38E-05	0.089	**8.51E-14**	5	0.387
rs798903	C	0.32	0.498	0.107	2.04E-04	0.463	0.118	**2.67E-09**	0.528	0.147	0.001	0.510	0.04	0.266	0.517	0.001	0.989	0.504	0.06	0.038	0.092	**1.64E-13**	49	0.083
rs3757552	G	0	0.112	-0.176	6.49E-05	0.122	-0.125	3.04E-05	0.117	-0.198	0.006	0.106	-0.094	0.092	0.122	-0.212	0.005	0.118	-0.099	0.024	-0.136	**4.06E-13**	0	0.554
rs10953925	G	0.23	0.457	-0.096	5.43E-04	0.506	-0.116	**2.16E-09**	0.428	-0.092	0.043	0.481	-0.08	0.022	0.474	-0.017	0.744	0.495	-0.033	0.257	-0.087	**6.07E-13**	36	0.166
rs13246689	C	0.23	0.456	-0.096	5.63E-04	0.507	-0.116	**2.43E-09**	0.429	-0.091	0.046	0.482	-0.08	0.022	0.475	-0.017	0.744	0.495	-0.032	0.266	-0.086	**7.02E-13**	37	0.159
rs10953924	C	0.23	0.453	-0.094	7.36E-04	0.507	-0.116	**2.18E-09**	0.428	-0.092	0.043	0.481	-0.08	0.022	0.474	-0.017	0.744	0.495	-0.033	0.260	-0.086	**7.60E-13**	36	0.168
rs11766764	T	0.23	0.454	-0.094	6.92E-04	0.507	-0.116	**2.22E-09**	0.428	-0.092	0.044	0.481	-0.08	0.022	0.474	-0.017	0.744	0.495	-0.032	0.261	-0.086	**8.43E-13**	37	0.161
rs3779381	G	0.05	0.264	0.083	0.009	0.255	0.074	0.001	0.227	0.155	0.006	0.254	0.13	0.002	0.241	0.101	0.106	0.250	0.154	9.02E-06	0.103	**8.84E-13**	7	0.372
rs2707466	*T*	*0.06*	*0.485*	*0.096*	*5.04E-04*	*0.425*	*0.058*	*0.005*	*0.418*	*0.056*	*0.255*	*0.440*	*0.119*	*0.002*	*0.424*	*0.166*	*0.002*	*0.435*	*0.132*	*2.05E-05*	*0.092*	***9.98E-13***	*31*	*0.204*
rs13247600	C	0.03	0.064	-0.18	0.005	0.076	-0.186	2.14E-05	0.089	-0.158	0.100	0.082	-0.173	0.018	0.084	-0.181	0.117	0.079	-0.239	8.74E-05	-0.191	**1.02E-12**	0	0.974
rs12706314	G	0.53	0.451	0.089	0.001	0.443	0.116	**2.62E-09**	0.487	0.088	0.055	0.446	0.047	0.188	0.461	0.08	0.121	0.439	0.036	0.210	0.085	**1.48E-12**	25	0.247
rs798949	C	0.53	0.441	0.088	0.001	0.443	-0.116	**2.51E-09**	0.487	0.088	0.055	0.446	0.047	0.188	0.461	0.08	0.121	0.439	0.036	0.210	0.085	**1.65E-12**	25	0.248
rs2110280	C	0.22	0.459	-0.091	9.69E-04	0.515	-0.115	**3.31E-09**	0.440	-0.105	0.021	0.487	-0.072	0.040	0.486	-0.01	0.848	0.503	-0.032	0.267	-0.085	**1.96E-12**	40	0.137
rs6466766	C	0.23	0.453	-0.095	6.54E-04	0.506	-0.118	**2.49E-09**	0.429	-0.091	0.049	0.482	-0.08	0.022	0.475	-0.017	0.745	0.496	-0.032	0.275	-0.086	**2.85E-12**	38	0.156
rs10953932	G	0.55	0.464	-0.118	1.98E-05	0.460	-0.104	8.11E-08	0.424	-0.148	0.001	0.439	-0.049	0.167	0.427	-0.015	0.772	0.447	-0.019	0.515	-0.084	**3.87E-12**	59	0.032
rs12672898	A	0.55	0.464	-0.118	1.94E-05	0.460	-0.104	8.09E-08	0.424	-0.148	0.001	0.439	-0.049	0.166	0.427	-0.015	0.773	0.447	-0.019	0.518	-0.084	**3.87E-12**	59	0.032
rs1534017	C	0.55	0.465	-0.118	1.94E-05	0.460	-0.104	8.22E-08	0.424	-0.148	0.001	0.439	-0.049	0.167	0.427	-0.015	0.774	0.447	-0.019	0.508	-0.084	**3.87E-12**	59	0.032
rs6978080	A	0.55	0.464	-0.118	1.81E-05	0.460	-0.104	8.05E-08	0.424	-0.147	0.001	0.439	-0.049	0.166	0.427	-0.015	0.773	0.447	-0.019	0.528	-0.084	**4.03E-12**	59	0.032
rs1524506	T	0.55	0.457	-0.116	2.75E-05	0.460	-0.104	8.29E-08	0.424	-0.148	0.001	0.439	-0.049	0.167	0.427	-0.015	0.775	0.447	-0.02	0.507	-0.084	**4.35E-12**	58	0.036
rs10215148	A	0.43	0.439	0.085	0.002	0.442	0.117	**2.25E-09**	0.487	0.086	0.062	0.444	0.049	0.173	0.459	0.082	0.109	0.437	0.038	0.187	0.084	**5.24E-12**	19	0.288
rs10215475	C	0.43	0.439	0.085	0.002	0.442	0.117	**2.22E-09**	0.487	0.086	0.062	0.444	0.049	0.173	0.459	0.082	0.109	0.437	0.038	0.187	0.084	**5.24E-12**	19	0.288
rs1125447	A	0.43	0.439	0.085	0.002	0.442	0.117	**2.27E-09**	0.487	0.086	0.061	0.444	0.049	0.174	0.459	0.082	0.109	0.437	0.038	0.188	0.084	**5.24E-12**	19	0.288

Variant			Generation R			ALSPAC			GOOD			RS-III			RS-II			RS-I			Combined			
SNP	A1	R2*	Freq.	BETA**	P	Freq	BETA**	P	Freq	BETA**	P	Freq	BETA**	P	Freq	BETA**	P	Freq	BETA**	P	BETA**	P	I2	HetP
rs1357755	C	0.43	0.439	0.085	0.002	0.442	0.117	**2.19E-09**	0.487	0.086	0.062	0.444	0.049	0.172	0.459	0.082	0.108	0.437	0.038	0.186	0.084	**5.24E-12**	19	0.288
rs2402560	T	0.43	0.439	0.085	0.002	0.442	0.117	**2.32E-09**	0.487	0.086	0.061	0.444	0.048	0.174	0.459	0.082	0.109	0.437	0.038	0.188	0.084	**5.60E-12**	20	0.283
rs11531545	G	0.43	0.433	0.084	0.002	0.442	0.117	**2.21E-09**	0.487	0.086	0.062	0.444	0.049	0.173	0.459	0.082	0.108	0.437	0.038	0.186	0.084	**5.89E-12**	19	0.288
rs10251139	C	0.53	0.441	0.086	0.002	0.443	0.116	**2.79E-09**	0.486	0.088	0.057	0.446	0.047	0.186	0.461	0.079	0.122	0.439	0.036	0.210	0.084	**8.22E-12**	22	0.271
rs10235934	G	0.43	0.439	0.084	0.002	0.442	0.117	**2.30E-09**	0.487	0.086	0.061	0.444	0.048	0.174	0.459	0.082	0.109	0.437	0.038	0.188	0.084	**8.91E-12**	20	0.283
rs1524498	C	0.53	0.433	0.085	0.002	0.443	0.116	**2.73E-09**	0.486	0.088	0.056	0.446	0.047	0.188	0.461	0.08	0.121	0.439	0.036	0.210	0.083	**8.94E-12**	22	0.272
rs1558541	C	0.53	0.433	0.085	0.002	0.443	0.116	**2.59E-09**	0.487	0.088	0.056	0.446	0.047	0.188	0.461	0.08	0.121	0.439	0.036	0.210	0.083	**8.94E-12**	22	0.272
rs1949803	G	0.58	0.509	-0.108	1.30E-04	0.460	-0.11	**2.85E-08**	0.420	-0.152	0.001	0.441	-0.047	0.197	0.429	-0.019	0.713	0.450	-0.021	0.491	-0.085	**9.52E-12**	57	0.043
rs10246521	T	0.53	0.439	0.085	0.002	0.442	0.115	**4.12E-09**	0.487	0.088	0.057	0.446	0.047	0.183	0.460	0.081	0.115	0.438	0.037	0.205	0.083	**9.67E-12**	18	0.297
rs1112208	G	0.53	0.433	0.085	0.002	0.442	0.115	**4.12E-09**	0.487	0.088	0.057	0.446	0.047	0.183	0.460	0.081	0.116	0.438	0.037	0.206	0.083	**9.67E-12**	18	0.297
rs2177578	A	0.53	0.433	0.085	0.002	0.442	0.115	**3.83E-09**	0.487	0.088	0.056	0.446	0.047	0.185	0.461	0.08	0.118	0.439	0.037	0.206	0.083	**1.00E-11**	18	0.297
rs10248011	A	0.53	0.433	0.085	0.002	0.443	0.115	**3.54E-09**	0.487	0.088	0.056	0.446	0.047	0.186	0.461	0.08	0.118	0.439	0.036	0.207	0.083	**1.11E-11**	20	0.287
rs10216123	G	0.38	0.421	0.072	0.010	0.414	0.116	**3.56E-09**	0.461	0.101	0.028	0.414	0.066	0.071	0.423	0.092	0.075	0.408	0.03	0.308	0.084	**1.17E-11**	25	0.250
rs10259383	T	0.53	0.441	0.087	0.002	0.443	0.115	**3.38E-09**	0.487	0.088	0.056	0.446	0.047	0.186	0.461	0.08	0.119	0.439	0.036	0.207	0.083	**1.27E-11**	20	0.285
rs17284918	G	0.55	0.509	-0.103	2.12E-04	0.460	-0.104	7.79E-08	0.424	-0.148	0.001	0.439	-0.049	0.167	0.428	-0.018	0.737	0.447	-0.019	0.526	-0.081	**1.77E-11**	55	0.051
rs6979948	T	0.49	0.435	-0.129	4.78E-06	0.436	-0.101	2.81E-07	0.404	-0.124	0.008	0.420	-0.053	0.146	0.408	-0.019	0.708	0.426	-0.018	0.550	-0.083	**2.10E-11**	57	0.042
rs1534019	T	0.49	0.440	-0.125	8.28E-06	0.437	-0.102	2.05E-07	0.405	-0.127	0.006	0.420	-0.053	0.146	0.409	-0.021	0.692	0.427	-0.016	0.578	-0.082	**2.32E-11**	57	0.042
rs7792071	G	0.49	0.446	-0.126	7.08E-06	0.436	-0.101	2.36E-07	0.405	-0.126	0.007	0.420	-0.053	0.146	0.408	-0.02	0.699	0.427	-0.017	0.566	-0.082	**2.50E-11**	56	0.044
rs1917112	G	0.36	0.434	0.095	5.21E-04	0.435	0.112	**1.03E-08**	0.497	0.061	0.189	0.455	0.049	0.165	0.465	0.079	0.126	0.446	0.032	0.272	0.081	**2.51E-11**	24	0.256
rs1534014	C	0.49	0.440	-0.125	8.83E-06	0.436	-0.101	2.19E-07	0.405	-0.127	0.006	0.420	-0.053	0.146	0.408	-0.02	0.696	0.427	-0.017	0.571	-0.082	**2.68E-11**	56	0.046
rs7808120	A	0.49	0.440	-0.125	8.67E-06	0.437	-0.101	2.17E-07	0.405	-0.127	0.006	0.420	-0.053	0.146	0.408	-0.02	0.696	0.427	-0.017	0.573	-0.082	**2.68E-11**	56	0.046
rs12706326	A	0.10	0.044	-0.213	0.005	0.051	-0.245	3.80E-06	0.053	-0.102	0.398	0.054	-0.18	0.047	0.057	-0.238	0.084	0.055	-0.189	0.003	-0.208	**2.75E-11**	0	0.914
rs17357115	C	0.55	0.510	-0.1	2.83E-04	0.460	-0.104	8.06E-08	0.424	-0.147	0.001	0.439	-0.049	0.166	0.427	-0.015	0.773	0.447	-0.019	0.522	-0.081	**2.76E-11**	54	0.052
rs7805374	G	0.49	0.440	-0.125	9.10E-06	0.436	-0.101	2.30E-07	0.405	-0.126	0.007	0.420	-0.053	0.146	0.408	-0.02	0.698	0.427	-0.017	0.568	-0.082	**2.78E-11**	56	0.046
rs17143161	G	0.36	0.443	0.09	9.79E-04	0.434	0.111	**1.16E-08**	0.496	0.062	0.181	0.455	0.051	0.146	0.467	0.08	0.118	0.448	0.036	0.221	0.081	**3.00E-11**	12	0.339
rs6466774	C	0.49	0.439	-0.124	9.61E-06	0.436	-0.101	2.59E-07	0.404	-0.126	0.007	0.420	-0.053	0.146	0.408	-0.02	0.705	0.426	-0.017	0.556	-0.082	**3.57E-11**	55	0.049
rs7806875	T	0.49	0.440	-0.124	1.02E-05	0.436	-0.101	2.80E-07	0.404	-0.124	0.008	0.420	-0.053	0.146	0.408	-0.019	0.707	0.426	-0.018	0.551	-0.082	**3.59E-11**	54	0.053
rs10274486	C	0.38	0.411	0.068	0.015	0.415	0.108	3.76E-08	0.460	0.102	0.027	0.415	0.065	0.075	0.424	0.09	0.082	0.407	0.027	0.358	0.079	**1.39E-10**	16	0.314
rs6942652	C	0.47	0.434	-0.114	5.10E-05	0.435	-0.099	4.26E-07	0.403	-0.123	0.008	0.419	-0.052	0.146	0.408	-0.02	0.704	0.426	-0.018	0.537	-0.079	**1.66E-10**	49	0.084
rs10953933	T	0.47	0.432	-0.114	5.01E-05	0.435	-0.099	4.44E-07	0.403	-0.122	0.009	0.419	-0.052	0.146	0.408	-0.02	0.705	0.426	-0.018	0.534	-0.079	**1.72E-10**	48	0.085
rs11509199	T	0.47	0.432	-0.114	4.96E-05	0.435	-0.099	4.41E-07	0.403	-0.122	0.009	0.419	-0.052	0.146	0.408	-0.02	0.705	0.426	-0.018	0.535	-0.079	**1.72E-10**	48	0.085

Variant			Generation R			ALSPAC			GOOD			RS-III			RS-II			RS-I			Combined			
SNP	A1	R2*	Freq.	BETA**	P	Freq	BETA**	P	Freq	BETA**	P	Freq	BETA**	P	Freq	BETA**	P	Freq	BETA**	P	BETA**	P	I2	HetP
rs12706333	T	0.47	0.434	-0.114	5.29E-05	0.435	-0.099	4.64E-07	0.403	-0.122	0.009	0.419	-0.052	0.146	0.408	-0.02	0.707	0.426	-0.018	0.530	-0.079	**1.72E-10**	48	0.085
rs10266975	C	0.47	0.434	-0.114	5.35E-05	0.435	-0.099	4.71E-07	0.403	-0.122	0.009	0.419	-0.052	0.146	0.408	-0.019	0.708	0.426	-0.018	0.529	-0.079	**1.77E-10**	49	0.084
rs17536644	G	0.34	0.419	0.093	0.001	0.432	0.111	**1.77E-08**	0.499	0.054	0.248	0.455	0.045	0.206	0.465	0.081	0.115	0.444	0.029	0.316	0.079	**1.87E-10**	28	0.224
rs6948725	C	0.47	0.440	-0.114	4.54E-05	0.435	-0.098	4.92E-07	0.403	-0.121	0.009	0.419	-0.052	0.146	0.408	-0.019	0.710	0.426	-0.019	0.525	-0.079	**2.04E-10**	47	0.093
rs10225276	G	0.47	0.434	-0.113	5.40E-05	0.435	-0.098	4.89E-07	0.403	-0.122	0.009	0.419	-0.052	0.146	0.408	-0.019	0.709	0.426	-0.019	0.526	-0.078	**2.18E-10**	47	0.095
rs12539571	C	0.47	0.434	-0.113	5.91E-05	0.435	-0.098	5.29E-07	0.402	-0.121	0.009	0.419	-0.052	0.146	0.407	-0.019	0.713	0.426	-0.019	0.522	-0.078	**2.26E-10**	47	0.096
rs10251901	G	0.38	0.413	0.07	0.013	0.413	0.107	4.31E-08	0.460	0.101	0.028	0.414	0.064	0.078	0.423	0.086	0.097	0.406	0.024	0.412	0.078	**2.30E-10**	18	0.299
rs6970383	C	0.47	0.435	-0.114	5.17E-05	0.438	-0.096	8.00E-07	0.404	-0.118	0.011	0.419	-0.052	0.150	0.408	-0.019	0.720	0.426	-0.019	0.522	-0.078	**3.39E-10**	45	0.104
rs4731007	G	0.47	0.437	-0.114	4.74E-05	0.438	-0.096	8.69E-07	0.404	-0.117	0.012	0.419	-0.051	0.154	0.409	-0.018	0.726	0.427	-0.019	0.523	-0.077	**3.40E-10**	46	0.101
rs6947934	C	0.47	0.437	-0.114	4.82E-05	0.438	-0.096	8.57E-07	0.404	-0.117	0.012	0.419	-0.051	0.153	0.408	-0.018	0.724	0.427	-0.019	0.523	-0.077	**3.40E-10**	46	0.101
rs3801385	C	0.00	0.101	-0.172	1.96E-04	0.105	-0.112	4.25E-04	0.101	-0.181	0.017	0.087	-0.088	0.151	0.100	-0.149	0.070	0.098	-0.101	0.035	-0.126	**3.86E-10**	0	0.784
rs11770502	G	0.41	0.453	-0.113	9.01E-05	0.450	-0.1	5.42E-07	0.423	-0.127	0.008	0.437	-0.042	0.258	0.426	-0.019	0.720	0.447	-0.017	0.575	-0.078	**4.48E-10**	50	0.074
rs12706321	G	0.07	0.034	-0.231	0.007	0.042	-0.251	3.50E-05	0.042	-0.101	0.474	0.045	-0.189	0.066	0.045	-0.254	0.126	0.047	-0.221	0.003	-0.223	**5.93E-10**	0	0.953
rs13226812	G	0.07	0.035	-0.234	0.007	0.042	-0.251	4.23E-05	0.042	-0.106	0.462	0.046	-0.175	0.095	0.046	-0.214	0.207	0.048	-0.223	0.004	-0.221	**1.30E-09**	0	0.954
rs10261386	T	0.27	0.384	0.074	0.010	0.390	0.102	3.72E-07	0.455	0.069	0.132	0.407	0.063	0.080	0.413	0.063	0.227	0.399	0.022	0.466	0.073	**4.14E-09**	6	0.378
rs17143147	T	0.39	0.403	0.076	0.010	0.443	0.102	1.53E-07	0.521	0.058	0.211	0.469	0.044	0.210	0.476	0.062	0.222	0.457	0.011	0.691	0.069	**1.01E-08**	35	0.176
rs6962509	A	0.14	0.445	0.076	0.007	0.424	0.099	5.98E-07	0.479	0.064	0.155	0.443	0.061	0.085	0.449	0.022	0.670	0.435	0.025	0.393	0.070	**1.28E-08**	9	0.359
rs10275526	C	0.14	0.440	0.072	0.010	0.424	0.099	5.35E-07	0.479	0.065	0.150	0.444	0.061	0.084	0.449	0.022	0.670	0.435	0.025	0.384	0.069	**1.78E-08**	8	0.365
rs2192291	T	0.14	0.427	0.073	0.010	0.424	0.099	6.38E-07	0.478	0.063	0.161	0.443	0.061	0.085	0.448	0.022	0.670	0.435	0.024	0.400	0.069	**1.90E-08**	10	0.351
rs12666340	C	0.14	0.443	0.071	0.012	0.424	0.099	5.49E-07	0.479	0.065	0.150	0.444	0.061	0.084	0.449	0.022	0.670	0.435	0.025	0.384	0.069	**1.95E-08**	8	0.365
rs12666345	G	0.14	0.442	0.07	0.012	0.424	0.099	5.38E-07	0.479	0.065	0.150	0.444	0.061	0.084	0.449	0.022	0.670	0.435	0.025	0.384	0.069	**2.13E-08**	8	0.366
rs12672958	C	0.14	0.442	0.07	0.012	0.424	0.099	5.57E-07	0.479	0.065	0.150	0.444	0.061	0.084	0.449	0.022	0.670	0.435	0.025	0.384	0.069	**2.13E-08**	8	0.366
rs11977285	A	0.14	0.442	0.07	0.012	0.424	0.099	6.04E-07	0.479	0.064	0.157	0.443	0.061	0.085	0.448	0.022	0.670	0.435	0.025	0.395	0.069	**2.21E-08**	8	0.365
rs2402555	T	0.14	0.442	0.07	0.012	0.424	0.099	6.19E-07	0.478	0.064	0.158	0.443	0.061	0.085	0.448	0.022	0.670	0.435	0.025	0.396	0.069	**2.21E-08**	8	0.365
rs798905	C	0.06	0.346	-0.124	3.66E-05	0.260	-0.06	7.04E-03	0.232	-0.093	0.098	0.244	-0.047	0.260	0.250	-0.146	0.018	0.252	-0.047	0.157	-0.076	**3.51E-08**	12	0.336
rs798913	A	0.02	0.073	0.11	0.056	0.081	0.156	1.66E-05	0.075	0.074	0.407	0.068	0.132	0.060	0.068	-0.046	0.672	0.071	0.157	0.009	0.130	**4.81E-08**	0	0.573
rs2691034	G	0.07	0.329	-0.123	7.59E-05	0.266	-0.063	0.005	0.237	-0.09	0.107	0.248	-0.044	0.293	0.255	-0.144	0.020	0.256	-0.046	0.172	-0.076	**5.38E-08**	5	0.384

Shaded rs917727 top-hit for GW- association with TB-BMD and rs4609139 top-hit of the secondary signal in TB-BMD *Correlation coefficients with rs7801723 based on HapMap release22 CEU population. **Effect estimates expressed as standardized adjusted SD per copy of allele (A1). Underline: rs7776725 Top-hit wrist fracture GWAS, rs2536189 Top-hit forearm BMD GWAS, rs2707466 Top-hit for Cortical thickness in Zheng et al (accompanying submission).
