# Supplementary material for: Meta-Analysis of Genome-Wide Scans for Total Body BMD in Children and Adults Reveals Allelic Heterogeneity and Age-Specific Effects at the WNT16 Locus
Source: PLoS Genet. 2012 Jul 5;8(7):e1002718. doi: 10.1371/journal.pgen.1002718 (PMC3390371; doi:10.1371/journal.pgen.1002718)
Supplement: Table S6 — Fam3c KO mouse data for each knockout strategy. Gene trap and two types of homologous recombination: 1 and 2 (HR #1, HR #2). (PDF) [file pgen.1002718.s009.pdf]

	KO	Male WT	Male KO	Female WT	Female KO
**Number of Mice**	Gene Trap	2	3	2	3
	HR #1	2	4	2	4
	HR #2	2	4	2	4
**Body Weight (grams)**	Gene Trap	33.9 ± 1.8	35.9 ± 1.4	20.4 ± 2.5	24.7 ± 1.5
	HR #1	27.1 ± 3.4	29.5 ± 2.6	22.7 ± 1.7	23.2 ± 1.3
	HR #2	33.0 ± 2.7	26.6 ± 1.4	20.6 ± 1.2	23.9 ± 0.7
**LBM (grams)**	Gene Trap	25.2 ± 1.2	26.2 ± 1.1	14.9 ± 1.7	17.2 ± 0.5
	HR #1	21.0 ± 1.9	22.9 ± 1.7	17.3 ± 0.8	17.0 ± 0.9
	HR #2	24.2 ± 1.6	20.7 ± 1.1	16.4 ± 1.1	18.4 ± 0.4
**Body Fat (percent)**	Gene Trap	21.4 ± 1.3	24.1 ± 1.3	20.9 ± 2.5	26.6 ± 5.6
	HR #1	18.3 ± 3.8	19.0 ± 1.9	19.7 ± 2.4	22.5 ± 3.5
	HR #2	26.0 ± 2.3	21.5 ± 1.1	17.2 ± 0.0	20.4 ± 2.8
**Body aBMD (mg/cm2)**	Gene Trap	53.6 ± 2.5	53.6 ± 0.8	48.3 ± 0.7	49.5 ± 0.8
	HR #1	51.4 ± 3.9	51.8 ± 1.9	47.3 ± 1.3	47.0 ± 1.3
	HR #2	51.4 ± 0.1	50.5 ± 1.4	47.6 ± 0.9	50.9 ± 0.9
**Body Bone Area (cm2)**	Gene Trap	10.0 ± 0.2	10.0 ± 0.4	9.4 ± 0.2	9.0 ± 0.7
	HR #1	9.5 ± 0.3	9.4 ± 0.2	8.5 ± 0.1	8.8 ± 0.3
	HR #2	8.7 ± 0.0	8.9 ± 0.4	8.8 ± 0.6	8.8 ± 0.2
**Body BMC (mg)**	Gene Trap	533 ± 35	537 ± 24	452 ± 18	447 ± 40
	HR #1	485 ± 19	486 ± 21	404 ± 17	414 ± 25
	HR #2	446 ± 3	450 ± 28	421 ± 35	450 ± 17
**Femur aBMD (mg/cm2)**	Gene Trap	84.8 ± 4.6	85.1 ± 4.5	64.9 ± 1.7	72.8 ± 2.4
	HR #1	76.8 ± 3.2	78.9 ± 4.1	71.6 ± 0.1	69.8 ± 0.9
	HR #2	74.8 ± 0.3	80.1 ± 3.4	72.7 ± 2.0	77.2 ± 1.1
**Femur Bone Area (cm2)**	Gene Trap	0.40 ± 0.01	0.40 ± 0.01	0.37 ± 0.05	0.36 ± 0.02
	HR #1	0.38 ± 0.03	0.37 ± 0.01	0.33 ± 0.02	0.33 ± 0.01
	HR #2	0.39 ± 0.01	0.33 ± 0.02	0.33 ± 0.01	0.36 ± 0.01
**Femur BMC (mg)**	Gene Trap	33.9 ± 2.8	34.3 ± 1.8	24.0 ± 2.8	26.3 ± 1.1
	HR #1	29.1 ± 3.3	29.0 ± 2.3	23.5 ± 1.7	22.9 ± 0.9
	HR #2	29.1 ± 1.0	26.4 ± 2.7	23.9 ± 1.5	27.3 ± 0.7
**Spine aBMD (mg/ cm2)**	Gene Trap	68.5 ± 1.3	62.8 ± 0.9	63.6 ± 1.6	63.6 ± 0.4
	HR #1	58.5 ± 5.6	62.7 ± 1.9	54.8 ± 1.9	52.8 ± 3.6
	HR #2	56.2 ± 4.8	59.5 ± 2.7	53.8 ± 4.0	65.0 ± 3.6
**Spine BMC (mg)**	Gene Trap	31.4 ± 0.8	29.6 ± 0.8	28.4 ± 0.4	26.8 ± 1.2
	HR #1	26.6 ± 3.2	27.4 ± 0.6	23.6 ± 0.6	21.4 ± 0.9
	HR #2	22.9 ± 1.6	26.2 ± 1.0	23.7 ± 1.1	29.0 ± 2.0

Results provided as [mean +/- SEM]
