# Supplementary material for: Meta-Analysis of Genome-Wide Scans for Total Body BMD in Children and Adults Reveals Allelic Heterogeneity and Age-Specific Effects at the WNT16 Locus
Source: PLoS Genet. 2012 Jul 5;8(7):e1002718. doi: 10.1371/journal.pgen.1002718 (PMC3390371; doi:10.1371/journal.pgen.1002718)
Supplement: Table S7 — SNPs showing association with TB-BMD in the discovery cohort, overall and by ethnic clustering. (PDF) [file pgen.1002718.s010.pdf]

					All Generation R participants												
					Combined Generation R (n=2,660)			Generation R CEU (n=1,511)			Generation R Non-CEU						
		CEU	YRI	JPT/CHB							Overall (n=1,149)			CLUSTER 1 (n=263)		CLUSTER 2 (n=866)	
SNP	A1	R2*	R2*	R2*	Freq.	BETA**	P	Freq	BETA**	P	Freq	BETA**	P	BETA**	P	BETA**	P
**rs917727**	T	1	1	1	0.296	0.21	**4.11E-11**	0.263	0.233	1.30E-07	0.32	0.183	9.19E-05	0.214	0.03	0.173	0.001
rs2908004	A	0.55	0	0.563	0.501	0.16	**1.23E-08**	0.457	0.178	1.59E-06	0.526	0.131	0.001	0.156	0.094	0.127	0.009
rs917726	T	1	0.74	1	0.282	0.208	**6.42E-11**	0.256	0.231	1.34E-07	0.3	0.178	1.32E-04	0.149	0.123	0.182	0.001
rs718766	C	1	0.51	1	0.272	0.208	**8.63E-11**	0.253	0.232	1.17E-07	0.283	0.175	2.05E-04	0.084	0.397	0.189	0.001
rs3801382	G	1	0.51	0.778	0.275	0.199	**1.04E-10**	0.256	0.224	1.16E-07	0.287	0.165	2.64E-04	0.072	0.448	0.182	0
rs7776725	*C*	*1*	*0.38*	*1*	*0.264*	*0.214*	***4.58E-11***	*0.251*	*0.233*	*1.46E-07*	*0.27*	*0.188*	*1.02E-04*	*0.13*	*0.204*	*0.19*	*0.001*
rs2536189	*G*	*0.55*	*0.01*	*0.563*	*0.498*	*0.153*	***2.23E-08***	*0.455*	*0.177*	*1.71E-06*	*0.521*	*0.126*	*0.002*	*0.15*	*0.107*	*0.123*	*0.011*
rs3801387	G	1	0.32	0.778	0.274	0.197	**1.61E-10**	0.255	0.223	1.24E-07	0.287	0.162	3.25E-04	0.004	0.968	0.187	0
rs4727924	T	0.513	0.25	0.641	0.467	0.172	**3.88E-09**	0.444	0.19	1.11E-06	0.474	0.155	4.84E-04	0.242	0.011	0.129	0.012
rs2536182	G	0.531	0	0.778	0.471	0.156	**2.69E-08**	0.443	0.186	7.23E-07	0.484	0.117	0.006	0.123	0.194	0.112	0.024
rs2536180	C	0.513	0.005	0.563	0.495	0.143	1.57E-07	0.464	0.172	2.70E-06	0.509	0.11	0.008	0.092	0.323	0.111	0.02
rs2707466	*T*	*0.511*	*0.01*	*0.563*	*0.485*	*0.152*	***3.55E-08***	*0.443*	*0.181*	*1.36E-06*	*0.51*	*0.118*	*0.004*	*0.139*	*0.134*	*0.116*	*0.018*
rs2254595	C	0.513	0.019	0.641	0.504	0.142	1.20E-07	0.469	0.166	4.90E-06	0.521	0.117	0.004	0.132	0.144	0.111	0.019
rs3779381	G	0.868	0.323	0.778	0.264	0.18	**1.57E-08**	0.241	0.197	7.31E-06	0.279	0.156	7.53E-04	-0.007	0.944	0.184	0.001
rs2536150	C	0.068	0.005	0	0.215	-0.136	6.22E-05	0.185	-0.166	5.08E-04	0.231	-0.114	0.02	-0.029	0.759	-0.125	0.03
rs2952559	C	0.055	0.001	0	0.265	-0.133	6.34E-05	0.211	-0.191	8.59E-05	0.306	-0.096	0.04	-0.118	0.2	-0.094	0.095
rs13247600	C	0.039	Monomorphic	Monomorphic	0.064	-0.219	7.07E-04	0.084	-0.189	0.013	0.043	-0.347	0.005	-0.471	0.302	-0.3	0.02
rs2707520	C	0.249	Monomorphic	0.053	0.444	-0.091	0.001	0.505	-0.093	0.013	0.386	-0.096	0.026	-0.114	0.275	-0.084	0.084
rs17509082	T	0.386	0.005	0.22	0.173	0.119	0.001	0.193	0.167	4.47E-04	0.171	0.048	0.386	0.074	0.547	0.018	0.767
rs2908007	A	0.37	0.028	0.083	0.492	-0.1	4.12E-04	0.57	-0.067	0.092	0.434	-0.133	1.77E-03	-0.151	0.137	-0.139	0.004

Bolded rs917727 top-hit * Correlation coefficients with rs917727 based on HapMap release22 Three different panel populations. **Effect estimates expressed as standardized adjusted SD per copy of allele (A1). Underline: rs7776725 Top-hit wrist fracture GWAS, rs2536189 Top-hit forearm BMD GWAS, rs2707466 Top-hit for Cortical thickness all reported in Zheng et al (accompanying submission).
