# Supplementary material for: Meta-Analysis of Genome-Wide Scans for Total Body BMD in Children and Adults Reveals Allelic Heterogeneity and Age-Specific Effects at the WNT16 Locus
Source: PLoS Genet. 2012 Jul 5;8(7):e1002718. doi: 10.1371/journal.pgen.1002718 (PMC3390371; doi:10.1371/journal.pgen.1002718)
Supplement: Table S8 — Information of genetic data for each of the study cohorts. Genotyping methods, quality control of SNPs, imputation, and statistical analysis for the genome-wide association studies. (PDF) [file pgen.1002718.s011.pdf]

Cohort	Platform	Genotyping				SNPs that met QC criteria	Imputation			Association analyses			
		Genotype calling algorithm	Inclusion criteria				Imputation software	Inclusion criteria		Analyses software	No. analyzed SNPs	men	women
			MAF	Call rate	p for HWE			MAF	Imputation quality				
GENERATION R	Illumina HumanHap 550K Quad	Genome Studio	≥ 1%	≥ 98%	> 10-6	469,644	MACH/minimac	≥1%	MACH R2 ≥ 0.3	MACH2QTL via GRIMP	3,021,329	1,313	1,347
ALSPAC	Illumina HumanHap 550K Quad	Genome Studio	≥ 1%	≥ 97%	>5x10-7	464,311	MACH	≥1%	MACH R2 ≥ 0.3	MACH2QTL	2,543,887	2,647	2,787
GOOD	Illumina / HumanHap 610 Quad	Beadstudio Genecall	≥ 1%	≥ 97.5%	> 10-6	521,160	MACH	≥1%	MACH R2 ≥ 0.3	MACH2QTL via GRIMP	2,543,887	938	0
RS-III	Illumina / HumanHap 610 QUAD	Genome Studio	≥ 1%	≥ 97.5%	> 10-6	514,073	MACH	≥1%	MACH R2 ≥ 0.3	MACH2QTL via GRIMP	2,543,887	680	914
RS-II	Illumina / HumanHap 550 V.3 DUO; Illumina / HumanHap 610 QUAD	Genome Studio	≥ 1%	≥ 97.5%	> 10-6	466,389	MACH	≥1%	MACH R2 ≥ 0.3	MACH2QTL via GRIMP	2,543,887	345	405
RS-III	Illumina / HumanHap 610 QUAD	Genome Studio	≥ 1%	≥ 97.5%	> 10-6	514,073	MACH	≥1%	MACH R2 ≥ 0.3	MACH2QTL via GRIMP	2,543,887	1000	594
RS-I	Illumina / HumanHap 550K V.3 ADHumanHap 550 V.3 DUO;	Beadstudio Genecall	≥ 1%	≥ 97.5%	> 10-6	512,349	MACH	≥1%	MACH R2 ≥ 0.3	MACH2QTL via GRIMP	2,448,227	1,051	1,385
